# High-temperature water unlocks urea as nitrogen-source towards imidazoles†

**DOI:** 10.1039/d4gc01705f

**Published:** 2024-09-20

**Authors:** Fabián Amaya-García, Lena Schittenhelm, Miriam M. Unterlass

**Affiliations:** a Universität Konstanz, Department of Chemistry Universitätsstrasse 10 78464 Konstanz Germany fabian.amaya-garcia@uni-konstanz.de miriam.unterlass@uni-konstanz.de; b CeMM Research Center for Molecular Medicine of the Austrian Academy of Sciences Lazarettgasse 14 AKH BT25.3 1090 Vienna Austria

## Abstract

Urea is a non-toxic, harmless, and abundant bulk organic chemical featuring high nitrogen content. Therefore, urea could be a prime green candidate for introducing nitrogen atoms into organic molecules. In this regard, urea in organic synthesis has been mainly employed as building block, component of solvent systems, catalyst, or for pH adjustment, while uses of urea as NH_3_-source towards the construction of small organic compounds are scarce. Here, nothing but high-temperature water (HTW) is employed to conduct the aquathermolysis of urea, generating NH_3_ to propel the Debus–Radzsisweski multicomponent reaction (MCR) towards imidazoles. The approach does neither require additional catalysts nor reaction auxiliaries or volatile organic compounds as solvent. Urea was used as N-source in combination with different 1,2-diketones and aldehydes featuring a variety of functional groups towards 23 lophine analogues (190 °C, 1–3 hours). Moreover, the presented synthesis performs equally or better than classical syntheses when acid-sensitive substrates are employed. The greenness of the synthesis using urea in HTW was assessed through green metrics and compared with syntheses reported in the literature in a large-scale fashion. Overall, the reported syntheses feature *E*-factor and process mass intensity values in ranges comparable to those of syntheses reported in literature.

## Abstract 2

The synergy between high-temperature water and urea propels the synthesis of imidazoles via the Debus-Radziszewski multicomponent reaction in a catalys-free fashion.

## Graphical abstract

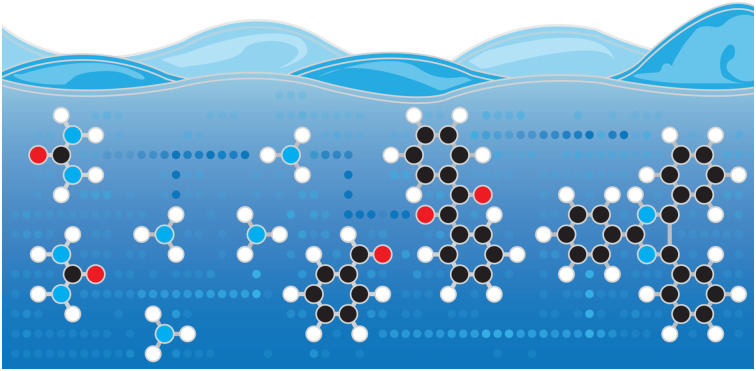

## Introduction

Urea, CO(NH_2_)_2_, is a high production volume organic chemical (>150 Mt a^−1^ in 2010) with a pivotal role in the chemical industry.^1,2^ Besides featuring high nitrogen content, urea is abundant, non-toxic, chemically stable under conventional storage conditions, and does not represent handling risks, such as high volatility or flammability. Consequently, urea exhibits interesting features from a green chemistry perspective. Urea is the most frequently used nitrogen fertilizer. In presence of ureases, the enzymatic hydrolysis of urea takes place and selectively produces NH_3_ and H_2_CO_3_,^3^ which is well-known as the *modus operandi* of urea fertilizers. Surprisingly, the seemingly straightforward use of urea as N-source towards the construction of well-defined organic molecules has not been actively explored in organic synthesis. To date, uses of urea are limited to a handful of roles, including those as component in deep eutectic solvent systems,^4^ pH adjustment,^5^ or as consumed participant towards urea-formaldehyde resins,^2^ heterocycles produced through multicomponent reactions (MCRs), *e.g.*, the Biginelli MCR,^6^ or melamine-derived materials (*e.g.*, carbon nitride C_3_N_4_).^7^ The synthesis of the latter compounds employs urea as direct starting material without using it as NH_3_-source.

**Scheme 1:**
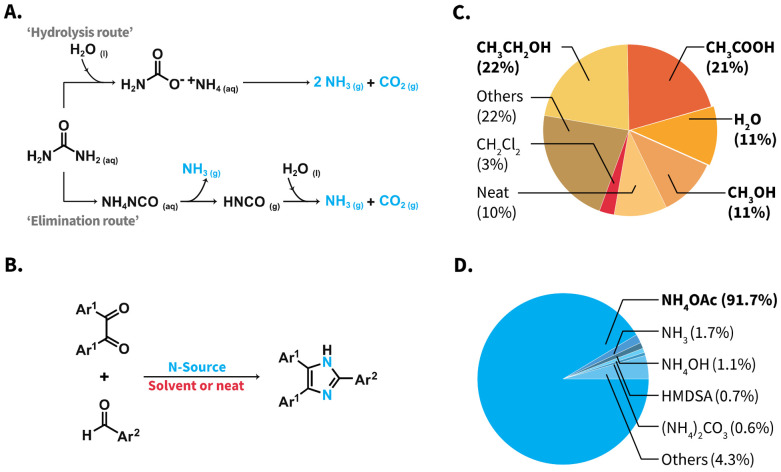
(A) Aquathermolysis of urea towards NH_3_ and CO_2_*via* ‘hydrolysis route’ (top) and ‘elimination route’ (bottom). (B) Reaction equation of the Debus–Radzisewski MCR to 2,4,5-trisubstituted imidazoles, reported reaction media (C) and reported N-sources (D). The pie charts show results of a database (Reaxys^TM^) search of syntheses towards 2,4,5-trisubstituted imidazoles (see ESI for details†). NH_4_OAc: ammonium acetate; HMDSA: hexamethyldisilazane, “Others” in Plot C refers to 5 different N-sources, individually covering 0.1–0.5% and in Plot D to 55 media covering 0.02–3%.

Besides enzymatic hydrolysis, urea can be heated up in the solid state (pyrolysis),^8^ or treated *via* electrochemical methods to generate NH_3_.^9,10^ These methods exhibit inherent drawbacks to construct small organic molecules. The enzymatic hydrolysis proceeds in aqueous environments that are generally incompatible with low-polarity, and hence water insoluble starting materials. Electrochemical methods generally require further oxidizing agents or catalysts, which lead to additional products besides NH_3_.^9,10^ Pyrolysis of urea has been used to generate NH_3_ in a handful of examples.^11^ This method nevertheless entails a complex network of reactions and generates byproducts as a function of the heating temperature, *e.g.*, biuret, cyanuric acid, and ammelide, are produced besides NH_3_ at 190 °C.^12^ Alternatively to these methods, high-temperature water (HTW) can be used to perform the aquathermolysis of urea.^10,13^ No further catalysts or reaction auxiliaries are required, and the approach ultimately produces NH_3_ and CO_2_ exclusively (Scheme 1A). Literature points at the so-called ‘hydrolysis route’ or an ‘elimination route’ to generate these products.^13,14^ To date, generating NH_3_*in situ via* aquathermolysis of urea has received attention for developing selective catalytic reduction systems in the automotive industry, treatment of flue gases, valorization of urea-containing wastewaters at industrial scale, and pH adjustment in the hydrothermal synthesis of, *e.g.*, metal oxide nanostructures.^5,10,15,16^ However, NH_3_ generated *via* urea aquathermolysis has not been explored in organic synthesis at preparative scale.

In this contribution, we show that HTW is a unique reaction medium to conduct the aquathermolysis of urea in tandem with the synthesis of heteroaromatic compounds. In particular, HTW remarkably allows for conducting both urea aquathermolysis and the Debus–Radzisewski MCR without further catalysts, reaction auxiliaries, and volatile organic compounds as reaction media. Liquid H_2_O is abundant, non-toxic, non-flammable, and in superheated form has in recent years been shown to be a potent solvent for organic synthesis. Compared with r.t. H_2_O, HTW exhibits (i) decreased static dielectric constant (*ε*), hence decreased polarity,^17^ and (ii) increased ionic product that reflects H_2_O's enhanced Brønsted acido-basic character.^18^ This interplay between polarity and acido-basic character is expected to influence the overall greenness of reactions. Reaction solvents have been identified as major contributors to the generation of waste from a chemical synthesis.^19^ In response to this, countermeasures such as the restriction of toxic organic solvents and research on alternative reaction media are encouraged, while the chemical industry has developed solvent selection guides to minimize waste generation.^20^ HTW allows for solubilizing low-polarity starting materials, and hence prevents waste generation from the usage of volatile organic compounds as solvents. Moreover, upon cooling down to r.t., H_2_O recovers its characteristic polarity and reaction products phase separate. This generally facilitates filtration as straightforward method to isolate the products. These features for both synthesis at preparative scale and purification have been explored towards a handful of heteroaromatic compounds.^21–23^

The Debus–Radziszewski MCR is a well-established synthesis that employs 1,2-diketones, aldehydes, and a N-source as starting materials towards imidazole-containing compounds (Scheme 1B).^24^ These compounds are highly relevant for applications in materials and life sciences.^25^ To date, research on the Debus–Radziszewski MCR has heavily focused on developing new reaction catalysts. Nevertheless, implementing catalysts in routine syntheses relies on the catalysts’ availability, often involving further reaction steps and/or laborious synthetic procedures. This reduces the overall greenness of otherwise efficient MCRs. In contrast, catalyst-free syntheses are expected to overcome this drawback.

More interestingly, the vast majority of reported Debus–Radzisewski syntheses have in fact been performed employing a rather limited number of reaction media and N-sources. In terms of reaction media, reported syntheses have been mainly conducted in alcohols and acetic acid (HOAc), summing up to 57% of reported syntheses (Scheme 1C). While several contributions have used H_2_O as reaction medium (Scheme 1C, 11% of reported syntheses), only alkyl-substituted imidazoles and imidazolium salts have been achieved in r.t. H_2_O.^26,27^ The vast majority of syntheses towards imidazoles featuring low-polarity, *e.g.*, 2,4,5-triarylimidazoles aka lophines, in H_2_O typically require catalysts, organic cosolvents, *e.g.*, methanol, ethanol, acetonitrile, or combinations thereof (ESI, Table S4†).

As N-source, NH_4_OAc has been by far the most used compound, accounting for 91.7% of the reported Debus–Radzisewski syntheses (Scheme 1D and ESI†). Other ammonium salts, *e.g.*, ammonium carbonate (0.6%), or the organosilicon compound hexamethyldisilazane (HMDSA; 0.7%) have been reported.^28,29^ Marestin *et al.* provided a synthesis of lophines in HTW using NH_4_OAc (*T* = 210 °C within 5–10 minutes for 9 compounds in yields from 74 to 99%).^30^ This example showcases the promising role of HTW as medium to synthesize lophines. Nevertheless, the synthesis features a scope with low functional group variety and loses performance when low substrate concentrations are employed. The latter is particularly problematic for expensive, scarce, or high-molecular weight starting materials. Urea as N-source is abundant, non-toxic, and as alternative to NH_4_OAc, odorless and non-hygroscopic. Reported Debus–Radziszewski syntheses have employed urea as part of deep eutectic solvent systems with choline acetate^31^ or ZnCl_2_,^32^ and in a solvent system with hydrogen peroxide.^33^ All these syntheses still use NH_4_OAc as N-source. Only one report deals with the ultrasound-assisted Debus–Radzisewski syntheses using urea, yet using triphenylphosphine as reaction auxiliar in alcohols.^34^ In summary, the Debus–Radzisewski MCR towards lophines strongly relies on reaction catalysts or auxiliaries, organic solvents, and is limited to NH_4_OAc as benchmark N-source. Overall, the use of alternative N-sources is underexplored and to the best of our knowledge, urea has not been used as N-source in a catalyst-free, organic solvent-free fashion.

Here, we show that HTW as reaction medium unlocks the use of urea as N-source to propel the Debus–Radzisewski MCR towards lophines without relying on further catalysts or reaction auxiliaries. More interestingly, commonly used organic solvents for performing the Debus–Radzisewski MCR are not completely compatible with the use of urea as N-source. As we show herein, switching from HTW to organic solvents goes beyond a mere change of reaction media, and in fact influences the reactions’ performance.

## Results and discussion

**Scheme 2:**
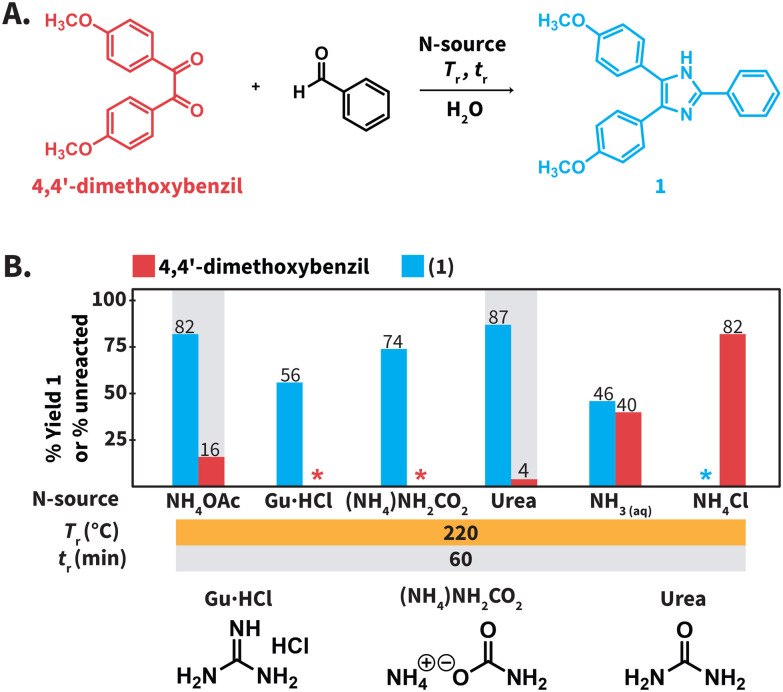
(A) Model reaction to synthesize compound 1 in HTW. (B) Screening of N-sources. Experiments performed using 0.3 mmol of 4,4′-dimethoxybenzil and benzaldehyde, 3.0 mmol of N-source, in 3.0 mL of distilled H_2_O. Yields (%) of 1 and amounts (%) of unreacted 4,4′-dimethoxybenzil were determined *via*^1^H NMR spectroscopy with dimethyl sulfone as internal standard. *The compound was not detected. Gray background indicates that the experiment was performed twice and we show the average values.

To investigate the Debus–Radziszewski MCR in HTW, we selected the model reaction between 4,4′-dimethoxybenzil and benzaldehyde towards 4,5-bis(4-methoxyphenyl)-2-phenylimidazole (1) (Scheme 2A). The majority of experiments were conducted with a 1 : 1 : 10 molar ratio of 4,4′-dimethoxybenzil : benzaldehyde : N-source, since this is a widely used ratio in reported Debus–Radziszewski syntheses. The model reaction was performed at different reaction temperatures (*T*_r_) and reaction times (*t*_r_) in a microwave (MW) assisted fashion. The crude reaction products were analyzed *via*^1^H NMR spectroscopy to determine the outcome of the reaction (see ESI for experimental details and Table S5 for performed reactions†).

### Screening of N-sources in HTW to conduct the Debus–Radziszewski MCR

We first screened the compatibility of different N-sources with HTW to synthesize compound 1 (Scheme 2B). In a typical Debus–Radziszewski synthesis, NH_4_OAc releases HOAc and NH_3_, the latter supplying nitrogen to form the imidazole ring. We envisioned that alternative compounds could act as N-sources in HTW. To test this, we investigated guanidinium hydrochloride (Gu·HCl), ammonium carbamate ((NH_4_)NH_2_CO_2_), ammonium chloride (NH_4_Cl), aqueous NH_3_, ammonium carbonate (NH_4_)_2_CO_3_, and urea as N-sources. For comparison, we also tested NH_4_OAc, *i.e.*, the benchmark N-source. The formation of 1 was used to establish whether the compounds indeed acted as N-source to form the imidazole ring in HTW. We performed the model reaction for *t*_r_ = 60 min using 10 equiv. of the tested N-sources. The model reaction was conducted at *T*_r_ = 220 °C for all the N-sources. The selected *T*_r_ is close to the maximum temperature recommended by the employed MW reactor (230 °C for H_2_O). We expected that these parameters were sufficient to avoid biased results owing to short *t*_r_, low *T*_r_, or insufficient equiv. of N-source.

All the reactions produced mixtures mainly composed of the starting material 4,4′-dimethoxybenzil and compound 1 (Scheme 2B). Note that benzaldehyde was not detected. We hypothesize that benzaldehyde is either reacting towards further products under the employed conditions or is not recovered during the isolation of the crude products. The model reaction using NH_4_OAc produced 82% of 1 and 16% of unreacted 4,4′-dimethoxybenzil. This indicated that the formation of the imidazole ring could take place under the selected conditions. The reaction using NH_4_Cl did not yield detectable amounts of 1. Using aqueous NH_3_ only resulted in 46% of 1, while reactions using Gu·HCl and ((NH_4_)NH_2_CO_2_) yielded 56% and 74%, respectively. For the model reaction using (NH_4_)_2_CO_3_, we observed an uncontrolled increase of pressure over *t*_r_. The maximum pressure allowed by the set-up was reached even below *T*_r_ = 150 °C (see ESI, section 3†). Therefore, this experiment was interrupted for safety reasons. The model reaction using urea generated the highest yield of 1 (87%). While (NH_4_)_2_CO_3_ could be formed after the generation of NH_3_ and CO_2_ from urea in HTW, the observed pressure for the experiment using urea was more controlled. Among the tested N-sources, using urea resulted in the best balance between yield of 1, stability of the N-source, and controlled increase of pressure over *t*_r_.

### Screening of reaction parameters using urea in HTW

**Scheme 3:**
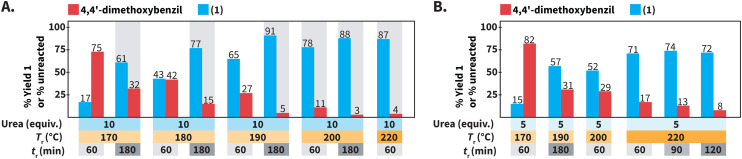
Screening of reaction conditions for synthesizing of 1 using urea as N-source. (A) Influence of *T*_r_ and *t*_r_. (B) Experiments using 5 equiv. of urea. Experiments were performed using 0.3 mmol of 4,4′-dimethoxybenzil and benzaldehyde, 3.0 mmol (10 equiv.) or 1.5 mmol (5 equiv.) of urea, in 3.0 mL of distilled H_2_O. Yields (%) of 1 and amounts (%) of unreacted 4,4′-dimethoxybenzil were calculated *via*^1^H NMR spectroscopy with dimethyl sulfone as internal standard. Gray background indicates that the experiment was performed twice and we show the average values.

We further studied the model reaction towards 1 in HTW using urea (Scheme 3). We screened the following reaction parameters: 170 °C ≤ *T*_r_ ≤ 220 °C, 60 min ≤ *t*_r_ ≤ 180 min, equiv. of urea = 5 and 10, and *c*_M_ = 0.1 mol L^−1^. Note that we tested specific combinations of these parameters rather than testing all possible combinations.

We first focused on the employed *T*_r_ and *t*_r_ for synthesizing compound 1 (Scheme 3A). We have observed that decreasing the *T*_r_ for synthesizing quinoxalines, *i.e.*, a heteroaromatic scaffold, in HTW can be paired with prolonged *t*_r_ values to compensate for drops in reaction yields.^35^ Thus, we tested whether the formation of 1 could still take place with high yields at *T*_r_ < 220 °C, at expense of longer values of *t*_r_. Performing the synthesis of 1 at *T*_r_ = 200 °C resulted in 78% of 1 after *t*_r_ = 60 min. Longer *t*_r_ values were expected to increase the yield of 1 at 200 °C and 88% of 1 could be obtained after *t*_r_ = 180 min. Further decreasing the *T*_r_ to 190 °C and 180 °C resulted in yields lower (65% and 43%) than that at 200 °C (78%) after *t*_r_ = 60 min. As expected, prolonging the *t*_r_ to 180 min at values of *T*_r_ < 200 °C increased the yield of 1. Among the screened conditions, the highest yield of 1 at the lowest amount of unreacted 4,4′-dimethoxybenzil was observed at *T*_r_ = 190 °C after *t*_r_ = 180 min.

We next focused on the equivalents of N-source (Scheme 3B). Since all the prior experiments were performed using 10 equiv. of urea, we tested whether less than 10 equiv. could still lead to the formation of 1 in high yields. At 220 °C after 60 min, using 5 equiv. of urea resulted in lower yield (71% of 1) than that using 10 equiv. Moreover, the yield of 1 did not increase with prolonged *t*_r_, but stagnated in the range of 71 to 74%. Overall, using 5 equiv. of urea resulted in yields lower than that using 10 equiv. for reactions at *T*_r_ ≤ 220 °C and different *t*_r_.

In summary, different combinations of parameters can employed in HTW using urea to synthesize 1 with very good to excellent yields. Among the tested conditions, 1 was obtained with the highest yield (91%) at the lowest amount of unreacted 4,4′-dimethoxybenzil (5%) at *T*_r_ = 190 °C after *t*_r_ = 180 min using 10 equiv. of urea at *c*_M_ = 0.1 mol L^−1^ (Scheme 3A).

### Scope of starting materials amenable to the Debus–Radziszewski MCR using urea in HTW

**Scheme 4:**
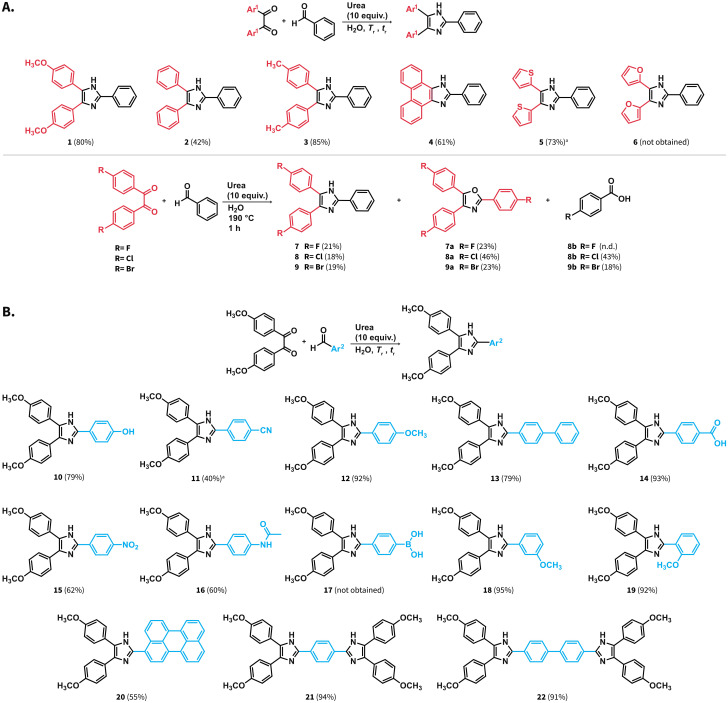
Scope of (A) 1,2-diaryldiketone and (B) aldehyde for the Debus–Radzisewski synthesis of lophines in HTW using urea. Reactions were performed using 0.6 mmol of starting materials and 6.0 mmol of urea in 6.0 mL of distilled H_2_O at *T*_r_ = 190 °C for *t*_r_ = 3 h, ^*a*^*t*_r_ = 1 h.

We investigated the scope of starting materials amenable to the synthesis of lophines in HTW using urea. We selected starting materials bearing a variety of functional groups, thereby aiming at products featuring high chemical diversity at a minimal number of compounds. First, benzaldehyde was reacted with different diketones to obtain compounds 2 to 9 with yields ranging from 18 to 85% (Scheme 4A). Lophine (2) and analogues featuring methyl substituents (3) or the phenanthrene moiety (4) were obtained. We also targeted the synthesis of lophines containing thiophene (5) and furane (6) rings. For these compounds, the employed *t*_r_ was shortened to 1 h since we observed that longer *t*_r_ lead to formation of dark brown products of high polarity. While compound 5 was obtained with 73% yield, compound 6 could not be obtained, suggesting that the substrate 2,2′-furil is not hydrothermally stable at the employed reaction conditions. As it is well-known in the field of hydrothermal carbonization of biomass, (poly-)saccharides first form furaldehydes that subsequently polycondense to carbon materials.^36^ We suspect that similar transformations are responsible for the unsucessful formation of compound 6. Compound 7 substituted with fluorine atoms was obtained with 21% yield after *t*_r_ = 1 h. The reaction resulted in the formation of a complex mixture of high polarity products that were not identified, and the formation of compound 7a. This compound features an oxazole substituted with *p*-fluorophenyl groups exclusively. The structure of compound 7a was confirmed through ^1^H, ^13^C NMR and electrospray ionization high-resolution mass spectrometry (ESI-HRMS). The formation of 7a was unexpected albeit can be explained by side reactions arising from 4,4′-difluorobenzil.^37^ Compound 8 substituted with chlorine atoms was also obtained with low yield (18%) at *T*_r_ = 190 °C after *t*_r_ = 1 h. We observed the formation of the corresponding compound 8a and *p*-chlorobenzoic acid. Analogously, compound 9 substituted with bromine atoms was also obtained with low yields (19%), and the formation of compound 9a and *p*-bromobenzoic acid was observed. The structures of compounds 8a, and 9a were also confirmed through ^1^H, ^13^C NMR and ESI-HRMS. We hypothesize that the formation of 8a and 9a is also justified by side reactions from the corresponding halogenated diketones.

The scope of aryl aldehydes was also explored (Scheme 4B). To this end, 4,4′-dimethoxybenzil was reacted with substituted aldehydes towards lophines bearing hydroxyl (10), methoxy (12), biphenyl (13), carboxylic acid (14), nitro (15), and acetamido (16) groups. Reaction towards compound 11 featuring a cyano substituent yielded the target compound with 40% yield after *t*_r_ = 1 hour at 190 °C. We employed a shorter reaction time since we also observed the generation of compound 14, which starts predominating after prolonged *t*_r_ values. This suggests that the employed reaction conditions promote the hydrolysis of the cyano group either in compound 11 or at the 4-formylbenzonitrile employed as starting material. Compound 17 bearing a phenylboronic acid substituent was not obtained and compound 1 was obtained instead. This suggests that the boronic acid group undergoes protodeboronation under the employed conditions. Compounds with methoxy groups at *meta* (18) and *ortho* (19) position were also obtained. The position of the methoxy substituent did not influence the rection yields for 18 and 19 compared with 12, *i.e.*, the *para* substituted isomer. We further tested the performance of the synthesis of lophines using urea by targeting compounds featuring extended π-conjugation. Despite the presence of a perylene moiety in the aldehyde used as starting material, compound 20 was obtained with 55% yield. Moreover, compounds 21 and 22 featuring imidazoles separated by one and two phenyl rings could be also obtained with 94% and 91% yield, respectively.

In sum, the synthesis of lophines in HTW using urea is amenable with a variety of starting materials. Compounds 13, 16, 20, and 22 have not been previously reported in the Sci-Finder database. The investigated scope indicates that (i) furane rings are not stable under the employed conditions, (ii) halogenated benzils result in yields lower than those of the other studied benzil analogues due to side reactions and, (iii) cyano groups might undergo hydrolysis towards lophines containing carboxylic acid groups.

We tested the applicability of the synthesis at higher scale. The synthesis of compounds 1, 2, 11 and 18 was performed at 1.2 mmol scale (Scheme 4A and B). These compounds were selected as examples of high and low yields among the tested scope of starting materials. The amount of urea was adjusted to 8 equiv. and the observed pressures over the course of the reaction were stable and below the maximum pressure allowed by the set-up (ESI, Fig. S28†). The compounds were purified by simple filtration, drying, and washing with solvents, avoiding column chromatography. Overall, the compounds were obtained with yields comparable or higher than those at 0.6 mmol scale (see ESI, numeral 7†).

**Scheme 5:**
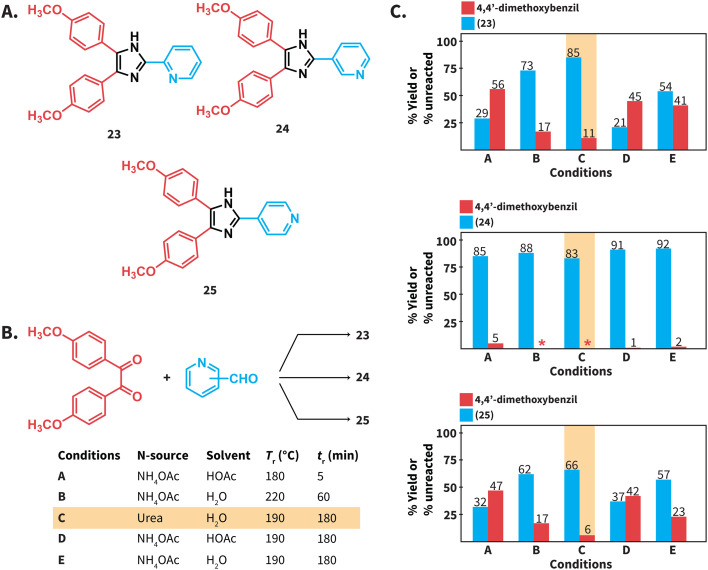
(A) Structures of compounds 23 to 25. (B) Debus–Radzisewski reaction towards 23 to 25 performed under different conditions. (C) Reaction yields (%) for compounds 23 to 25 and unreacted 4,4′-dimethoxybenzil (%) determined *via*^1^H NMR spectroscopy. *The compound was not detected.

**Scheme 6:**
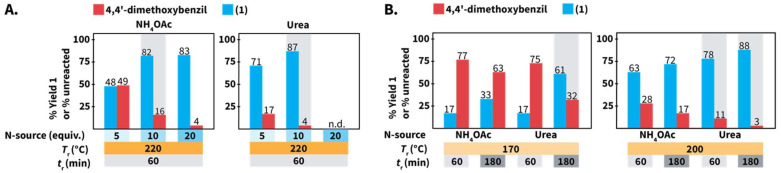
Comparison between NH_4_OAc and urea to synthesize 1 in HTW. (A) Effect of the equivalents’ number. (B) Performance of the model reaction at selected values of *T*_r_ and *t*_r_. Experiments performed using 0.3 mmol of 4,4′-dimethoxybenzil and benzaldehyde, using 1.5 to 6 mmol of N-source, in 3.0 mL of distilled H_2_O. Yields (%) of 1 and amounts (%) of unreacted 4,4′-dimethoxybenzil were calculated *via*^1^H NMR spectroscopy with dimethyl sulfone as internal standard. Gray background indicates that the experiment was performed twice and we show the average values. n.d. = experiment was not performed due to pressure expected to be higher than the maximum value allowed by the set-up.

We next tested the compatibility of our reaction conditions with acid-sensitive starting materials. Established Debus–Radzisewski syntheses are typically performed under acidic conditions owing to the selection of HOAc as solvent and/or the use of NH_4_OAc as N-source that generates HOAc. These syntheses are expected to be incompatible with acid-sensitive starting materials. In this regard, compounds 23 and 25 (Scheme 5A) have been synthesized in DMSO as solvent instead of HOAc owing to the suspected lability of 2-pyridinecarboxaldehyde and 4-pyridinecarboxaldehyde to acidic conditions.^38^ The herein reported synthesis of lophines using urea in HTW avoids the use of NH_4_OAc and HOAc as solvent and we hypothesized that reactions involving acid-sensitive pyridinecarboxaldehydes could work better under our conditions. Therefore, we synthesized compounds 23 to 25*via* (i) a reported MW assisted synthesis using NH_4_OAc in HOAc at 180 °C^39^ (conditions A), (ii) employing NH_4_OAc in H_2_O at 220 °C (conditions B), and (iii) using urea in H_2_O at 190 °C (conditions C) (Scheme 5B). The selected parameters for conditions A correspond to the optimal reported reaction conditions, while conditions B correspond to the optimal parameters for the model reaction using NH_4_OAc (see ESI, Fig. S1†). We found that these syntheses involving HOAc, either as solvent or released from NH_4_OAc, generate 23 and 25 with yields lower than those using urea (Scheme 5C). The combination NH_4_OAc/HTW (conditions B) results in yields of 23 and 25 higher than those using NH_4_OAc/HOAc (conditions A). We hypothesize that the combination NH_4_OAc/HTW exhibits improved yields due to less acidic reaction conditions than those of the combination NH_4_OAc/HOAc. Yet, unreacted 4,4′-dimethoxybenzil is still detected. To our delight, the synthesis of 23 and 25 using urea in HTW resulted in 85% and 66% yield, respectively (Scheme 6C). In contrast to the observations for 23 and 25, the yields of 24 at all tested conditions are comparable. This suggests that the stability of 3-pyridinecarboxaldehyde under acidic conditions is higher than that of the isomers.

We performed the synthesis of compounds 23 to 25 at *T*_r_ = 190 °C for 3 hours in NH_4_OAc/HOAc (conditions D) and NH_4_OAc/H_2_O (conditions E) to directly compare with the *T*_r_ and *t*_r_ presented in this work. The yields of 23 and 25 for reactions performed under conditions D and E were lower than those of reactions using urea/HTW (Scheme 5C). Overall, we propose that the herein presented synthesis of lophines using urea in HTW is suitable for synthesizing lophines that require acid-sensitive starting compounds. Note that the explored examples deal with acid-sensitive aldehydes, whereas 1,2-diketones with acid lability were not tested.

### Comparison between NH_4_OAc and urea to conduct the Debus–Radziszewski MCR in HTW

We further compared the model reaction towards 1 using urea and NH_4_OAc within a broader region of the reaction parameters’ space (Scheme 6). We first studied the role of the equiv. of N-source. At 220 °C, employing the same number of equiv. resulted in better yields of 1 for reactions using urea, *e.g.*, using 5 equiv. resulted in 48% and 71% using NH_4_OAc and urea, respectively (Scheme 6A). Under the assumption of quantitative generation of NH_3_, urea and NH_4_OAc are expected to generate 2 and 1 equiv. of NH_3_, respectively (*cf*Scheme 1A for urea). The yields of reactions using urea are less affected, which might be caused by the generation of NH_3_ in higher amounts when urea is used. Note that the synthesis of 1 using 20 equiv. of urea was not performed since we anticipated that pressure values reaching the limit of the employed set-up at 220 °C would be generated.

In principle, 5 equiv. of urea and 10 equiv. of NH_4_OAc are expected to release similar amounts of NH_3._ The model reaction at 220 °C for 60 min employing 10 equiv. of NH_4_OAc resulted in 82% of 1, while 5 equiv. of urea yielded 71% of 1 (Scheme 6A). We hypothesized that HOAc released from NH_4_OAc exerts a role towards the observed difference. In this respect, the synthesis of quinoxalines in HTW from 1,2-diaryldiketones and *o*-phenylendiamines in HTW using HOAc has been shown to require *t*_r_ shorter than that in solely H_2_O.^23^ Employing 20 equiv. of NH_4_OAc resulted in a yield of 1 (83%) almost identical to that of the reaction using 10 equiv. (82%) (Scheme 6A). This indicated that the yield of 1 reaches a plateau between 10 and 20 equiv. of NH_4_OAc.

The model reaction using NH_4_OAc and urea under different *T*_r_ and *t*_r_ values was also compared (Scheme 6B). After *t*_r_ = 60 min, values of *T*_r_ ≤ 200 °C result in yields lower than those at 220 °C for reactions using both N-sources. Prolonging the *t*_r_ increases the yield of 1 for reactions conducted at *T*_r_ = 200 °C, irrespective of the N-source. Interestingly, 1 was obtained with 17% yield for reactions performed at *T*_r_ = 170 °C after *t*_r_ = 60 min using both N-sources. Yet, the yields are different after *t*_r_ = 180 min (33% using NH_4_OAc and 61% using urea). Overall, decreasing the equiv. of N-source, *T*_r_ or employing shorter *t*_r_ results in drops of yields for 1 using both N-sources. Nonetheless, the yields of reactions using urea were less affected than those using NH_4_OAc under the tested conditions.

### Green chemistry metrics and large-scale comparison with state-of-the-art syntheses

The greenness of the synthesis of lophines in HTW using urea was assessed. We compared the atom economy ((AE = MW of the product/MW of starting materials) × 100) for syntheses using urea and NH_4_OAc. The reaction stoichiometry of the Debus–Radziszewski synthesis establishes that 2 equiv. of NH_3_ are required to synthesize 1 equiv. of lophine. The AE increases from 63% using NH_4_OAc to 68% using urea (calculated for compound 2). This is consistent with the fact that 1 equiv. of urea generates 2 equiv. of NH_3_, while 1 equiv. of NH_4_OAc releases only 1 equiv. of NH_3_.

To further assess the greenness of the synthesis, we next calculated *E*-factor (EF) values (kg waste/kg desired product). We selected this green metric owing to its widespread use in the literature and relatively straightforward calculation. The EF values for the synthesis of selected compounds in HTW using urea range from 18.0 to 253.1 kg kg^−1^. These values include the amount of water and solvents employed for purification (see Table S6, ESI†). We did not consider recovery of solvents. Note that the EF values decrease for compounds that were also synthesized at higher scale, *e.g.*, for compound 1 decreases from 68.9 kg kg^−1^ to 32.9 kg kg^−1^.

Aiming at placing our results within the state-of-the-art, we next conducted a systematic comparison between the herein reported syntheses and reported literature. To this end, we extracted all the reported syntheses towards compounds 1 to 25 from the SciFinder database (details on the data extraction can be found in the ESI†). A total of 401 syntheses reported in literature were found and our work contributes with 23 syntheses. Interestingly, syntheses using 1 equiv. of N-source have been reported. These are considered stoichiometrically unviable and inconsistent with high reaction yields. We suspect that these syntheses correspond to outliers with poorly reported amounts of starting materials. Overall, literature reports are mainly composed of syntheses towards compound 2, which accounts for 311 entries. The reported syntheses were classified in three groups according to the employed reaction media as syntheses performed (i) in H_2_O, (ii) under neat conditions, and (iii) in other reaction media, that include organic solvents or H_2_O/organic solvent mixtures. We found that syntheses performed in H_2_O (4% of syntheses) are scarce, while other reaction media (53%) or neat conditions (43%) are predominant. Clearly, the reported synthesis of lophines in HTW using urea targets the less explored reaction medium and uses the cleanest solvent.

Comparing complete EF values of the syntheses in this work with those of the state-of-the-art is not suitable to achieve a fair assessment of greenness. Quantitative information about purification procedures, *e.g.*, amount of solvent used for recrystallization or column chromatography steps is poorly reported for state-of-the-art syntheses. To compare all the syntheses, we recalculated EF values including the reaction solvents and without work-up procedures (Table 1). The synthesis of compounds 1 to 25 using urea features a range of 16.6 to 156.1 kg kg^−1^. In comparison to state-of-the-art syntheses, this range is only slightly higher than that of reactions performed under neat conditions. Note that the upper limit of the EF range for reactions performed in other media is high and we suspect that this is because the reported reactions were not optimized with respect to concentration of starting materials. We next calculated process mass intensity (PMI = Total mass of material used in a process/mass of product) to complement the assessment of greenness. Under an ideal scenario, the total amount of starting materials is used to synthesize the product and a PMI value close to 1.0 kg kg^−1^ is obtained. The closer the PMI value to 1.0 kg kg^−1^, the greener the process is.

**Table 1:** Ranges for calculated green metrics for the syntheses in this work and state-of-the-art syntheses

	This work	H_2_O	Other media	Neat
EF^a^ (kg kg^−1^)	16.6–156.1	7.2–143.6	0.8–26 107.1	0.3–59.3
PMI^b^ (kg kg^−1^)	17.6–157.1	8.2–144.6	1.8–26 108.1	1.3–60.3
PMI_RRC_^c^ (kg kg^−1^)	2.1–15.2	1.6–5.9	1.1–134.0	1.3–60.3
PMI_S_^d^ (kg kg^−1^)	15.5–141.8	3.7–138.7	0.5–26 103.7	—

a Calculations were performed considering the reaction solvent, no recycling, and expressed as kg of waste/kg of product.

b Calculations were performed considering the reaction solvent and expressed as kg of materials/kg of product.

c kg of starting materials/kg of product.

d kg of solvent/kg of product.

While EF focuses on waste generation, PMI focuses on the reaction inputs and can be further analyzed through the individual contribution of reactants and catalysts (PMI_RRC_), solvents (PMI_s_) and reaction work-up (PMI_w_).^40^ We calculated PMI, PMI_RRC_, and PMI_S_ for all the reactions (Table 1). These metrics rely on typically well reported information, *i.e.*, amount of starting materials, catalyst, and solvent. The range of PMI values for our syntheses span a range comparable to those of reactions performed in water and other media. Reactions performed under neat conditions feature the most favorable range of PMI values, which is justified by the lack of solvent.

**Scheme 7:**
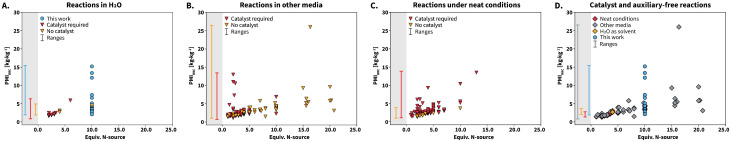
Comparison of PMI_RRC_ values for the synthesis of compounds 1 to 25. Each point represents one synthesis reported either in this work (blue circles) or in the in the SciFinder database (triangles and squares). Reactions were classified according to the reaction media used to conduct the syntheses: (A) in H_2_O, (B) in other media, and (C) under neat conditions. Each plot shows whether the reported synthesis requires catalysts or auxiliar. (D) Catalyst and auxiliary-free reactions were further analyzed according to the employed reaction medium. Ranges of PMI_RRC_ for the compared syntheses are depicted within the gray backgrounds.

Analyzing the individual components of PMI reveals that PMI_s_ is the main contributor to the total PMI value for the syntheses, which is consistent with all state-of-the-art reactions performed in solution. We analyzed the PMI_RRC_ values in more detail and used them to further compare our reactions with the reported literature. This metric has been shown to be more suitable to compare different syntheses where (i) the *c*_M_ is not standarized, (ii) different reactants and catalysts towards several target molecules are used, and (iii) the molecular weight values of the starting materials differ among the compared syntheses.^40^ The calculated PMI_RRC_ values for all the reported syntheses were depicted against equiv. of N-source (Scheme 7). The PMI_RRC_ values of our syntheses range between 2.1–15.2 kg kg^−1^ and are found at 10 equiv. of N-source (Scheme 7A and D, blue circles). The range is mostly comparable to that of reactions in H_2_O and other media, and slightly higher than that of reactions performed under neat conditions (Scheme 7, gray background PMI_RRC_ ranges).

We analyzed the PMI_RRC_ values for each reaction medium according to the use of catalysts or auxiliaries. The usage of these is highly abundant, irrespective of the reaction medium (Scheme 7A to C, red triangles). For syntheses performed in H_2_O (Scheme 7A), 69% require a catalyst or auxiliaries, whereas the percentage grows to 75% and 91% for syntheses conducted in other media (Scheme 7B) and under neat conditions (Scheme 7C), respectively. We observed that syntheses involving catalyst or auxiliaries seem to be more localized in the region between 5 equiv. of N-source or less. This suggests that catalysts influence the PMI_RRC_ values by decreasing the required amount of N-source. However, synthesizing catalysts or auxiliaries inherently requires further starting materials, solvents, and generates waste. The calculated green metrics do not take this into account, and hence we consider that reactions using catalysts would feature less favorable green metrics. Interestingly, the PMI_RRC_ values for the herein presented catalyst-free syntheses match those of state-of-the-art syntheses that require catalyst. We further analyzed only the reported catalyst-free syntheses (Scheme 7D). These are in fact scarce and account for 18% of all the reported syntheses. In particular, among the synthesis under neat conditions, only 9% are catalyst-free. Therefore, while these syntheses feature better green metrics due to the absence of solvent, catalysts are required. Catalyst-free syntheses reported in H_2_O belong to works using NH_4_OAc^30^ and diammonium hydrogenphospate ((NH_4_)_2_HPO_4_).^41^ Overall, urea can be used as non-toxic and highly available N-source in HTW towards lophines, featuring metrics in ranges comparable to those of catalyst-free reported synthesis in H_2_O to-date (Table 1 and Scheme 7A).

### The role of urea in the Debus–Radziszewski MCR towards lophines in HTW

**Scheme 8:**
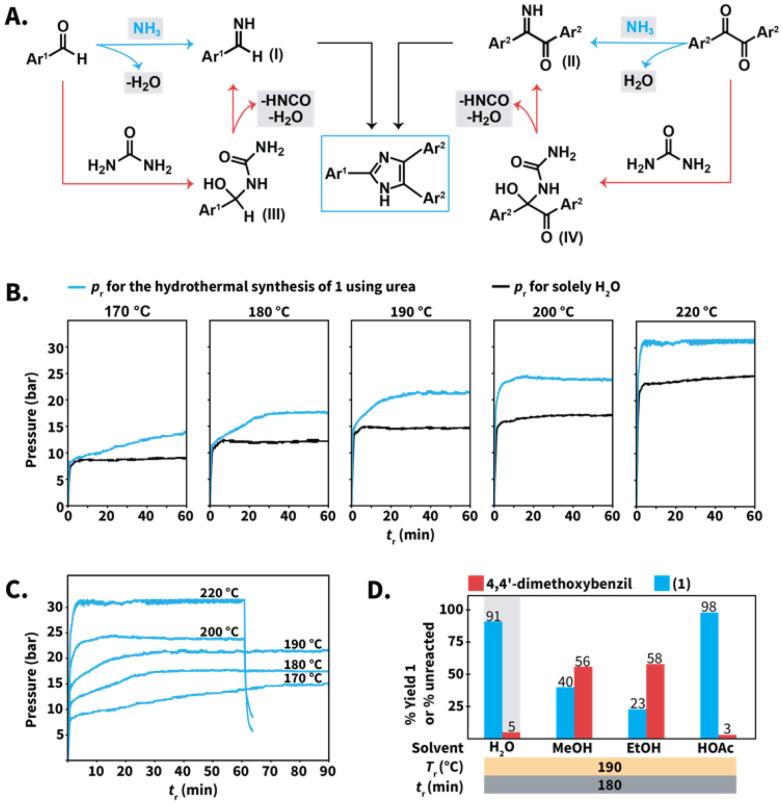
(A) Proposed mechanisms for the formation of lophines using urea in HTW. (B) Profiles of *p*_r_–*t*_r_ for liquid H_2_O (black) and selected reactions towards compound 1 (Scheme 3) using 10 equiv. of urea (blue) at different *T*_r_. (C) Profiles of *p*_r_–*t*_r_ at different *T*_r_ for the synthesis of 1 using urea. (D) Model reaction towards 1 performed in different solvents. Yields (%) of 1 and amounts (%) of unreacted 4,4′-dimethoxybenzil were calculated *via*^1^H NMR spectroscopy with dimethyl sulfone as internal standard. Gray background indicates that the experiment was performed twice and we show the average yield or unreacted amount.

Finally, we gained insight into the role of urea to conduct the Debus–Radziszewski synthesis in HTW. We hypothesized that the reaction was mainly controlled by the aquathermolysis of urea. This reaction generates NH_3_ and CO_2_, which are distributed in both the liquid and the gas phase as a function of different factors, *e.g.*, their solubility in HTW. The produced NH_3_ reacts with 1,2-diketones and aldehydes towards imines, *e.g.*, benzylimines (Scheme 8A, structure I) and α-iminoketones (Scheme 8A, structure II) that form the imidazole ring (Scheme 8A, blue pathway). This proposal is based on theoretical and experimental studies of the formation of simple imidazoles, pointing at imine intermediates.^42^ On the other side, direct reaction between urea and the employed 1,2-diketones or aldehydes towards addition intermediates III and IV is conceivable (Scheme 8A, orange pathway). This pathway is based on the proposed mechanism for, *e.g.*, the Biginelli MCR, that starts with the nucleophilic addition of urea to aryl aldehydes.^43,44^ The intermediates III and IV would undergo cleavage to generate the corresponding α-iminoketones I and benzylimines II.

Both mechanisms might in principle take place simultaneously. Nevertheless, drops in reaction yields for the model reaction towards compound 1 were observed after decreasing from 10 to 5 equiv. of urea at selected *T*_r_ values (Scheme 3B). The employed excess of urea is expected to be sufficient to form addition products yet inherently generates lower amounts of NH_3_. This suggests that the aquathermolysis of urea plays a major role in propelling the imidazole formation. The proposed routes involve the generation of NH_3_, HNCO, and H_2_O, which are expected to increase the pressure at the employed *T*_r_ values. Thus, we conjectured that further insight could be gained by analyzing the reaction pressure (*p*_r_) over the course of the syntheses.

For the synthesis of compound 1 using urea (Scheme 3), the maximum observed *p*_r_ values ranged between 15 bar (at 170 °C) to 30–31 bar (at 220 °C) (Scheme 8B, blue curves and ESI for all values†). More importantly, the observed *p*_r_ values are higher than those generated by solely H_2_O under comparable conditions (Scheme 8B, black curves). The difference in *p* is justified by the presence of gaseous species, presumably HNCO, NH_3_ and CO_2_. If formed, HNCO would rapidly undergo hydrolysis to NH_3_ and CO_2_. For better estimating the order of magnitude of the total *p* under the employed conditions, we calculated the values of *p* using the van der Waals equation for non-ideal gas behavior. Assuming complete formation of NH_3_ and CO_2_ as gases produced from urea, values of *p*_r_ ranging between 54 bar (at 180 °C) and 59 bar (at 220 °C) were calculated (see ESI for calculation†). This range is higher than the range observed *p*_r_. The differences are however expected as (i) we did not consider the solubility of NH_3_ and CO_2_ in liquid HTW, and (ii) NH_3_ is partially consumed by the imidazole formation. In summary, the *p*_r_–*t*_r_ profiles using urea at different *T*_r_ values are consistent with the generation of gases, presumably NH_3_ and CO_2_. At values of 170 °C ≤ *T*_r_ ≤ 220 °C, the maximum *p*_r_ decreases as a function of the *T*_r_ (Scheme 8B). This is expected, since (i) H_2_O's autogenous pressure decreases as a function of *T*_r_ and (ii) the amount and partial pressure of NH_3_ and CO_2_ is also expected to be a function of the *T*_r_.

More interestingly, the *p*_r_–*t*_r_ profiles revealed that the maximum *p*_r_ is reached after different *t*_r_ (Scheme 8C). At 220 °C, the maximum observed *p*_r_ is reached within 2 min and matches the maximum operating *p* of the employed MW reactor, *i.e.*, 30 to 31 bar. After this, the *p*_r_ did not further increase. Therefore, the *p*_r_–*t*_r_ profile at 220 °C indicates that gases are readily produced during the first minutes of reaction and rapidly contribute to reach the maximum *p*_r_. For experiments at *T*_r_ ≤ 200 °C, the maximum *p*_r_ is reached after values of *t*_r_ longer than 2 min (Scheme 8C). At 200 °C, the maximum *p*_r_ was reached after 10 min, while at least 15 min and 30 min are required at 190 °C and 180 °C, respectively. At 170 °C, the maximum *p*_r_ was observed after 60 min. Therefore, the observed *p*_r_–*t*_r_ profiles indicate that the *T*_r_ can be used to tune the release of NH_3_. The lower the *T*_r_, the longer the *t*_r_ required to reach the maximum *p*_r_. Among the tested *T*_r_ values, 170 °C exhibits an increase in pressure – and as we believe consequently NH_3_ release – that extends over the longest time span.

To gain a deeper understanding of the urea's role for synthesizing compound 1, we performed the model reaction in different organic solvents. We selected ethanol (EtOH), methanol (MeOH), and HOAc (Scheme 8D). These solvents are the most frequently employed in reported NH_4_OAc-based Debus–Radziszewski syntheses (*cf.*Scheme 1D). The alcoholysis of urea has received attention to synthesize organic carbonates. In particular, EtOH and MeOH react with urea towards organic carbonates with NH_3_ produced as byproduct.^45^ These reactions generally rely on metallic catalysts although supercritical MeOH has been reported to react with urea (265 °C for 2 h) towards dimethylcarbonate.^46^ Therefore, we tested whether the compatibility of the selected solvents with the thermolysis of urea could be different from that of HTW, influencing the outcome of the model reaction towards 1 to some extent.

While the synthesis of 1 in HTW yielded solid products after completion, the reactions performed in the selected solvents yielded solutions and the crude products were isolated after pouring the reaction into water, neutralization, and filtration (see ESI†). The reaction performed in HOAc produced 1 with 98% yield (NMR yield), suggesting that the acidity provided by HOAc contributes to the role of urea as N-source. In stark contrast, the reactions performed in EtOH and MeOH after 180 min produced 1 with 23% and 40% yield, respectively. We propose that the low yields are a consequence of the insufficient generation of NH_3_ from urea, supporting the synergy between the pair urea/HTW to propel the imidazole synthesis.

## Conclusions

In sum, we show that the Debus–Radziszewski MCR in HTW using alternative N-sources can be performed in laboratory MW set-ups and does not require additional catalysts or promotors. In particular, HTW can be used as green reaction medium to unlock the use of urea as N-source to synthesize lophines under different combinations of *T*_r_ and *t*_r_. The presented reaction parameters were compatible with a broad scope of starting materials. Particularly, lophines synthesized from acid-labile pyridine carbaldehydes were obtained with yields comparable to or higher than those of synthetic approaches involving NH_4_OAc. We observed low performance for halogenated 1,2-diaryldiketones and furane-bearing 2-diaryldiketones. The syntheses using urea/HTW exhibits *E*-factor and PMI_RRC_ values comparable to those of state-of-the-art syntheses, uses water, a clean and non-toxic solvent, and does not require catalysts or reaction auxiliaries. We propose that the generation of NH_3_*via* urea aquathermolysis is propelling the formation of lophines, since experiments using urea in superheated alcohols resulted in poor reaction yields. The herein presented methodology is highly accessible from a practical perspective. Overall, our contribution showcases the power of HTW as reaction medium in the synthesis of heteroaromatic compounds.

## Author contributions

Fabian Amaya Garcia: project design, synthesis, data acquisition and analysis, co-supervision, green metrics calculations and assessment of sustainability, manuscript writing. Lena Schittenhelm: synthesis. Miriam M. Unterlass: project design, supervision, and manuscript writing.

## Data availability

In accordance with the principles of transparency and reproducibility, we have included the data any data required to understand and verify the research in this article as part of the main paper and in the ESI of this article.† Researchers can access and utilize this data.

## Conflicts of interest

There are no conflicts to declare.

## Supplementary Material

GC-026-D4GC01705F-s001
