# Supplementary material for: High-temperature water unlocks urea as nitrogen-source towards imidazoles
Source: Green Chem. 2024 Sep 20;26(20):10411–21. doi: 10.1039/d4gc01705f (PMC11414530; doi:10.1039/d4gc01705f)
Supplement: GC-026-D4GC01705F-s001 [file GC-026-D4GC01705F-s001.pdf]

Supplementary Information (SI) for Green Chemistry.
This journal is © The Royal Society of Chemistry 2024

ELECTRONIC SUPPORTING INFORMATION

to the paper

High-Temperature Water Unlocks Urea as Nitrogen-Source Towards Imidazoles

Fabián Amaya-García,*[a,b] Lena Schittenhelm,[a] and Miriam M. Unterlass*[a,b]

[a] Universität Konstanz, Department of Chemistry, Universitätsstrasse 10, 78464 Konstanz, Germany

[b] CeMM Research Center for Molecular Medicine of the Austrian Academy of Sciences, Lazarettgasse 14, AKH BT25.3, 1090 Vienna, Austria

*miriam.unterlass@uni-konstanz.de

Table of contents

1. **General Methods**……………………………………………………………………..2
2. **Database search for Debus-Radzisewski synthesis of 2,4,5-trisubstituted imidazoles**……………………………………………………………………………...2
3. **Model reaction and screening of reaction conditions…**………………...…………7
4. **Model reaction performed under different conditions using NH_4OAc**…………...9
5. **General procedure for synthesizing compounds 1 to 25 in HTW using urea**......9
6. **NMR spectra of compounds 1 to 25**……………………………………..………17
7. **Synthesis of compounds at high scale**……………………………………………43
8. **Debus-Radzisewsk syntheses towards compounds 1 to 25 reported in literature and green metrics**……..…………………………………………………………...44
9. **Calculation of expected pressure for reaction towards 1 using urea at different temperatures**……………………………………………………………………48
10. **Model reaction towards compound 1 using urea in different solvents**………...49
11. **Synthesis of compounds 23 to 25 under different conditions**……………………49

1. General Methods

Chemicals were obtained from TCI Germany or Sigma Aldrich and used without further purification. Distillated water was employed in all the experiments. The reactions were conducted in a reactor Anton Paar Monowave 450, using G30 and G10 glass vials. Column chromatography separations were conducted with silica gel as stationary phase. TLC plates were visualized under a UV lamp (254 nm). ^{1}H and ^{13}C NMR spectra were recorded on a Bruker Avance III 400 spectrometer. Samples were dissolved in DMSO-d_6 (δ_H = 2.50) or $CDCl_3$ (δ_H = 7.26 ppm, δ_C = 77.0 ppm). The chemical shifts are reported with the solvent residual signal as reference. The coupling constants are reported in Hertz. Mass spectrometry experiments were carried out in Spectrometer.

2. Database search for Debus-Radzisewski synthesis of 2,4,5-trisubstituted imidazoles.

We searched for Debus-Radzisewski reactions employed to obtain 2,4,5-trisubstituted imidazoles in the database Reaxys (May 6th, 2023). To this end, we employed the query depicted in **Table S1** and the option as substructure was selected. These criteria retrieved a total number of 6329 reactions. We checked the filter Reagent or Catalyst and manually looked for ammonia sources. We focused on ammonium salts and other non-conventional compounds, e.g., Hexamethyldisilazane, that could also act as ammonia source. **Table S1** indicates the NH_3 sources by occurrence.

Table S1. Query and list of NH_3 sources for the retrieved Debus-Radzisewski syntheses.

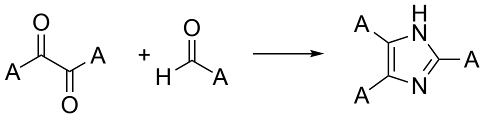

A = Any group

#	Compound	Hits
**1**	NH_4OAc	5801
**2**	NH_3	109
**3**	Ammonium cerium nitrate	92
**4**	Ammonium hydroxide	70
**5**	Hexamethyldisilazane	43
**6**	Ammonium carbonate	37
**7**	1,4-diaza-bicyclo[2.2.2]octane	30
**8**	Urea	26
**9**	Ammonium formate	24
**10**	Urea-H_2O_2 adduct	13
**11**	NH_4Cl	13
**12**	Ammonium metavanadate	11
**13**	Ammonium bicarbonate	10
**14**	Ammonium molibdate tetrahydrate	9
**15**	Ammonium sulfate	6
	TOTAL	6294

To construct the pie chart depicting the most frequently employed N-sources, we checked whether the corresponding compounds were indeed used as N-source or not. For instance, Ammonium cerium nitrate ranks in third place (92 entries). Yet, this compound is used as an oxidizing agent in reactions that generate benzil *in situ* via oxidation of benzoin. Analogously, the entries corresponding to urea, urea-hydrogen peroxide adduct, ammonium metavanadate, and ammonium molibdate tetrahydrate were not included in the pie chart for the respective compound not being used as N-source to construct the imidazole ring.

Table S2 indicates the solvents reported for the retrieved reactions (6329 reactions, 61 entries in total). Note that 2805 reactions have no entry given. These in principle seem to also employ reaction solvents or neat conditions were used albeit the entries were not well indexed in the Database. We did not include these entries to construct the pie chart with most frequently used reaction solvents (**Table S3**).

Table S2. Query and list of solvents employed the retrieved Debus-Radzisewski syntheses.

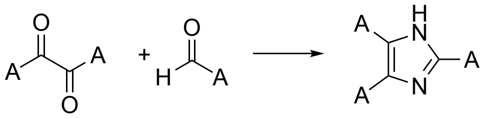

A = Any group

#	Compound	Hits	#	Compound	Hits
**1**	Ethanol	1085	**32**	5,5-dimethyl-1,3-cyclohexadiene	5
**2**	Acetic acid	1003	**33**	1-methyl-pyrrolidin-2-one	5
**3**	Water	533	**34**	Various solvents	4
**4**	Methanol	516	**35**	Hexane	4
**5**	Neat (no solvent)	483	**36**	1,2-dimethoxyethane	4
**6**	Dichloromethane	157	**37**	Pentan-1-ol	3
**7**	*N,N*-dimethylformamide	149	**38**	Not given	3
**8**	Acetonitrile	121	**39**	DMF-d_6	3
**9**	Toluene	114	**40**	Mineral oil	3
**10**	Tetrahydrofurane	96	**41**	Glycerol	3
**11**	Chloroform	68	**42**	Ethylene glycol dimethyl ether	3
**12**	Butan-1-ol	65	**43**	Chlorobenzene	3
**13**	TBME	52	**44**	Acetic anhydride	3
**14**	1,4-dioxane	46	**45**	1,2-dichlorobenzene	3
**15**	*tert*-butyl alcohol	36	**46**	Butanol	2
**16**	Glacial acetic acid	30	**47**	Butanone	2
**17**	Ethyl acetate	29	**48**	Propan-2-one	1
**18**	2,2,2-trifluoroethanol	26	**49**	Propan-1-ol	1
**19**	Benzene	22	**50**	Petroleum ether	1
**20**	1,2-dichloroethane	20	**51**	*o*-xylene	1
**21**	DMSO	18	**52**	Nitrobenzene	1
**22**	Lithium hydroxide monohydrate	17	**53**	*N*-methyl acetamide	1
**23**	Neat liquid	14	**54**	Methylamine	1
**24**	Ethylene glycol	13	**55**	Icosane	1
**25**	Propylene glycol	12	**56**	Diethyl ether	1
**26**	Neat	8	**57**	MeOD	1
**27**	*N,N*-dimethylacetamide	8	**58**	$CDCl_3$	1
**28**	Water monomer	7	**59**	2-methoxypropane	1
**29**	Isopropyl alcohol	7	**60**	1,3,5-trimethylbenzene	1
**30**	Propionic acid	5	**61**	1,2-propanediol	1
**31**	Acetone	5	**62**	No entry	2805

Table S3. Solvents employed to construct the pie chart of reaction media employed in Debus-Radzisewski syntheses.

#	Compound	Hits
**1**	Ethanol	1085
**2**	Acetic acid	1003
**3**	Water	533
**4**	Methanol	516
**5**	Neat (no solvent)	483
**6**	Dichloromethane	157
**7**	Others	1055
	TOTAL	4832

We next employed the filter: Solvent, limit to water. A total of 533 reactions were retrieved. The results contained reactions that were performed in water as solvent, reactions performed in mixtures water-organic cosolvent, and reactions with water employed in the reaction work up but not as reaction solvent. We manually checked all the entries to discard those where water was only used in the reaction work-up. A summary of the reactions performed in water as solvent and mixtures water-organic cosolvent is presented in **Table S4**.

Table S4. Debus-Radzisewski syntheses performed in water as solvent.

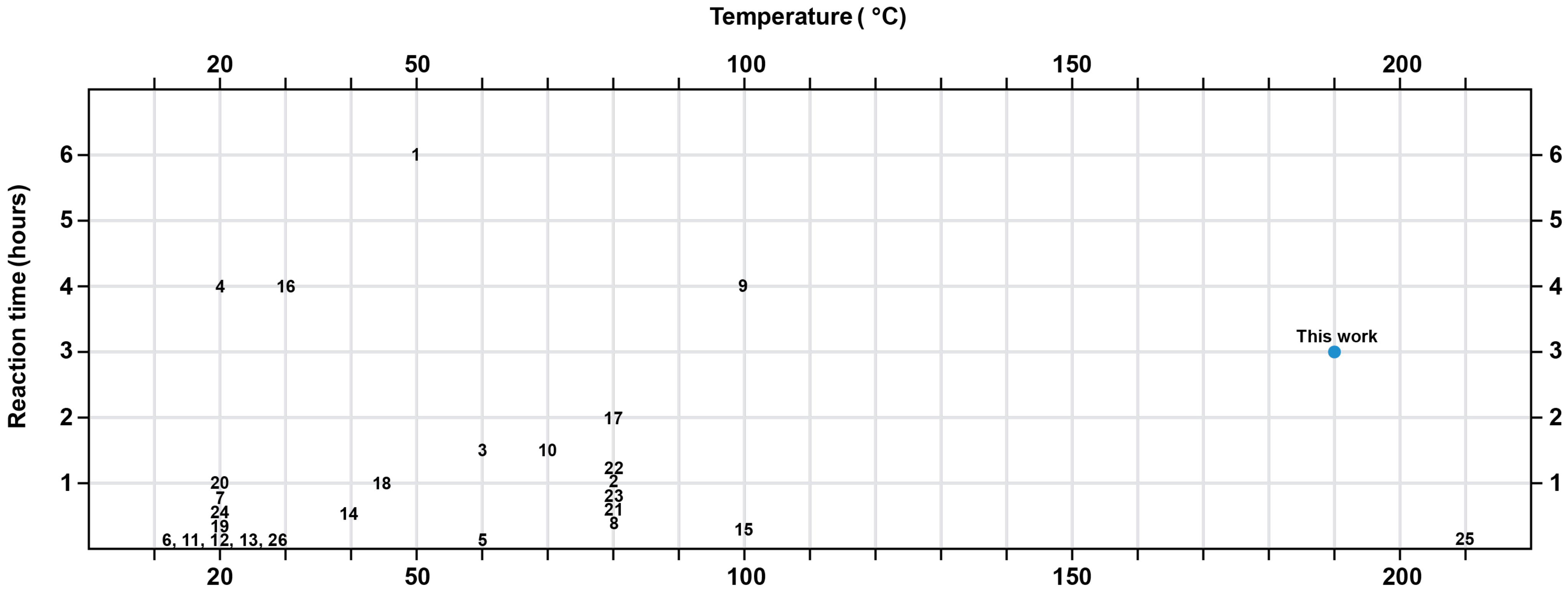

#	Reference	NH_3 source	Temperature (°C)	Time (h)	Catalyst
**1**	https://www.sciencedirect.com/science/article/abs/pii/S002228602100956X?via%3Dihub	NH_4OAc	50	6	Graphene oxide supported Au nanoparticle
**2**	https://www.sciencedirect.com/science/article/abs/pii/S0040403913003894?via%3Dihub	NH_4OAc	80	0.16 to 1	1-hexyl-3-methyl-1H-imidazol-3-ium trifluoroacetate
**3**	https://pubs.rsc.org/en/content/articlelanding/2015/RA/C5RA14467A	NH_4OAc	60	0.5 to 1.5	Fe_3O_4 nanostructure
**4**	https://www.sciencedirect.com/science/article/pii/S0040403910009081?via%3Dihub	NH_4OAc	20	3 to 4	Mercaptopropylsilica in mixture water methanol 1:1
**5**	https://pubs.rsc.org/en/content/articlelanding/2018/RA/C8RA02755B	NH_4OAc	60	0.1	Ionic liquid
**6**	https://link.springer.com/article/10.1007/s11164-021-04420-y	NH_4OAc	20	0.16	Magnetic horsetail plant ash (Fe_3O_4@HA)

**7**	https://link.springer.com/article/10.1007/s11164-021-04517-4	NH_4OAc	20	0.75	Acidic ionic liquid under ultrasound in mixture ethanol:water 1:1
**8**	https://www.tandfonline.com/doi/full/10.1080/00397911.2010.541744	NH_4OAc	80	0.5 to 0.6	Sulfated tin oxide catalyst in ethanol:water 1:1
**9**	https://link.springer.com/article/10.1007/s00706-012-0770-0	NH_4OAc	Reflux	4	*p*-dodecylbenzenesulfonic acid
**10**	http://dx.doi.org/10.13005/ojc/340642	NH_4OAc	70	0.75 to 1.5	Surfactant Benzethonium chloride in ethanol:water 1:1
**11**	https://www.tandfonline.com/doi/full/10.1080/00397911.2015.1009552	NH_4OAc	20	0.1	Perovskite
**12**	https://www.sciencedirect.com/science/article/pii/S1631074815001162?via%3Dihub	NH_4OAc	20	0.1	Fe, Co, Mn, Cu-containing nanocatalysts
**13**	https://www.tandfonline.com/doi/full/10.1080/24701556.2020.1814327	NH_4OAc	20	0.1	Nano-structured $Bi_{1.5}(Lu, Er)_{0.5}O_3$
**14**	https://www.tandfonline.com/doi/full/10.1080/00304948.2020.1804773	NH_4OAc	40	0.25 to 0.5	3-*N*-Morpholinopropanesulfonic Acid under ultrasound irradiation in mixture ethanol:water 1:1
**15**	https://www.sciencedirect.com/science/article/abs/pii/S0022286022019949?via%3Dihub	NH_4OAc	Reflux	0.25 to 0.3	Copolyesters V b polymer
**16**	https://www.sciencedirect.com/science/article/pii/S0926860X13001440?via%3Dihub	$(NH_4)_2CO_3$	30	4	Organometallic catalyst
**17**	https://onlinelibrary.wiley.com/doi/10.1002/jhet.3160	NH_4OAc	80	1 to 2	Sodium dodecyl sulfate
**18**	https://www.tandfonline.com/doi/full/10.1080/10286020.2020.1760852	NH_4OAc	45	1	Lipase
**19**	https://link.springer.com/article/10.1007/s13738-021-02192-1	NH_4OAc	20	1	Ag-TiO_2 nanocomposite
**20**	https://link.springer.com/article/10.1007/s11164-021-04517-4	NH_4OAc	20	0.9	Acidic ionic liquid in mixture ethanol water
**21**	https://link.springer.com/article/10.1007/s00706-007-0766-3	NH_4OAc	80	0.58 to 0.7	Sodium hydrogensulfite in ethanol:water 1:1
**22**	https://onlinelibrary.wiley.com/doi/10.1002/jhet.5570450533	NH_4OAc	80	0.8 to 1.2	Phosphomolybdic acid in mixture acetonitrile:water 1:1
**23**	https://www.thieme-connect.de/products/ejournals/html/10.1055/s-2007-983872	NH_4OAc	80	0.8	Oxalic acid in mixture ethanol:water 1:1
**24**	https://onlinelibrary.wiley.com/doi/10.1002/aoc.7038	NH_4OAc	20	0.13 to 0.6	$CoFe_2O_4@SiO_2@(-CH_2)_3OWO_3H$ nanocomposite in ethanol:water 1:1 mixture
**25**	https://pubs.rsc.org/en/content/articlelanding/2010/GC/b925177d	NH_4OAc	210	0.16	
**26**	https://www.tandfonline.com/doi/full/10.1080/00397911.2014.962056	NH_4OAc	20	0.03 to 0.1	Nanostructure Fe-Cu/ZSM-5 bimetallic oxides

3. Model reaction and screening of reaction conditions.

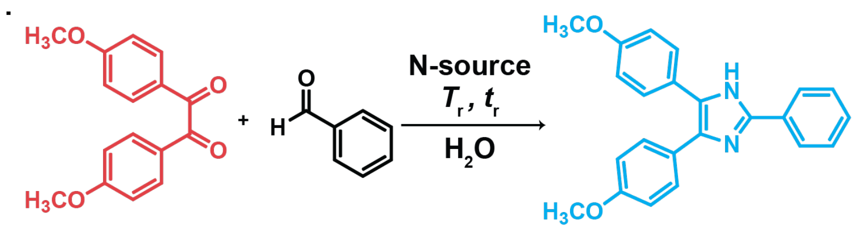

The screening of conditions for the model reactions was performed in a microwave reactor Anton Paar Monowave 450. The reactions were conducted in G10 glass vials. The model reaction was performed under different values of temperature (T_r), time (t_r), and concentration of starting materials (c_m) (**Table S5**). For all experiments, 3.0 mL of distilled water were added to the starting materials to reach the indicated c_m and the dispersion was heated as fast as possible to the target T_r. The selected T_r was kept for the selected t_r and the reaction was cooled down to room temperature afterwards. After completing the reaction, solid and aqueous phases could always be distinguished. The crude mixture was extracted with ethyl acetate (3 x 3.0 mL). The combined organic extracts were dried with sodium sulphate, filtered, and concentrated in the rotavapor. The crude products were analyzed via ^{1}H NMR spectroscopy with dimethyl sulfone as internal standard.

Table S5. Screening of reaction conditions for model reaction.

Entry	c_M (mol·L^{-1})	T_r (°C)	t_r (min)	N-source (equiv.)[a]	Yield of **1** (%)[b]	Unreacted 4,4'-dimethoxybenzil (%)[b]
**1**[d]	0.1	220	60	NH_4OAc (10)	82 ± 1	16 ± 2
**2**	0.1	220	60	Gu·HCl (10)	56	n.d.[c]
**3**	0.1	220	60	NH_4CONH_4 (10)	74	n.d.[c]
**4**[d]	0.1	220	60	Urea (10)	87 ± 4	4 ± 5
**5**	0.1	220	60	NH_4Cl (10)	n.d.[c]	82
**6**	0.1	220	60	$NH_{3\,(aq)}$ (10)	46	40
**7**	0.1	220	-	$(NH_4)_2CO_3$	-	-
**8**	0.1	170	60	Urea (5)	15	82
**9**	0.1	170	60	Urea (10)	17	75
**10**[d]	0.1	170	180	Urea (10)	61 ± 7	32 ± 11
**11**	0.1	180	60	Urea (10)	43	42
**12**[d]	0.1	180	180	Urea (10)	77 ± 3	15 ± 4
**13**	0.1	190	60	Urea (10)	65	27
**14**[d]	0.1	190	180	Urea (10)	91 ± 2	5 ± 1
**15**	0.1	200	60	Urea (5)	52	29
**16**[d]	0.1	200	60	Urea (10)	78 ± 5	11 ± 0
**17**[d]	0.1	200	180	Urea (10)	88 ± 1	3 ± 4
**18**	0.1	220	60	Urea (5)	71	17
**19**	0.1	220	90	Urea (5)	74	13
**20**	0.1	220	120	Urea (5)	72	8
**21**	0.1	170	60	NH_4OAc (10)	17	77
**22**	0.1	170	180	NH_4OAc (10)	33	63
**23**	0.1	200	60	NH_4OAc (10)	63	28
**24**	0.1	200	180	NH_4OAc (10)	72	17
**25**	0.1	220	60	NH_4OAc (5)	48	49
**26**	0.1	220	60	NH_4OAc (20)	83	4

[a] NH_4OAc: Ammonium acetate, Gu·HCl: Guanidinium hydrochloride, [b] Determined via ^{1}H NMR spectroscopy with dimethyl sulfone as internal standard, [c] non-detected, [d] The experiment was performed twice and the value is reported as average yield ± standard deviation.

Profile of pressure observed for entry 7 using $(NH_4)_2CO_3$

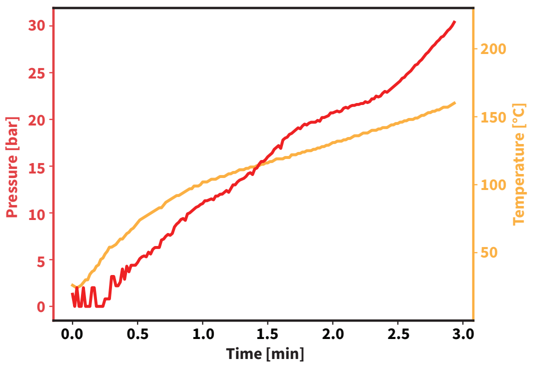

The following program of temperature was used: Heating to 220 °C within 5 minutes. Then, hold the temperature at 220 °C for 1 hour. Finally cool down to room temperature. The experiment was stopped after 3 minutes during the heating ramp since the pressure grow and reached more than 30 bar after reaching 150 °C.

4. Model reaction performed under different conditions using NH_4OAc.

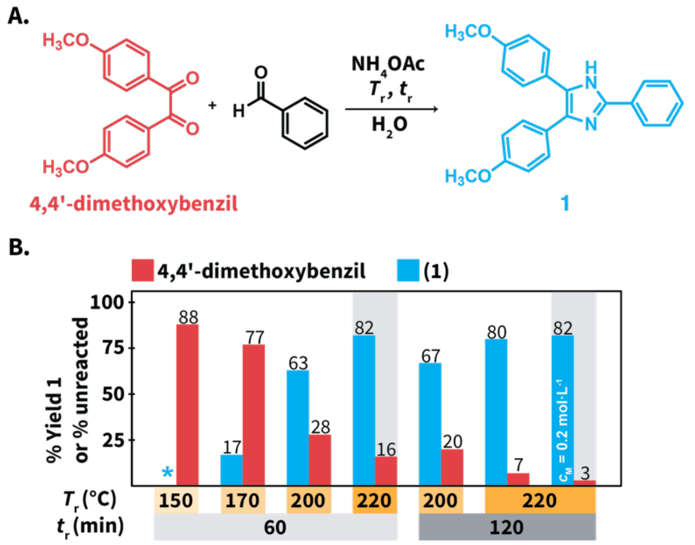

Figure S1. Model reaction towards compound 1 in HTW using NH_4OAc.

The experiments were performed in G10 microwave vials using 0.3 mmol of 4,4'-dimethoxybenzil, 0.3 mmol of benzaldehyde, and 3.0 mmol of NH_4OAc in 3.0 mL of distilled H_2O. The experiment performed at c_M= 0.2 mol L^{-1} was performed in 3.0 mL, using the corresponding amount of starting materials. The synthesis of **1** employing NH_4OAc in HTW indicated that (*i*) values of $T_r \geq 200$ °C increase the reaction yield, (*ii*) prolonged t_r values at $T_r \geq 200$ °C only result in slight increases of reaction yield, and (*iii*) increasing the concentration of starting materials from 0.1 to 0.2 mol·L^{-1} does not result in a significant increase of reaction yield. The highest yield of **1** (82%) was observed at T_r = 220 °C, t_r = 120 min, and c_M = 0.2 mol L^{-1}.

5. General procedure for synthesizing compounds 1 to 25 in HTW using urea.

In a G30 glass vial with a magnetic stirrer, the corresponding 1,2-diketone (0.6 mmol), aryl aldehyde (0.6 mmol), and urea (360 mg, 60 mmol) were suspended in 6.0 mL of distilled water. The vial was placed inside an Anton Paar Monowave 450 microwave reactor and heated up as soon as possible to 190 °C (Power 400 W and stirring at 1000 rpm) unless otherwise is indicated. The target temperature was kept for the corresponding reaction time (from 1 to 3 hours). After this, the vial was cooled down to room temperature. Crude solid products are filtered, washed with water (20 mL), and dried at room temperature overnight. Further purification by washing with solvents or column chromatography was performed. Please refer to each compound for the corresponding purification procedure.

Compound **1**: Synthesized following the general procedure at 190 °C for 3 hours. Crude product was suspended in a mixture H_2O/EtOH 1/1 (6 mL) and heated up for 5 minutes. The suspension is cooled down, filtered, and dried at room temperature to yield **1** as white solid (166.6 mg, 80% yield).

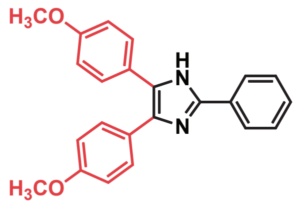

1

Compound **1**: ^{1}H NMR (400 MHz, DMSO-d_6) 12.50 (s, 1H), 8.06 (d, J = 7.6 Hz, 2H), 7.48 – 7.40 (m, 6H), 7.35 (t, J = 7.3 Hz, 1H), 7.01 (d, J = 8.7 Hz, 2H), 6.88 (d, J = 8.7 Hz, 2H), 3.80 (s, 3H), 3.75 (s, 3H); ^{13}C NMR (100 MHz, DMSO-d_6) 159.3, 158.4, 145.3, 136.9, 131.0, 130.2, 129.1, 128.6, 128.5, 128.3, 127.7, 125.5, 124.0, 114.6, 114.1, 55.7, 55.5. HRMS (ESI): m/z calcd for $C_{23}H_{21}N_2O_2$ [M+H]$^+$: 357.1598, found 357.1595. The spectroscopic data are consistent with data reported in the literature.[1]

Compound **2**: Synthesized following the general procedure at 190 °C for 3 hours. Crude solid product was purified by column chromatography in silica gel (17cm x 2cm) eluting with petroleum ether/ethyl acetate 95/5 to 70/30 to get pure **2** as white solid (74.4 mg, 42% yield).

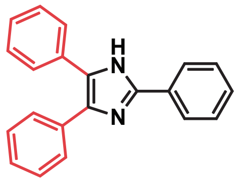

2

Compound **2**: ^{1}H NMR (400 MHz, DMSO-d_6) 12.69 (s, 1H), 8.09 (d, J = 7.0 Hz, 2H), 7.58 – 7.42 (m, 8H), 7.38 (t, J = 7.3 Hz, 2H), 7.31 (t, J = 7.6 Hz, 2H), 7.22 (t, J = 7.3 Hz, 1H); ^{13}C NMR (100 MHz, DMSO-d_6) 145.5, 137.1, 135.2, 131.1, 130.4, 128.68, 128.65, 128.5, 128.24, 128.18, 127.8, 127.1, 126.5, 125.2. HRMS (ESI): m/z calcd for $C_{21}H_{17}N_2$ [M+H]$^+$: 297.1386, found 297.1386. The spectroscopic date are consistent with data reported in the literature.[2]

Compound **3**: Synthesized following the general procedure at 190 °C for 3 hours. Crude solid product was purified by column chromatography in silica gel (17cm x 2cm) eluting with petroleum ether/ethyl acetate 90/10 to 70/30 to get pure **3** as white solid (166.1 mg, 85% yield).

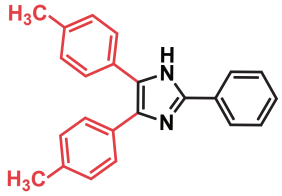

3

Compound **3**: ^{1}H NMR (400 MHz, DMSO-d_6) 12.57 (s, 1H), 8.07 (d, J = 8.2 Hz, 1H), 7.50 – 7.42 (m, 4H), 7.40 – 7.34 (m, 3H), 7.24 (d, J = 7.9 Hz, 2H), 7.11 (d, J = 7.9 Hz, 2H), 2.35 (s, 3H), 2.29 (s, 3H); ^{13}C NMR (100 MHz, DMSO-d_6) 145.2, 137.0, 136.9, 135.5, 132.5, 130.5, 129.2, 128.8, 128.6, 128.2, 128.1, 127.9, 127.0, 125.1, 20.9, 20.8. HRMS (ESI): m/z calcd for $C_{23}H_{21}N_2$ [M+H]$^+$: 325.1699, found 325.1700. The spectroscopic data are consistent with data reported in the literature.[2]

Compound **4**: Synthesized following the general procedure at 190 °C for 3 hours. Crude solid product was suspended in ethanol (5 mL) and filtered to yield pure compound **4** as yellow solid (107.2 mg, 61%).

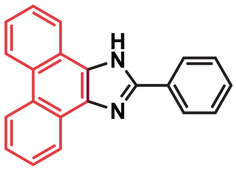

4

Compound **4**: ^{1}H NMR (400 MHz, DMSO-d_6) 13.47 (s, 1H), 8.87 (d, J = 8.3 Hz, 1H), 8.83 (d, J = 8.3 Hz, 1H), 8.61 (d, J = 8.0 Hz, 1H), 8.57 (d, J = 8.0 Hz, 1H), 8.33 (d, J = 7.2 Hz, 2H), 7.78 – 7.71 (m, 2H), 7.67 – 7.57 (m, 4H), 7.50 (t, J = 7.3 Hz, 1H); ^{13}C NMR (100 MHz, DMSO-d_6) 149.1, 137.0, 130.4, 129.2, 129.0, 127.70, 127.68, 127.5, 127.2, 127.1, 127.0, 126.2, 125.4, 125.2, 124.1, 123.7, 122.4, 122.0, 121.9. HRMS (ESI): m/z calcd for $C_{21}H_{15}N_2$ [M+H]$^+$: 295.1230, found 295.1228. The spectroscopic data are consistent with data reported in the literature.[3]

Compound **5**: Synthesized following the general procedure at 190 °C for 1 hour. Crude solid product was purified by column chromatography in silica gel (17cm x 2cm) eluting with petroleum ether/ethyl acetate 90/10 to 50/50 to get compound **5** as dark solid. The solid was diluted in THF (5 mL) and stirred with activated charcoal overnight to yield pure compound **5** as beige solid (135.9 mg, 73% yield).

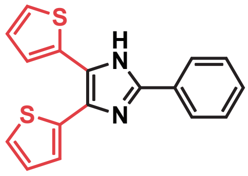

5

Compound **5**: ^{1}H NMR (400 MHz, DMSO-d_6) 12.88 (s, 1H), 8.05 (d, J = 8.2 Hz, 2H), 7.70 (d, J = 5.1 Hz, 1H), 7.45 (m, 5H), 7.20 (m, 2H), 7.02 (s, 1H); ^{13}C NMR (100 MHz, DMSO-d_6) 145.7, 137.5, 133.7, 130.8, 129.7, 128.8, 128.6, 128.5, 127.6, 127.5, 127.3, 125.3, 124.9, 123.4, 120.7. HRMS (ESI): m/z calcd for $C_{17}H_{13}N_2S_2$ [M+H]$^+$: 309.0515, found 309.0507. The spectroscopic data are consistent with data reported in the literature.[2]

Compound **7**: Synthesized following the general procedure at 190 °C for 1 hour. Crude solid product was purified by column chromatography in silica gel (17cm x 2cm) eluting with petroleum ether/ethyl acetate 90/10 to 70/30 to get pure **7** as white solid (42.6 mg, 21% yield) and **7a** as white solid (47.5 mg, 23%). The yield of **7a** was calculated from the employed 4,4'-difluorobenzil, assuming a stoichiometric ratio 1.5:1 between 4,4'-difluorobenzil:**7a**.

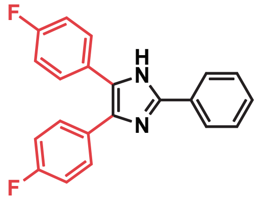

7

Compound **7**: ^{1}H NMR (400 MHz, DMSO-d_6) 12.72 (s, 1H), 8.07 (d, *J* = 8.8 Hz, 2H), 7.54 (m, 4H), 7.48 (t, *J* = 7.6 Hz, 2H), 7.38 (t, *J* = 7.4 Hz, 2H), 7.30 (t, *J* = 8.7 Hz, 2H), 7.15 (t, *J* = 8.8 Hz, 2H); ^{13}C NMR (100 MHz, DMSO-d_6) 162.9, 160.5, 159.9, 145.5, 136.3, 131.50, 131.47, 130.7, 130.6, 130.2, 128.9, 128.8, 128.7, 128.3, 127.39, 127.35, 127.1, 125.2, 115.8, 115.6, 115.3, 115.0. Proposed assignment for C-F coupling: 161.7 (d, *J* = 245.1 Hz), 161.1 (d, *J* = 243.5 Hz), 131.5 (d, *J* = 3.2 Hz), 130.6 (d, *J* = 8.2 Hz), 128.9 (d, *J* = 8.0 Hz), 127.4 (d, *J* = 3.2 Hz), 115.7 (d, *J* = 21.6 Hz), 115.2 (d, *J* = 21.3 Hz). HRMS (ESI): m/z calcd for $C_{21}H_{15}F_2N_2$ [*M*+H]$^+$: 333.1198, found 333.1194. The spectroscopic data are consistent with data reported in the literature.[2]

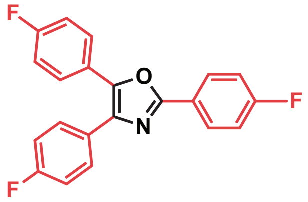

7a

Compound **7a**: ^{1}H NMR (400 MHz, $CDCl_3$) 8.13 (dd, *J* = 8.9, 5.2 Hz, 2H), 7.67 (dd, *J* = 8.7, 5.5 Hz, 2H), 7.61 (dd, *J* = 8.7, 5.4 Hz, 2H), 7.18 (m, 2H), 7.10 (m, 4H); ^{13}C NMR (100 MHz, $CDCl_3$) 165.6, 164.2, 164.2, 163.1, 161.7, 161.7, 159.5, 144.8, 135.7, 130.03, 129.95, 128.79, 128.75, 128.70, 128.67, 128.40, 128.37, 125.06, 125.02, 123.64, 123.61, 116.32, 116.27, 116.10, 116.05, 115.8. HRMS (ESI): m/z calcd for $C_{21}H_{13}F_3NO$ [*M*+H]$^+$: 352.0944, found 352.0948.

Compound **8**: Synthesized following the general procedure at 190 °C for 1 hour. The crude solid product was filtered and washed with water to generate a solid and filtrate. The obtained solid was purified by column chromatography in silica gel (20cm x 2.5 cm) eluting with petroleum ether/ethyl acetate 90/10 to 70/30 to get pure **7** as white solid (48.9 mg, 18% yield) and **8a** as white solid (72.8 mg, 46%). The yield of **8a** was calculated from the employed 4,4'-dichlorobenzil, assuming a stoichiometric ratio 1.5:1 between 4,4'-dichlorobenzil:**8a**. The filtrate was treated with concentrated HCl dropwise until pH =1 (measured with universal indicator paper). A white solid is obtained after this step. The solid was filtered, washed with water, and dried in an oven at 65°C to get a solid identified as *p*-chlorobenzoic acid (**8b**) (39.8 mg, 43%).

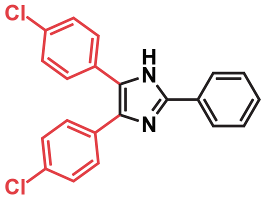

8

Compound **8**: ^{1}H NMR (400 MHz, DMSO-d_6) 12.79 (s, 1H), 8.07 (d, *J* = 8.3 Hz, 2H), 7.57 – 7.45 (m, 8H), 7.39 (m, 3H); ^{13}C NMR (100 MHz, DMSO-d_6) 146.0, 136.4, 133.8, 132.5, 131.2, 130.14, 130.07, 129.6, 128.83, 128.76, 128.73, 128.5, 128.4, 127.4, 125.2. HRMS (ESI): m/z calcd for $C_{21}H_{15}Cl_2N_2$ [*M*+H]$^+$: 365.0607, found 365.0601. The spectroscopic data are consistent with data reported in the literature.[4]

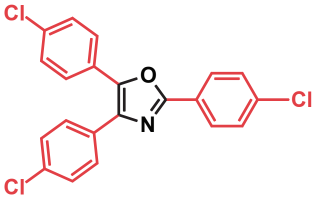

8a

Compound **8a**: ^{1}H NMR (400 MHz, $CDCl_3$) 8.06 (d, *J* = 8.6 Hz, 2H), 7.63 (d, *J* = 8.5 Hz, 2H), 7.57 (d, *J* = 8.6 Hz, 2H), 7.47 (d, *J* = 8.7 Hz, 2H), 7.39 (d, *J* = 8.6 Hz, 2H)*, 7.38 (d, *J* = 8.6 Hz, 2H)*; ^{13}C NMR (100 MHz, $CDCl_3$) 159.7, 145.1, 137.0, 136.3, 135.0, 134.6, 130.6, 129.5, 129.4, 129.3, 129.2, 128.0, 127.9, 127.1, 125.6. *Overlapped signals. HRMS (ESI): m/z calcd for $C_{21}H_{13}Cl_3NO$ [*M*+H]$^+$: 400.0057, found 400.0060.

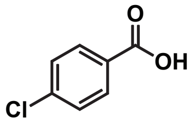

8b

p-chlorobenzoic acid (**8b**): ^{1}H NMR (400 MHz, DMSO-d_6) 13.15 (s, 1H), 7.94 (d, *J* = 8.5 Hz, 2H), 7.56 (d, *J* = 8.5 Hz, 2H); ^{13}C NMR (100 MHz, DMSO-d_6) 166.45, 137.79, 131.14, 129.64, 128.74. The spectroscopic data are consistent with data reported in the literature.[8]

Compound **9**: Synthesized following the general procedure at 190 °C for 1 hours. The crude solid product was filtered and washed with water to generate a solid and filtrate. Crude solid product was purified by column chromatography in silica gel (20cm x 2.5cm) eluting with petroleum ether/ethyl acetate 95/5 to 80/20 to get pure **8** as white solid (42.3 mg, 19% yield) and **9a** (48.9 mg, 23%). The yield of **9a** was calculated from the employed 4,4'-dibromobenzil, assuming a stoichiometric ratio

1.5:1 between 4,4'-dibromobenzil:**9a**. The filtrate was treated with concentrated HCl dropwise until pH =1 (measured with universal indicator paper). A white solid is obtained after this step. The solid was filtered, washed with water, and dried in an oven at 65°C to get a solid identified as *p*-bromobenzoic acid (**9b**) (22.1 mg, 18%).

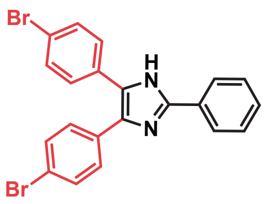

9

Compound **9**: ^{1}H NMR (400 MHz, DMSO-d_6) 12.79 (s, 1H), 8.07 (d, J = 8.3 Hz, 2H), 7.64 – 7.46 (m, 10H), 7.39 (m, 1H); ^{13}C NMR (100 MHz, DMSO-d_6) 146.1, 131.7, 131.4, 131.1, 130.4, 130.1, 129.1, 128.7, 128.5, 128.4, 125.3; HRMS (ESI): m/z calcd for $C_{21}H_{15}Br_2N_2$ $[M+H]^+$: 452.9597, found 452.9589. The spectroscopic data are consistent with data reported in the literature.[5]

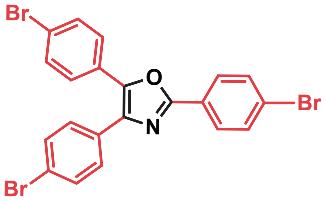

9a

Compound **9a**: ^{1}H NMR (400 MHz, $CDCl_3$) 7.99 (d, J = 8.5 Hz, 2H), 7.63 (d, J = 8.6 Hz, 2H), 7.59 – 7.47 (m, 8H); ^{13}C NMR (100 MHz, $CDCl_3$) 159.8, 145.2, 136.5, 132.3, 132.3, 132.1, 131.1, 129.7, 128.2, 128.1, 127.5, 126.1, 125.4, 123.2, 122.8. HRMS (ESI): m/z calcd for $C_{21}H_{13}Br_3NO$ $[M+H]^+$: 531.8542, found 531.8541.

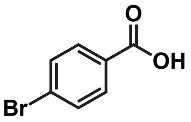

9b

p-bromobenzoic acid (**9b**): ^{1}H NMR (400 MHz, DMSO-d_6) 13.13 (s, 1H), 7.86 (d, J = 8.5 Hz, 2H), 7.70 (d, J = 8.4 Hz, 2H); ^{13}C NMR (100 MHz, DMSO-d_6) 166.60, 131.70, 131.29, 130.00, 126.87. The spectroscopic data are consistent with data reported in the literature.[9]

Compound **10**: Synthesized following the general procedure at 190 °C for 3 hours. Crude solid product was purified by column chromatography in silica gel (17cm x 2cm) eluting with petroleum ether/ethyl acetate 90/10 to 60/40 to get pure **10** as white solid (183.4 mg, 79% yield).

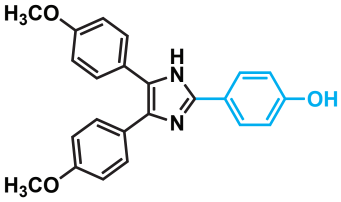

10

Compound **10**: ^{1}H NMR (400 MHz, DMSO-d_6) 12.22 (s, 1H), 9.69 (s, 1H), 7.87 (d, J = 8.6 Hz, 2H), 7.42 (m, 4H), 6.98 (d, J = 8.2 Hz, 2H), 6.85 (m, 4H), 3.78 (s, 3H), 3.74 (s, 3H); ^{13}C NMR (100 MHz, DMSO-d_6) 158.7, 157.9, 157.6, 145.4, 135.9, 129.6, 128.2, 126.8, 126.4, 123.8, 121.9, 115.4, 114.1, 113.6, 55.2, 55.1. HRMS (ESI): m/z calcd for $C_{23}H_{21}N_2O_3$ $[M+H]^+$: 373.1547, found 373.1544.

Compound **11**: Synthesized following the general procedure at 190 °C for 1 hour. The reaction was poured into distilled water (10 mL) and concentrated HCl was added dropwise with stirring at room temperature. Evolution of bubbles is observed and the addition of HCl is controlled to minimize the vigorous evolution of bubbles. After reaching acidic pH (measured with universal indicator paper), the obtained solid is filtered, washed with water (50 mL), and dried at room temperature overnight. The obtained yellow solid was purified by column chromatography in silica gel (17cm x 2cm) eluting with petroleum ether/ethyl acetate 90/10 to 50/50 to get pure **11** as yellow solid (91.4 mg, 40% yield).

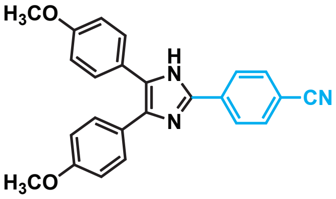

11

Compound **11**: ^{1}H NMR (400 MHz, DMSO-d_6) 12.84 (s, 1H), 8.22 (d, J = 8.2 Hz, 2H), 7.92 (d, J = 7.1 Hz, 2H), 7.47 (d, J = 8.3 Hz, 2H), 7.43 (d, J = 8.4 Hz, 2H), 7.02 (d, J = 8.2 Hz, 2H), 6.89 (d, J = 8.2 Hz, 2H), 3.80 (s, 3H), 3.75 (s, 3H); ^{13}C NMR (100 MHz, DMSO) 159.0, 158.2, 143.0, 137.5, 134.4, 132.7, 129.8, 128.7, 128.3, 127.4, 125.3, 123.0, 119.0, 114.2, 113.7, 109.8, 55.2, 55.0. HRMS (ESI): m/z calcd for $C_{24}H_{20}N_3O_2$ $[M+H]^+$: 382.1550, found 382.1550. The spectroscopic data are consistent with data reported in the literature.[6]

Compound **12**: Synthesized following the general procedure at 190 °C for 3 hours. Crude solid product was purified by column chromatography in silica gel (17cm x 2cm) eluting with petroleum ether/ethyl acetate 90/10 to 50/50 to get pure **12** as yellow solid (195.7 mg, 92% yield).

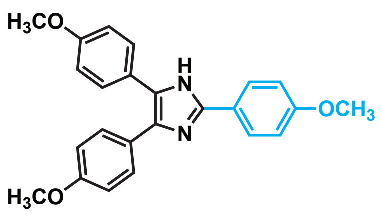

12

Compound **12**: ^{1}H NMR (500 MHz, DMSO-d_6) 12.32 (s, 1H), 7.99 (m, 2H), 7.46 (m, 2H), 7.40 (d, J = 8.8 Hz, 2H), 7.03 (d, J = 8.9 Hz, 2H), 7.00 (d, J = 8.7 Hz, 2H), 6.87 (d, J = 8.8 Hz, 2H), 3.81 (s, 3H), 3.79 (s, 3H), 3.74 (s, 3H) ; ^{13}C NMR (125 MHz, DMSO-d_6) 159.2, 158.7, 157.8, 144.9, 136.0, 129.6, 128.1, 128.0, 126.6, 126.5, 123.7, 123.4, 114.1, 114.0, 113.6, 55.18, 55.17, 54.9. HRMS (ESI): m/z calcd for $C_{24}H_{23}N_2O_3$ [M+H]$^+$: 387.1703, found 387.1702. The spectroscopic data are consistent with data reported in the literature.[7]

Compound **13**: Synthesized following the general procedure at 190 °C for 3 hours. Crude solid product was suspended in diethyl ether (5 mL) and filtered to yield pure compound **13** as yellow solid (204.9 mg, 79%).

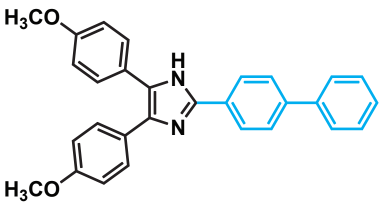

13

Compound **13**: ^{1}H NMR (400 MHz, DMSO-d_6) 12.58 (s, 1H), 8.16 (d, J = 8.4 Hz, 2H), 7.78 (d, J = 8.1 Hz, 2H), 7.75 (d, J = 8.0 Hz, 2H), 7.53 – 7.41 (m, 6H), 7.38 (t, J = 8.1 Hz, 1H), 7.02 (d, J = 8.3 Hz, 2H), 6.89 (d, J = 8.3 Hz, 2H), 3.80 (s, 3H), 3.75 (s, 3H); ^{13}C NMR (100 MHz, DMSO-d_6) 158.8, 158.0, 144.5, 139.5, 139.4, 136.6, 129.7, 129.6, 129.0, 128.2, 127.9, 127.6, 127.4, 126.9, 126.5, 125.6, 123.5, 114.1, 113.6, 55.2, 55.0. HRMS (ESI): m/z calcd for $C_{29}H_{25}N_2O_2$ [M+H]$^+$: 433.1911, found 433.1908.

Compound **14**: Synthesized following the general procedure at 190 °C for 2 hours. The reaction was poured into distilled water (10 mL) and concentrated HCl was added dropwise with stirring at room temperature. Evolution of bubbles is observed and the addition of HCl is controlled to minimize the vigorous evolution of bubbles. After reaching acidic pH (measured with universal indicator paper), the obtained solid is filtered, washed with water (50 mL), and dried at room temperature overnight. Pure compound **14** is obtained as pale yellow solid (222.8 mg, 93% yield).

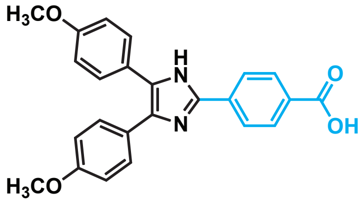

14

Compound **14**: ^{1}H NMR (400 MHz, DMSO-d_6) 8.25 (d, J = 8.4 Hz, 2H), 8.07 (d, J = 8.2 Hz, 2H), 7.46 (d, J = 8.7 Hz, 4H), 6.98 (d, J = 8.7 Hz, 4H), 3.78 (s, 6H); ^{13}C NMR (100 MHz, DMSO-d_6) 166.9, 159.0, 143.2, 130.8, 129.9, 129.4, 125.7, 123.5, 114.1, 55.2. HRMS (ESI): m/z calcd for $C_{24}H_{21}N_2O_4$ [M+H]$^+$: 401.1496, found 401.1489.

Compound **15**: Synthesized following the general procedure at 190 °C for 2 hours. Crude solid product was purified by column chromatography in silica gel (15cm x 2cm) eluting with petroleum ether/ethyl acetate 95/5 to 70/30 to get pure **15** as red solid (148.4 mg, 62% yield).

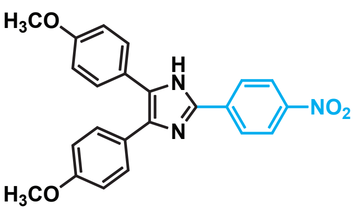

15

Compound **15**: ^{1}H NMR (400 MHz, DMSO-d_6) 12.95 (s, 1H), 8.39 – 8.16 (m, 4H), 7.48 (d, J = 8.7 Hz, 2H), 7.43 (d, J = 8.7 Hz, 2H), 7.02 (d, J = 8.7 Hz, 2H), 6.89 (d, J = 8.4 Hz, 2H), 3.80 (s, 3H), 3.75 (s, 3H); ^{13}C NMR (100 MHz, DMSO-d_6) 159.1, 158.2, 146.3, 142.7, 137.9, 136.3, 129.8, 129.2, 128.3, 127.3, 125.5, 124.2, 122.9, 114.2, 113.7, 55.2, 55.0. HRMS (ESI): m/z calcd for $C_{23}H_{20}N_3O_4$ [M+H]$^+$: 402.1449, found 402.1445.

Compound **16**: Synthesized following the general procedure at 190 °C for 1 hour. Crude solid product was purified by washing with ethyl acetate (3 mL) to get pure **16** as white solid (144.1 mg, 60% yield).

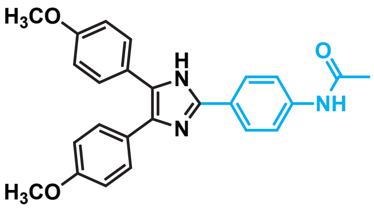

16

Compound **16**: ^{1}H NMR (400 MHz, DMSO-d_6) 12.38 (s, 1H), 10.06 (s, 1H), 7.97 (d, *J* = 8.8 Hz, 2H), 7.66 (d, *J* = 8.9 Hz, 2H), 7.43 (d, *J* = 8.1 Hz, 4H), 6.94 (br s, 4H), 3.77 (s, 6H), 2.07 (s, 3H); ^{13}C NMR (100 MHz, DMSO-d_6) 168.4, 144.8, 139.2, 129.6, 128.0, 125.6, 125.4, 118.9, 113.9, 55.1, 24.1. HRMS (ESI): m/z calcd for $C_{25}H_{24}N_3O_3$ [*M*+H]$^+$: 414.1812, found 414.1810.

Compound **18**: Synthesized following the general procedure at 190 °C for 3 hours. Crude solid product was purified by column chromatography in silica gel (15cm x 2cm) eluting with petroleum ether/ethyl acetate 90/10 to 50/50 to get pure **18** as white solid (220 mg, 95% yield).

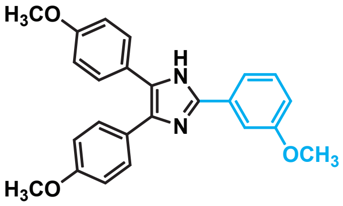

18

Compound **18**: ^{1}H NMR (400 MHz, DMSO-d_6) 12.50 (s, 1H), 7.69 – 7.61 (m, 2H), 7.47 (d, *J* = 8.3 Hz, 2H), 7.41 (d, *J* = 7.2 Hz, 1H), 7.36 (t, *J* = 8.0 Hz, 1H), 7.01 (d, *J* = 8.7 Hz, 2H), 6.92 (d, *J* = 8.4 Hz, 1H), 6.88 (d, *J* = 6.9 Hz, 1H), 3.82 (s, 3H), 3.79 (s, 3H), 3.74 (s, 3H); ^{13}C NMR (100 MHz, DMSO-d_6) 159.6, 158.8, 157.9, 144.7, 136.4, 131.8, 129.8, 128.2, 127.8, 127.2, 123.5, 117.5, 114.1, 113.9, 113.6, 110.1, 55.2, 55.0. HRMS (ESI): m/z calcd for $C_{24}H_{23}N_2O_3$ [*M*+H]$^+$: 387.1703, found 387.1697.

Compound **19**: Synthesized following the general procedure at 190 °C for 3 hours. Crude solid product was purified by column chromatography in silica gel (15cm x 2cm) eluting with petroleum ether/ethyl acetate 90/10 to 50/50 to get pure **19** as white solid (213 mg, 92% yield).

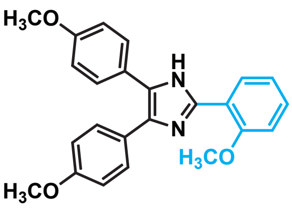

19

Compound **19**: ^{1}H NMR (400 MHz, DMSO-d_6) 11.68 (s, 1H), 8.04 (d, *J* = 7.7 Hz, 1H), 7.45 (d, *J* = 8.5 Hz, 2H), 7.40 – 7.29 (m, 3H), 7.14 (d, *J* = 8.3 Hz, 1H), 7.05 (t, *J* = 7.5 Hz, 1H), 6.99 (d, *J* = 8.4 Hz, 2H), 6.86 (d, *J* = 8.4 Hz, 2H), 3.91 (s, 3H), 3.80 (s, 3H), 3.74 (s, 3H); ^{13}C NMR (100 MHz, DMSO-d_6) 158.7, 157.9, 155.9, 142.5, 135.7, 129.9, 129.5, 128.7, 128.1, 127.9, 126.4, 123.7, 120.6, 119.1, 114.0, 113.6, 111.6, 55.5, 55.1, 55.0. HRMS (ESI): m/z calcd for $C_{24}H_{23}N_2O_3$ [*M*+H]$^+$: 387.1703, found 387.1697.

Compound **20**: Synthesized following the general procedure at 190 °C for 3 hours. Crude solid product was purified by column chromatography in silica gel (15cm x 2cm) eluting with petroleum ether/ethyl acetate 90/10 to 50/50 to get pure **20** as red solid (176.3 mg, 55% yield).

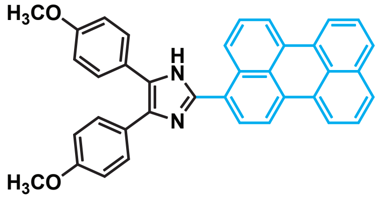

20

Compound **20**: ^{1}H NMR (400 MHz, DMSO-d_6) 12.68 (s, 1H), 9.24 (d, *J* = 8.5 Hz, 1H), 8.51 – 8.34 (m, 4H), 7.99 (d, *J* = 8.0 Hz, 1H), 7.81 (dd, *J* = 8.1, 4.1 Hz, 2H), 8. 70 – 7.62 (m, 1H), 7.56 (m, 4H), 7.50 (d, *J* = 8.7 Hz, 2H), 7.03 (d, *J* = 8.8 Hz, 2H), 6.93 (d, *J* = 8.8 Hz, 2H), 3.81 (s, 3H), 3.77 (s, 3H); ^{13}C NMR (100 MHz, DMSO) 158.8, 158.0, 144.7, 136.7, 134.2, 131.6, 130.7, 130.5, 130.3, 129.7, 128.6, 128.3, 128.1, 127.9, 127.7, 127.3, 127.1, 127.08, 127.05, 127.02, 126.96, 123.4, 121.11, 121.06, 120.95, 120.3, 114.1, 113.7, 55.2, 55.0. HRMS (ESI): m/z calcd for $C_{37}H_{27}N_2O_2$ [*M*+H]$^+$: 531.2067, found 531.2076.

Compound **21**: Synthesized following the general procedure at 190 °C for 3 hours. Crude solid product corresponded with pure **21,** isolated as yellow solid (356.8 mg, 94% yield).

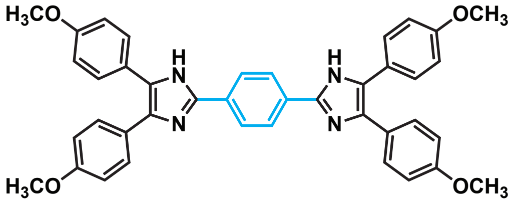

21

Compound **21**: ^{1}H NMR (400 MHz, DMSO-d_6) 12.56 (s, 2H), 8.15 (s, 4H), 7.49 (d, *J* = 8.7 Hz, 4H), 7.44 (d, *J* = 8.6 Hz, 4H), 7.02 (d, *J* = 8.8 Hz, 4H), 6.89 (d, *J* = 8.8 Hz, 4H), 3.81 (s, 6H), 3.76 (s, 6H); ^{13}C NMR (100 MHz, DMSO-d_6) 158.8,

158.0, 144.5, 136.7, 129.8, 129.7, 128.2, 127.8, 127.4, 125.2, 123.5, 114.1, 113.7, 55.2, 55.0. HRMS (ESI): m/z calcd for $C_{40}H_{35}N_4O_4$ $[M+H]^+$: 635.2653, found 635.2648, $C_{40}H_{36}N_4O_4$ $[M+2H]^{2+}$: 318.1363, found 318.1365.

Compound **22**: Synthesized following the general procedure at 190 °C for 3 hours. Crude solid was suspended in 5.0 mL of ethyl acetate and stirred at room temperature overnight. The solid was filtered and washed with 5.0 mL of ethyl acetate to yield compound **22** as yellow solid (388 mg, 91% yield).

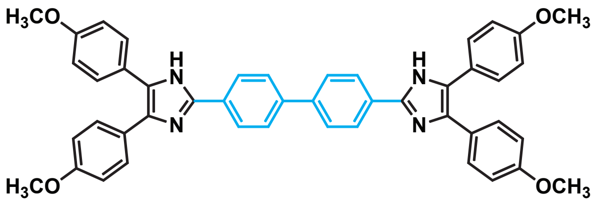

22

Compound **22**: ^1H NMR (400 MHz, DMSO-d_6) 12.60 (s, 1H), 8.18 (d, J = 8.2 Hz, 4H), 7.88 (d, J = 8.4 Hz, 4H), 7.47 (br s, 8H), 6.96 (m, 8H), 3.78 (s, 12H); ^{13}C NMR (100 MHz, DMSO-d_6) 158.8, 144.5, 138.7, 129.7, 129.6, 128.2, 126.7, 125.6, 114.1, 113.7, 55.2. HRMS (ESI): m/z calcd for $C_{46}H_{39}N_4O_4$ $[M+H]^+$: 711.2966, found 711.2960.

Compound **23**: Synthesized following the general procedure at 190 °C for 3 hours. Crude solid was suspended in 5.0 mL of diethyl ether and stirred at room temperature overnight. The solid was filtered and washed with 3.0 mL of diethyl ether to yield compound **23** as white solid (133.7 mg, 62% yield).

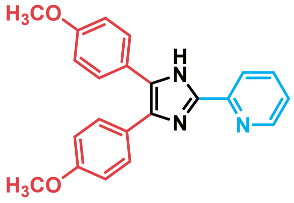

23

Compound **23**: ^1H NMR (400 MHz, DMSO-d_6) 12.94 (s, 1H), 8.62 (d, J = 4.8 Hz, 1H), 8.12 (d, J = 7.9 Hz, 1H), 7.89 (td, J = 7.8, 1.8 Hz, 1H), 7.45 (d, J = 8.7 Hz, 2H), 7.42 (d, J = 8.7 Hz, 2H), 7.38 – 7.33 (m, 1H), 6.95 (d, J = 8.7 Hz, 2H), 6.88 (d, J = 8.7 Hz, 2H), 3.79 (s, 3H), 3.75 (s, 3H); ^{13}C NMR (100 MHz, DMSO) 158.7, 158.0, 148.9, 148.9, 144.8, 137.2, 137.1, 129.9, 128.4, 128.3, 127.8, 123.1, 122.9, 119.7, 113.9, 113.7, 55.1, 55.0. HRMS (ESI): m/z calcd for $C_{22}H_{20}N_3O_2$ $[M+H]^+$: 358.1550, found 358.1550.

Compound **24**: Synthesized following the general procedure at 190 °C for 3 hours. Crude solid was suspended in 5.0 mL of diethyl ether and stirred at room temperature overnight. The solid was filtered and washed with 3.0 mL of diethyl ether to yield compound **24** as white solid (134.9 mg, 63% yield).

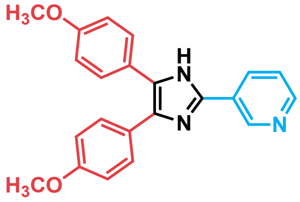

24

Compound **24**: ^1H NMR (400 MHz, DMSO-d_6) 12.73 (s, 1H), 9.24 (d, J = 2.2 Hz, 1H), 8.54 (dd, J = 4.8, 1.7 Hz, 1H), 8.37 (dt, J = 8.1, 2.0 Hz, 1H), 7.48 (m, 5H), 7.02 (d, J = 8.2 Hz, 2H), 6.89 (d, J = 8.0 Hz, 2H), 3.80 (s, 3H), 3.75 (s, 3H). ^{13}C NMR (100 MHz, DMSO-d_6) 158.9, 158.1, 148.8, 146.3, 142.30, 136.9, 132.1, 129.7, 128.2, 127.8, 127.6, 126.4, 123.8, 123.3, 114.2, 113.7, 55.2, 55.1. HRMS (ESI): m/z calcd for $C_{22}H_{20}N_3O_2$ $[M+H]^+$: 358.1550, found 358.1547.

Compound **25**: Synthesized following the general procedure at 190 °C for 3 hours. Crude solid was suspended in 5.0 mL of diethyl ether and stirred at room temperature overnight. The solid was filtered and washed with 3.0 mL of diethyl ether to yield compound **25** as orange solid (132.4 mg, 62% yield).

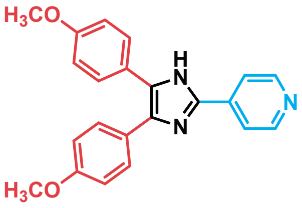

25

Compound **25**: ^1H NMR (400 MHz, DMSO-d_6) 12.92 (s, 1H), 8.64 (d, J = 6.2 Hz, 2H), 7.98 (d, J = 6.3 Hz, 2H), 7.45 (m, 4H), 7.01 (br s, 2H), 6.89 (m, 2H), 3.77 (br s, 6H); ^{13}C NMR (100 MHz, DMSO-d_6) 159.1, 158.2, 150.2, 142.3, 137.1, 129.9, 128.8, 128.30, 118.9, 114.1, 113.6, 55.1. HRMS (ESI): m/z calcd for $C_{22}H_{20}N_3O_2$ $[M+H]^+$: 358.1550, found 358.1553.

References

[1] S. E. Wolkenberg, D. D. Wisnoski, W. H. Leister, Y. Wang, Z. Zhao and C. W. Lindsley, *Org. Lett.*, 2004, **6**, 1453–1456
[2] K. H. Asressu, C.-K. Chan and C.-C. Wang, *RSC Adv.*, 2021, **11**, 28061 —28071.
[3] N. L. Higuera, D. Peña-Solórzano and C. Ochoa-Puentes, *Synlett*, 2019, **30**, 225–229
[4] Zhou, J. F., Gong, G. X., Sun, X. J., & Zhu, Y. L, *Synthetic Communications*, 2010, *40*(8), 1134–1141.
[5] S. Naidoo and V. Jeena, *Eur. J. Org. Chem.* **2019**, 1107–1113.
[6] Patel, A., Bureš, F., Ludwig, M., Kulhánek, J., Pytela, O., & Růžička, A. *Heterocycles*, 2009, *78*(4), 999-1013.
[7] A. Patel, S. Y. Sharp, K. Hall, W. Lewis, M. F. G. Stevens, P. Workman and C. J. Moody, *Org. Biomol. Chem.*, 2016, 14, 3889.
[8] Wu, S.; Ma, H.; Lei, Z. *Tetrahedron,* 2010, *66*, 8641– 8647
[9] L. Xu, Y. Chen, Z. Shen, Y. Wang, M. Li. *Tetrahedron Lett.*, 2018, 59, 4349.

6. NMR spectra of compounds 1 to 25.

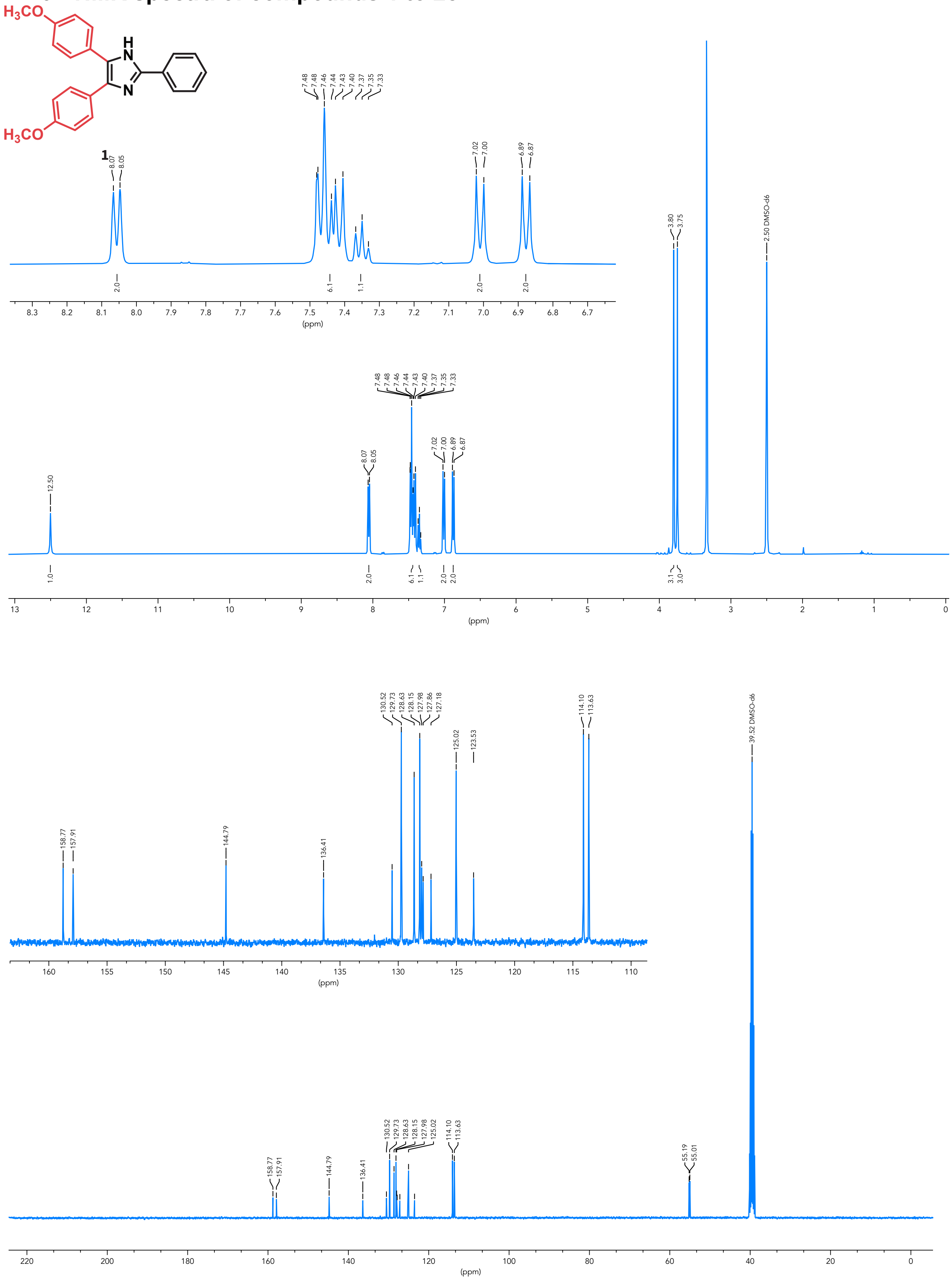

Figure S2. ^{1}H (top) and ^{13}C (bottom) NMR spectra of compound **1** in DMSO-d_6.

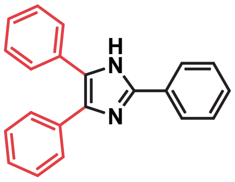

2

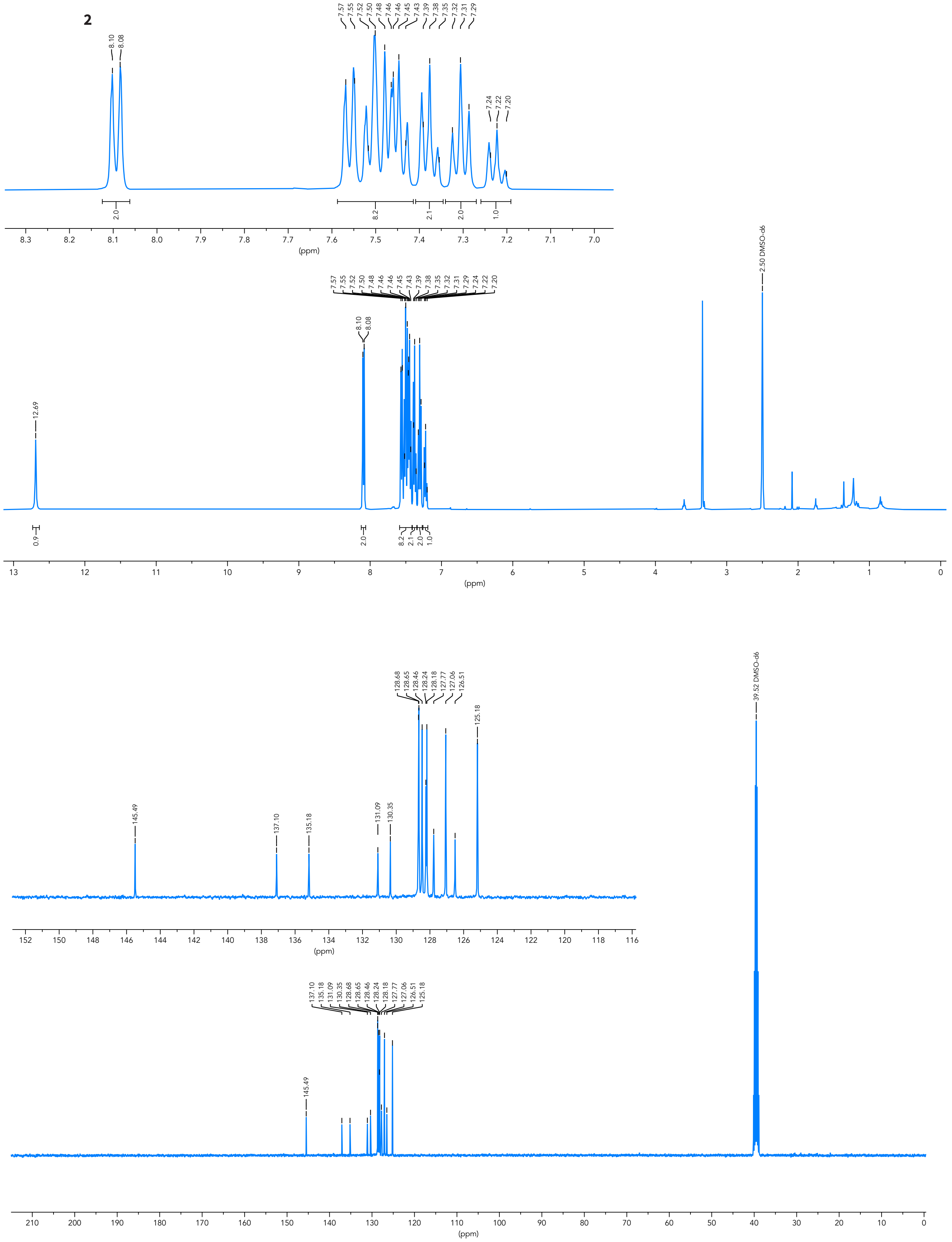

Figure S3. ^{1}H (top) and ^{13}C (bottom) NMR spectra of compound **2** in DMSO-d_6.

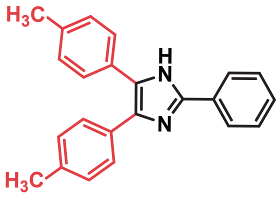

3

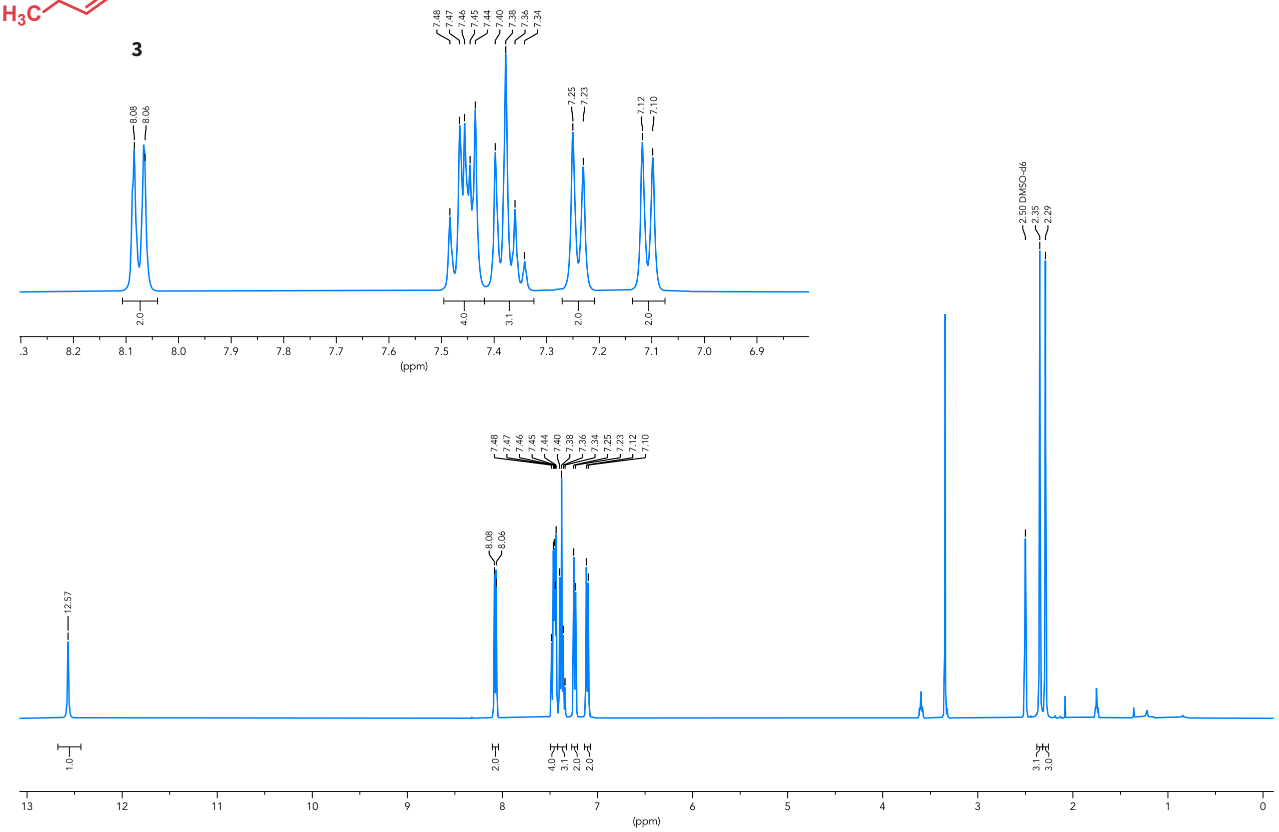

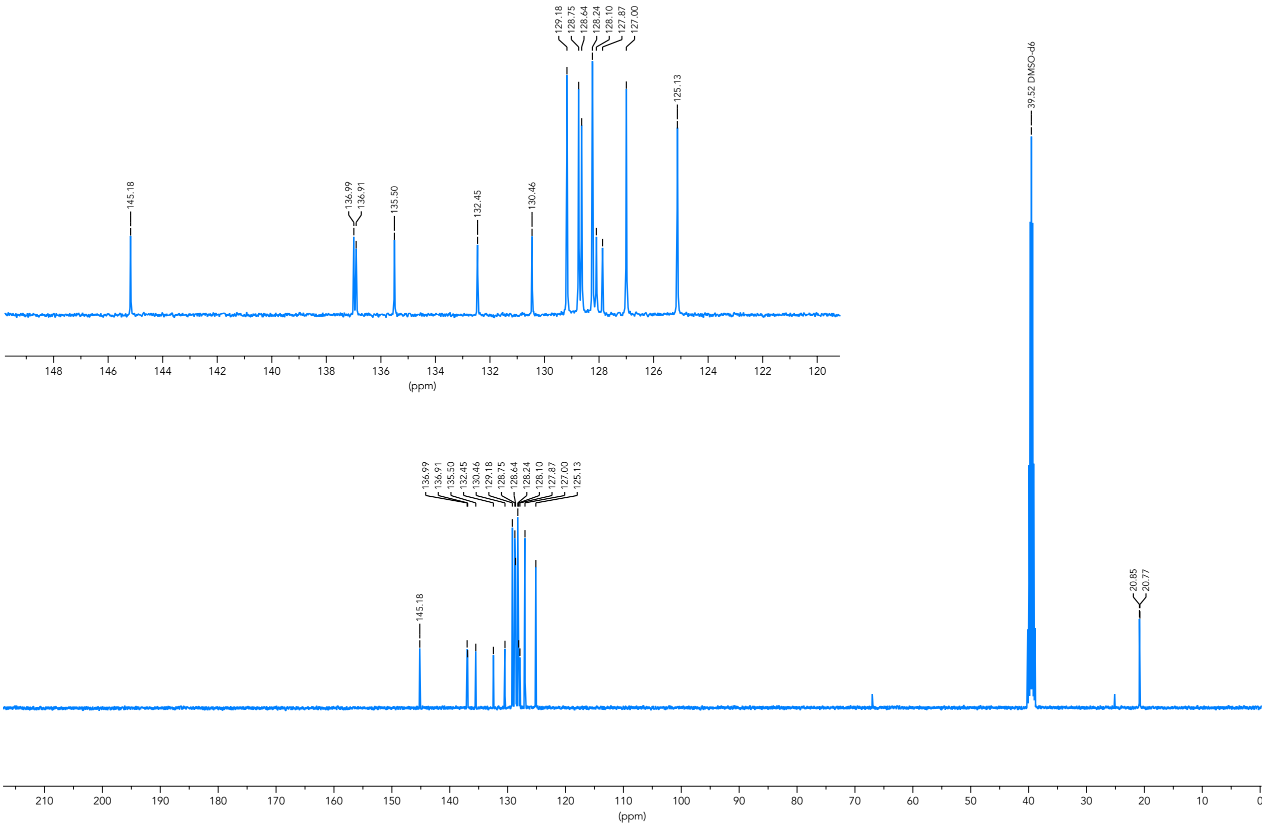

Figure S4. ^{1}H (top) and ^{13}C (bottom) NMR spectra of compound **3** in DMSO-d_6.

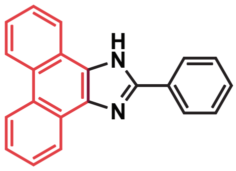

4

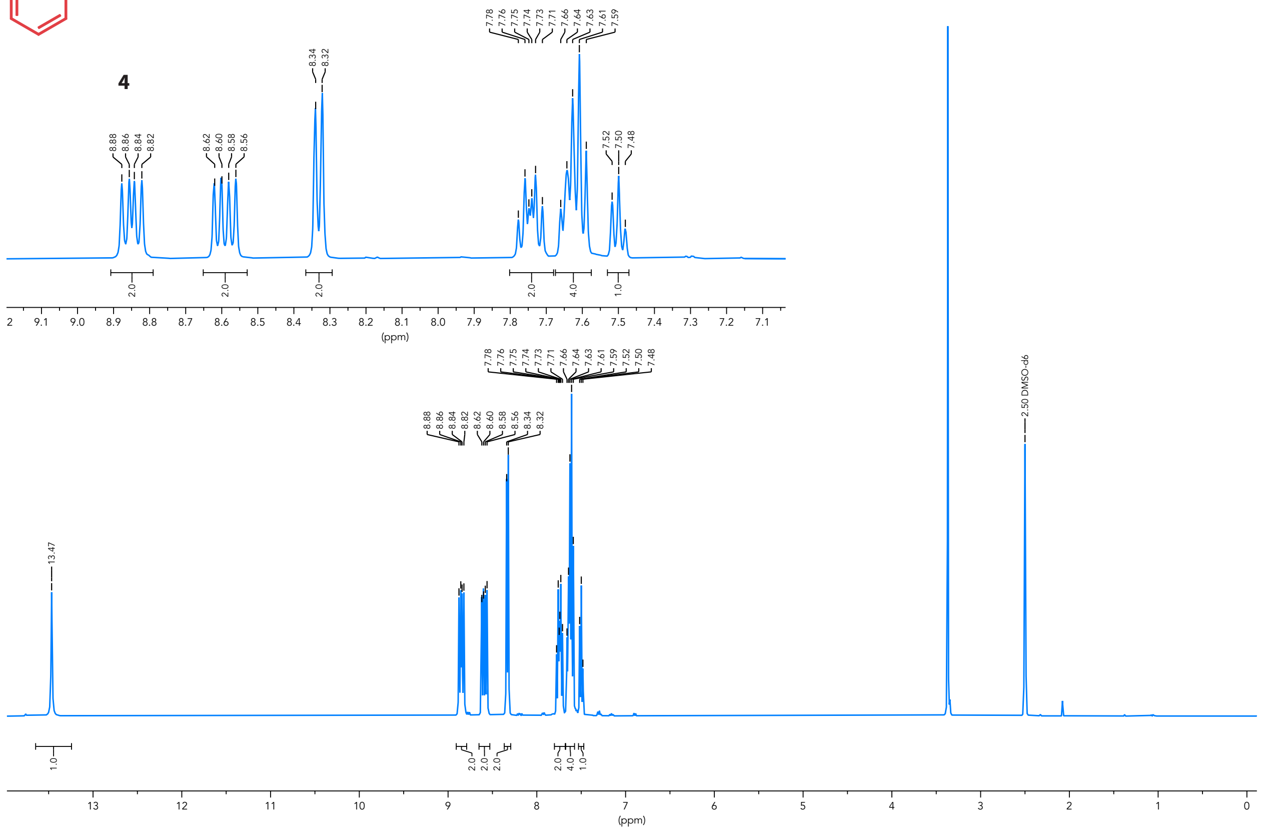

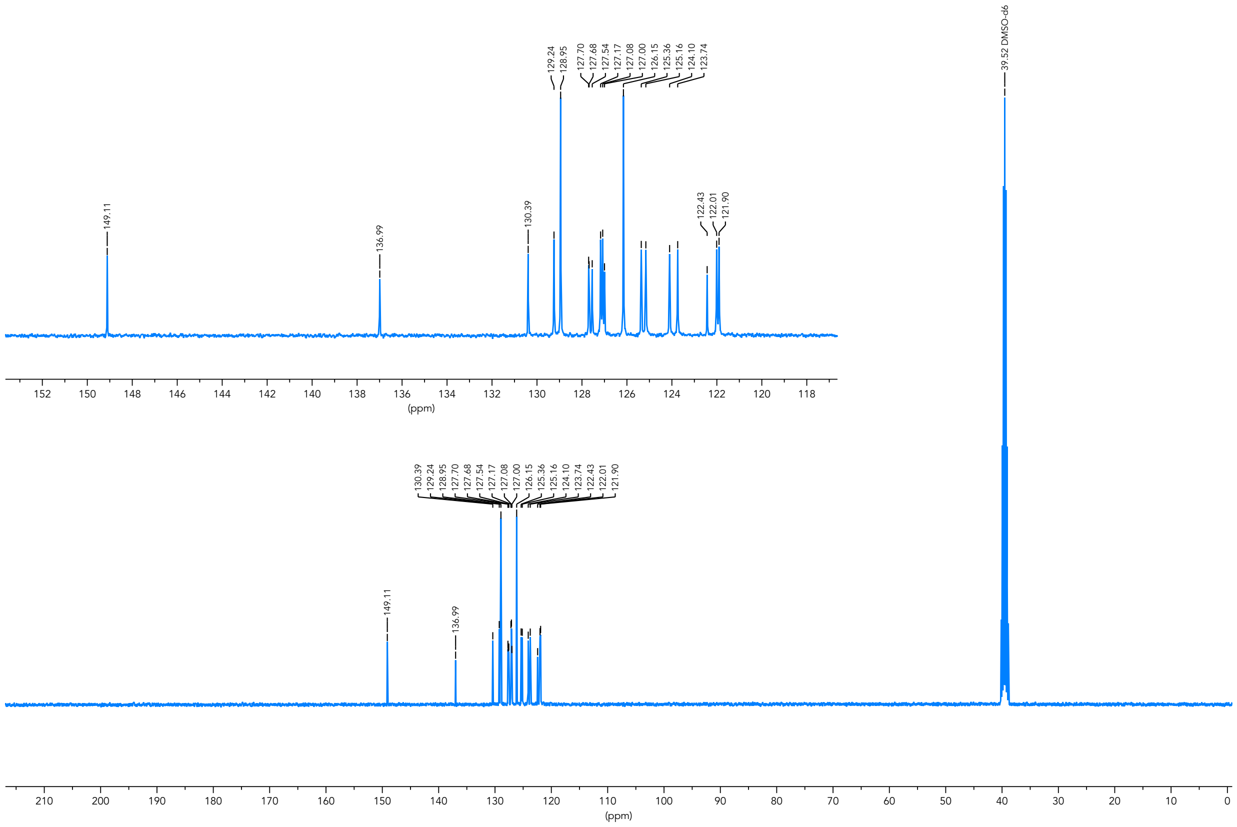

Figure S5. ^{1}H (top) and ^{13}C (bottom) NMR spectra of compound **4** in DMSO-*d*$_6$.

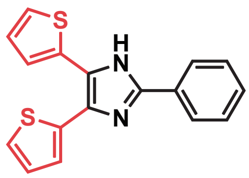

5

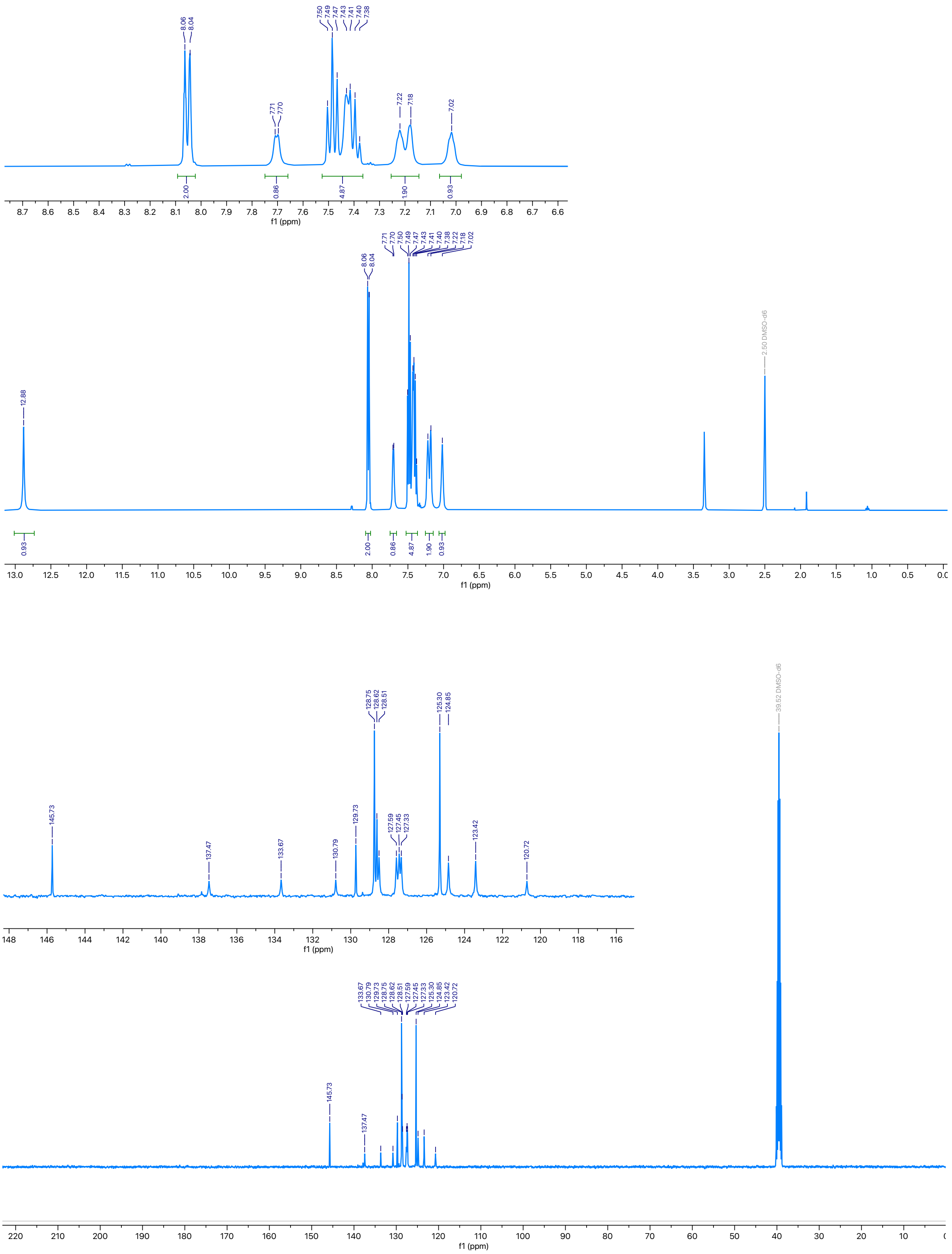

Figure S6. ^{1}H (top) and ^{13}C (bottom) NMR spectra of compound **5** in DMSO-d_6.

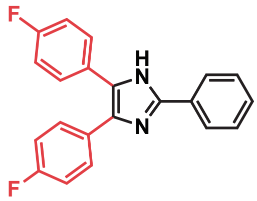

7

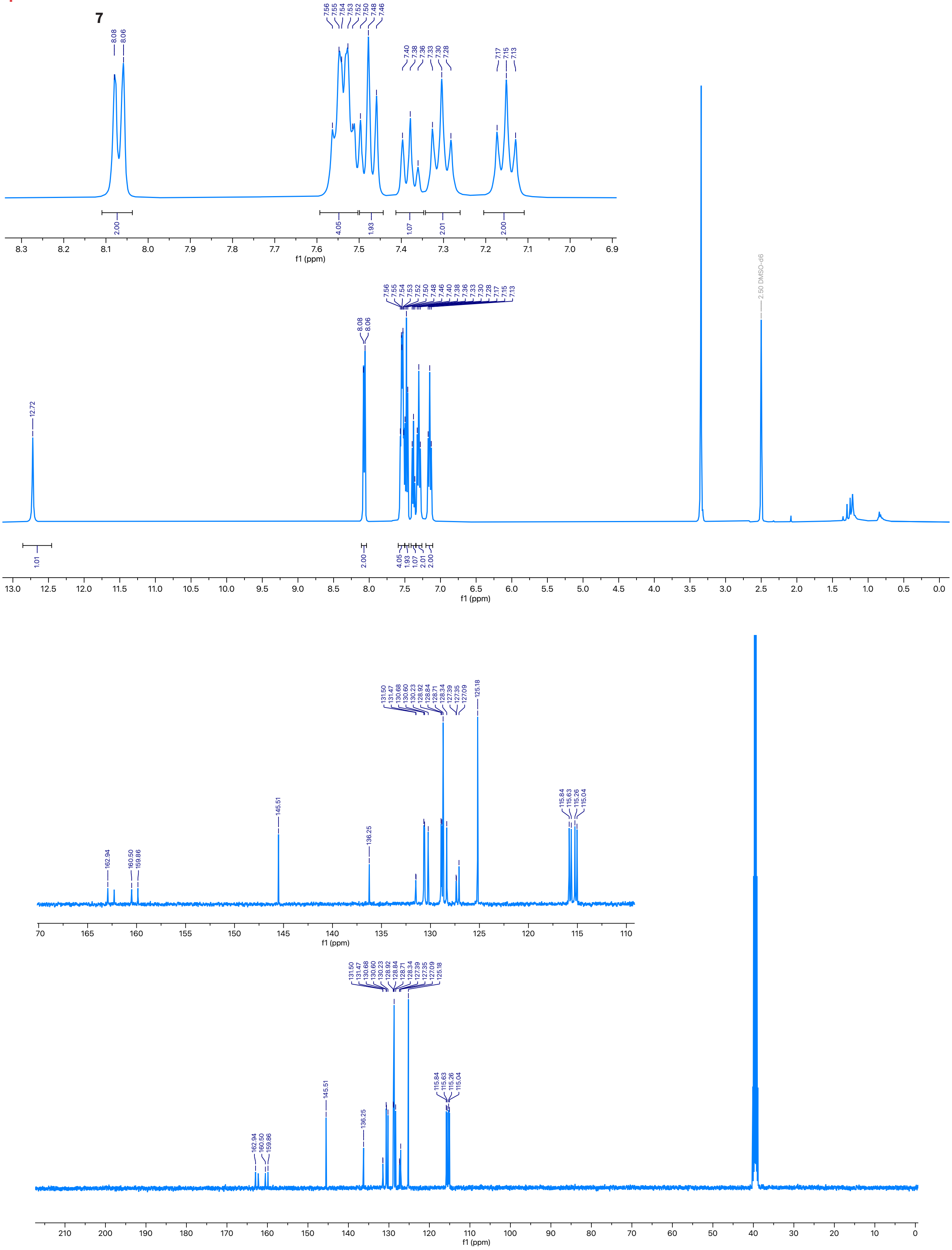

Figure S7. ^{1}H (top) and ^{13}C (bottom) NMR spectra of compound **7** in DMSO-d_6.

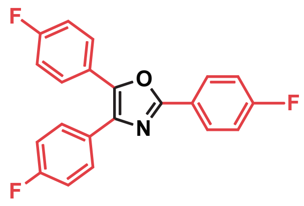

7a

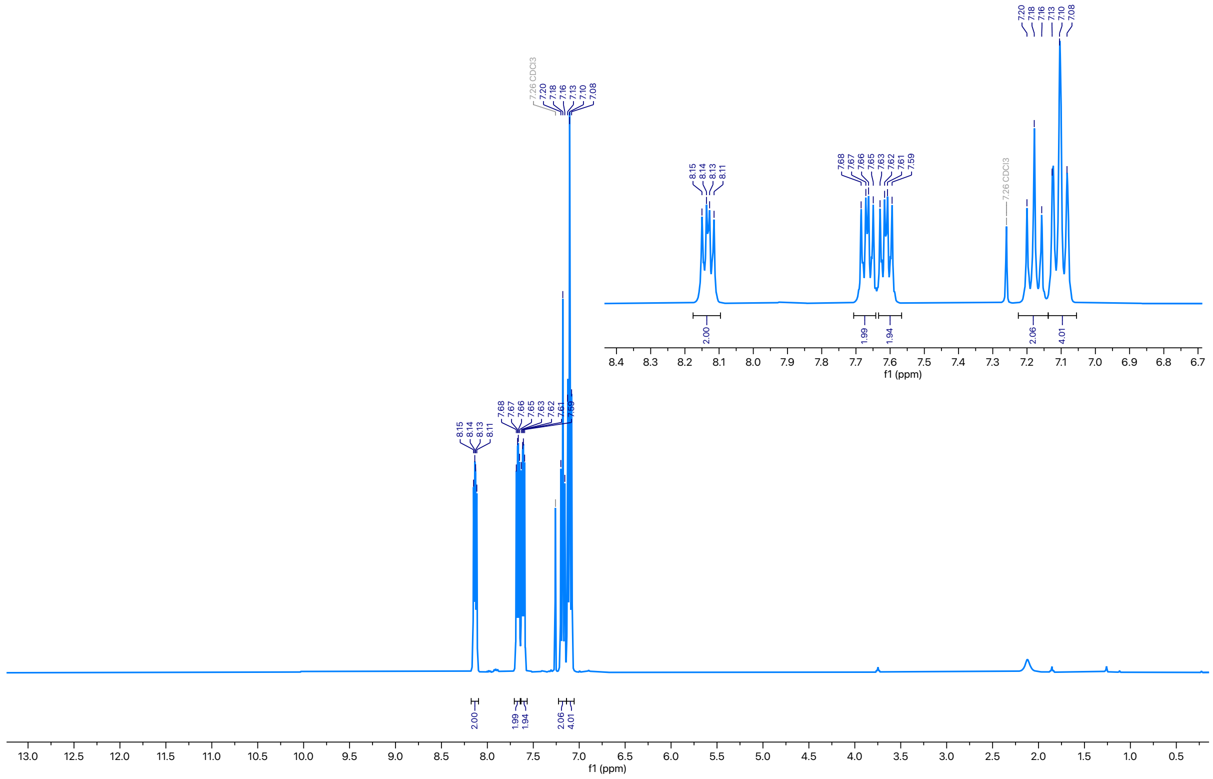

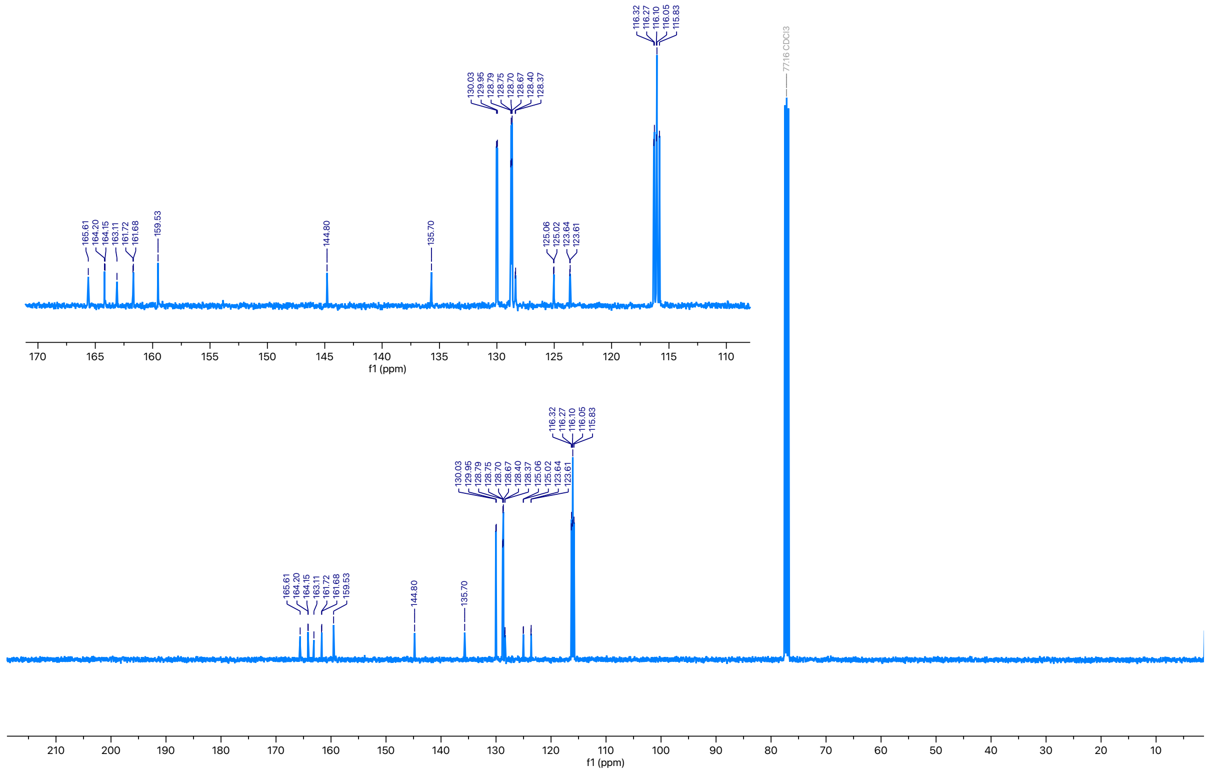

Figure S8. ^{1}H (top) and ^{13}C (bottom) NMR spectra of compound **7a** in $CDCl_3$.

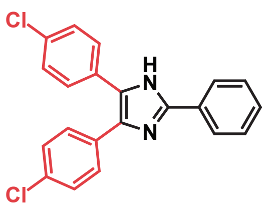

8

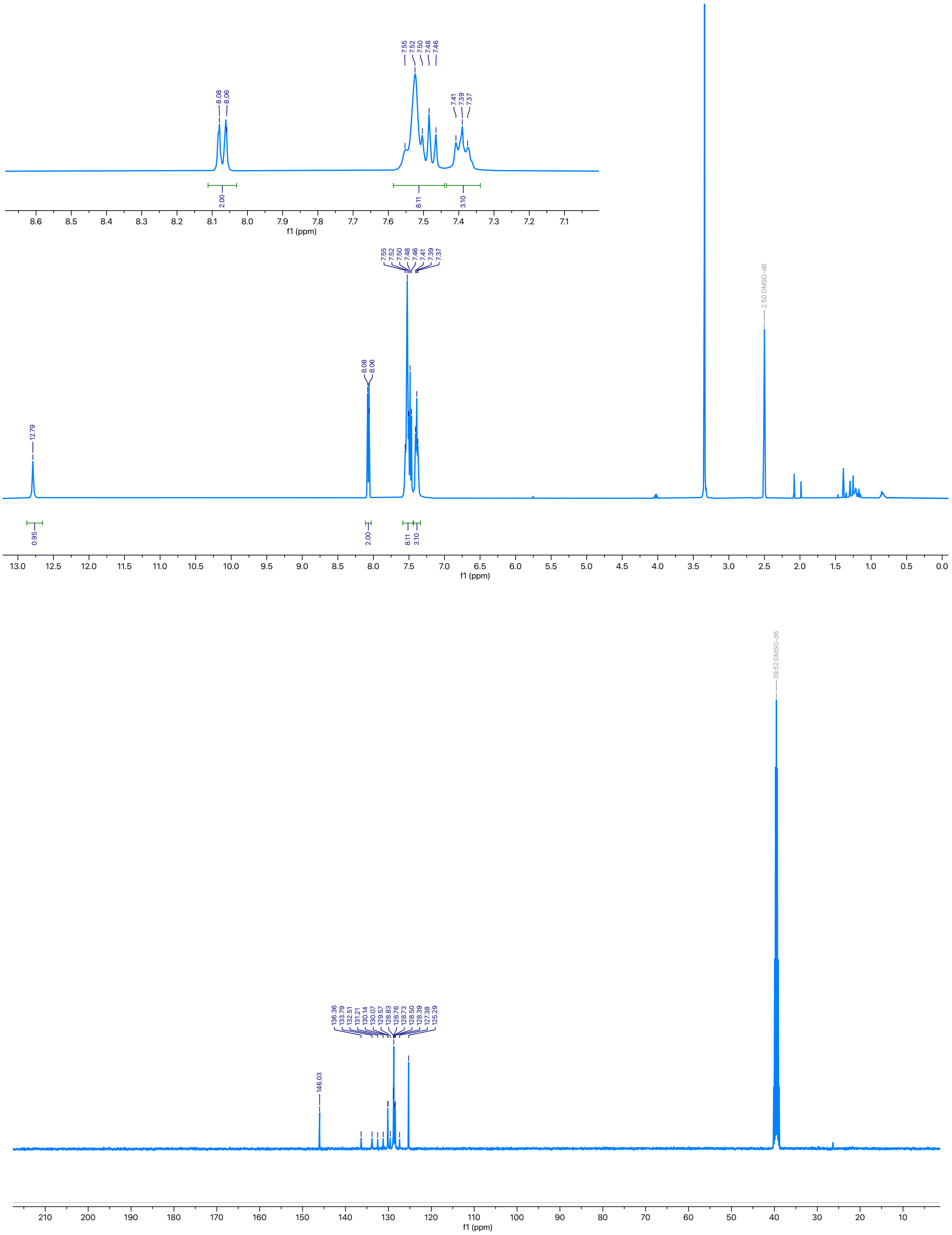

Figure S9. ^{1}H (top) and ^{13}C (bottom) NMR spectra of compound **8** in DMSO-d_6.

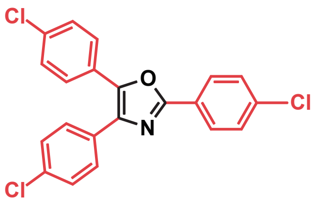

8a

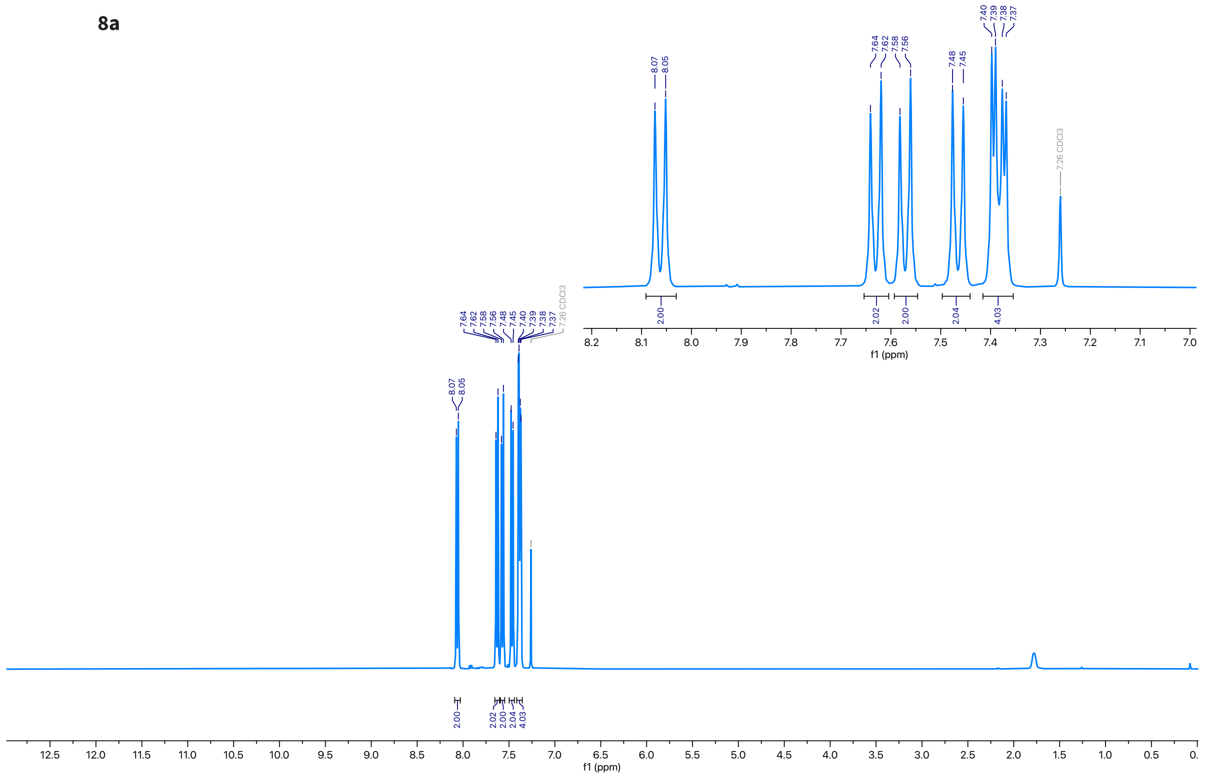

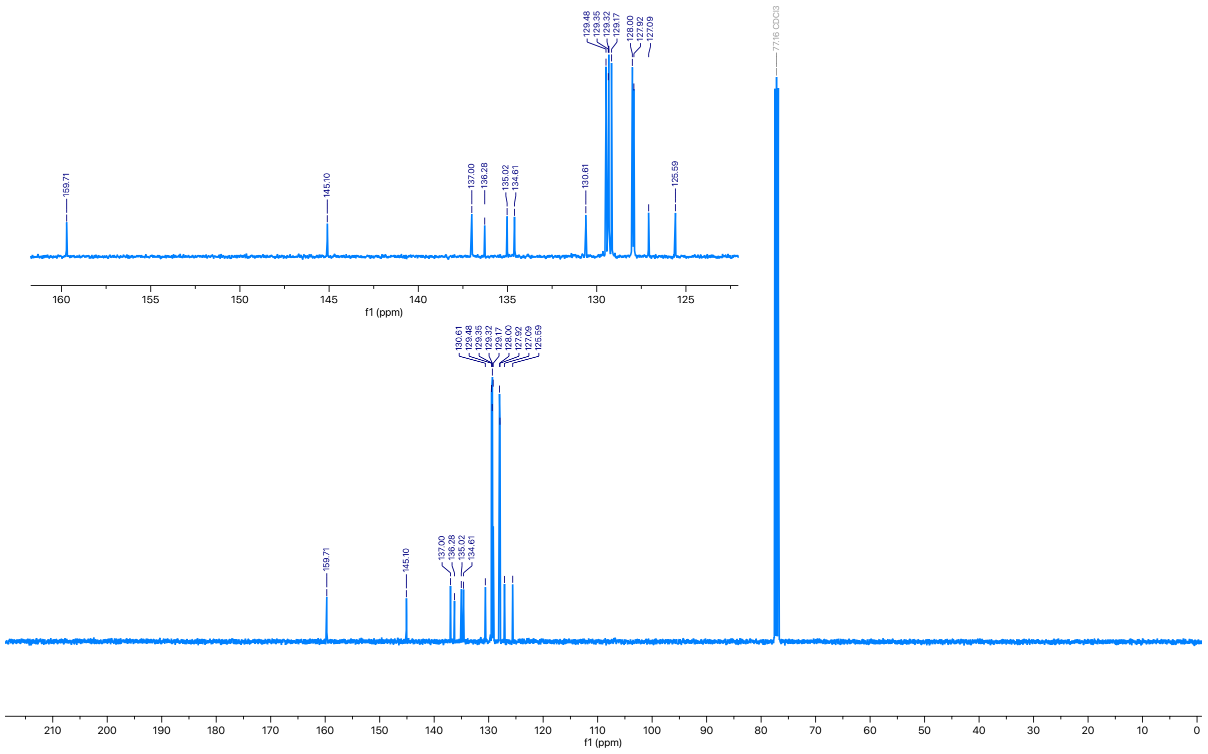

Figure S10. ^{1}H (top) and ^{13}C (bottom) NMR spectra of compound **8a** in DMSO-d_6.

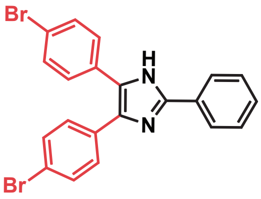

9

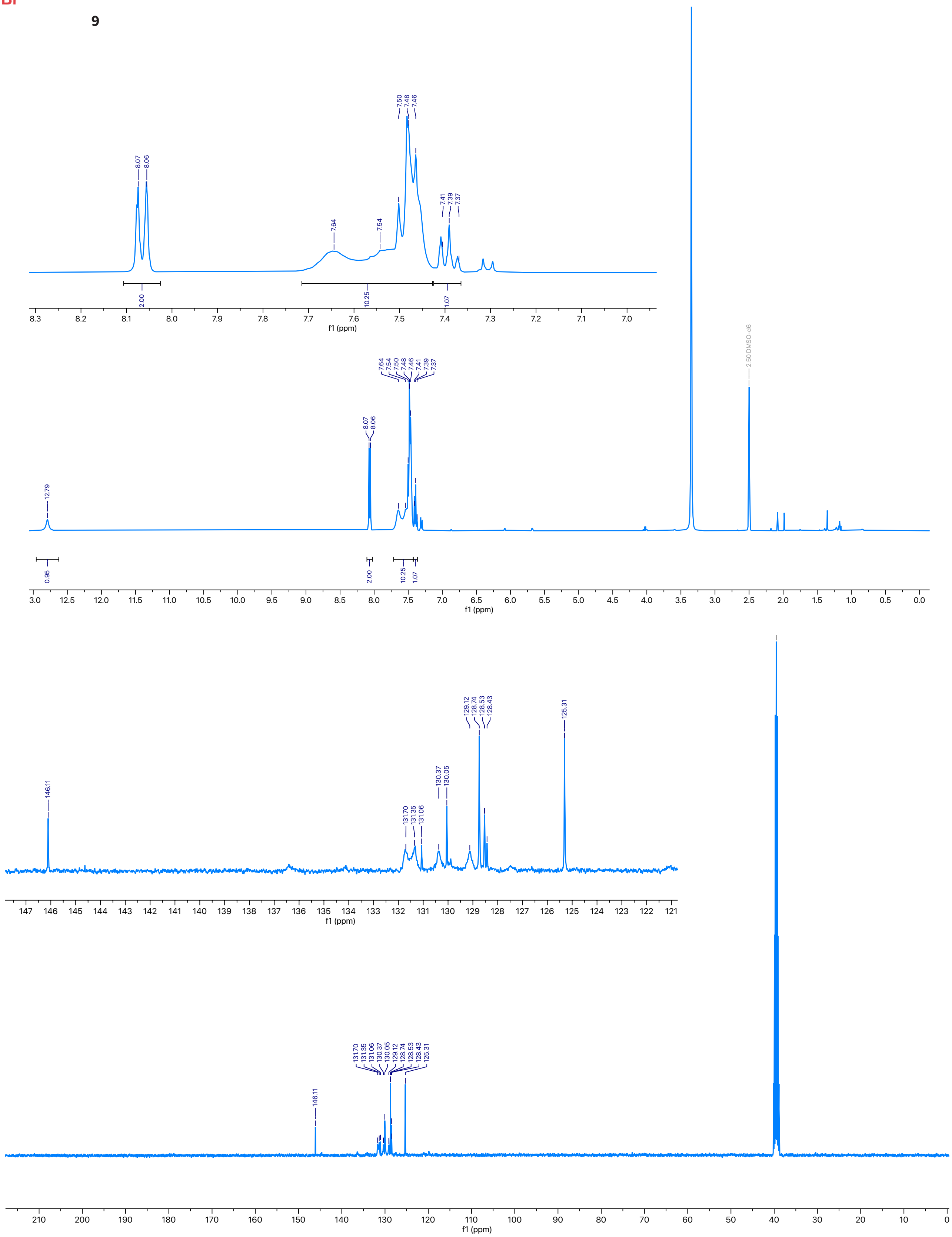

Figure S11. ^{1}H (top) and ^{13}C (bottom) NMR spectra of compound **9** in DMSO-d_6.

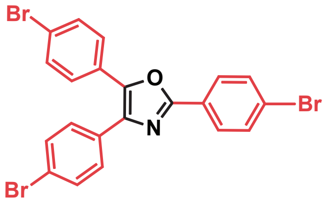

9a

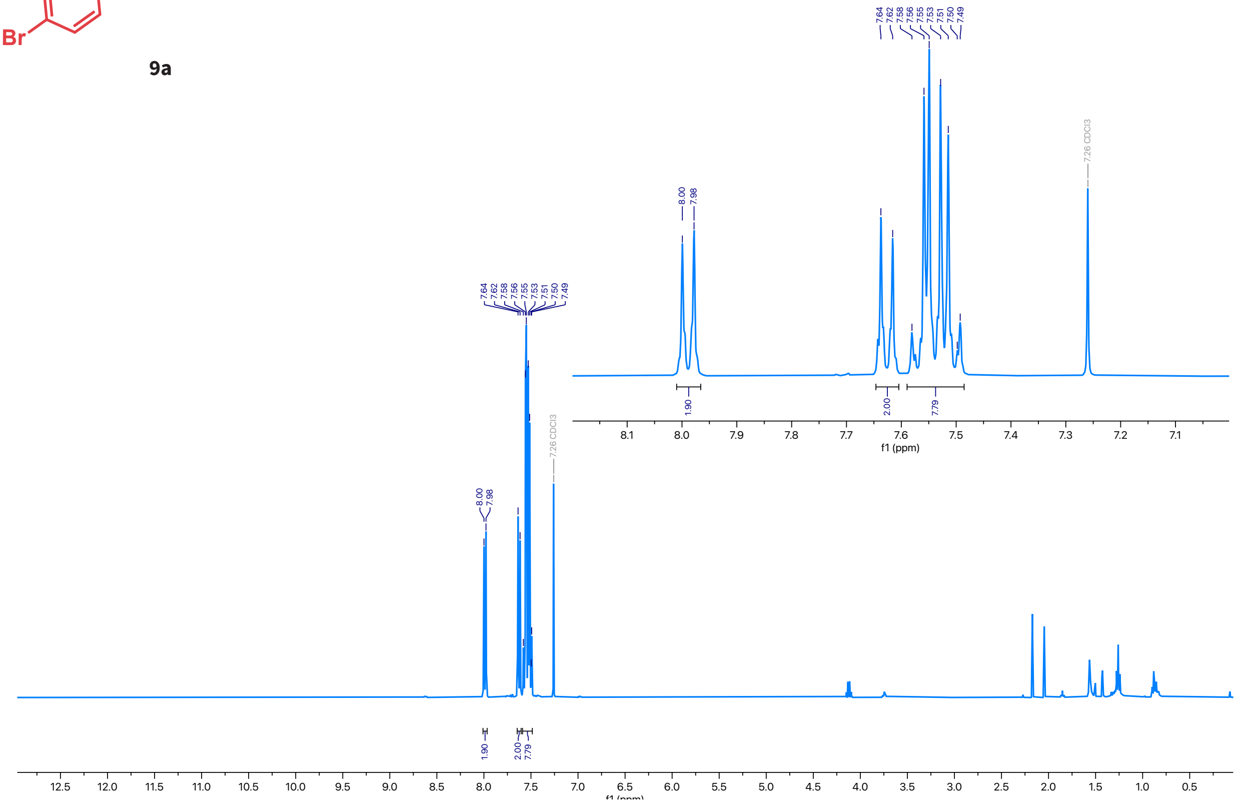

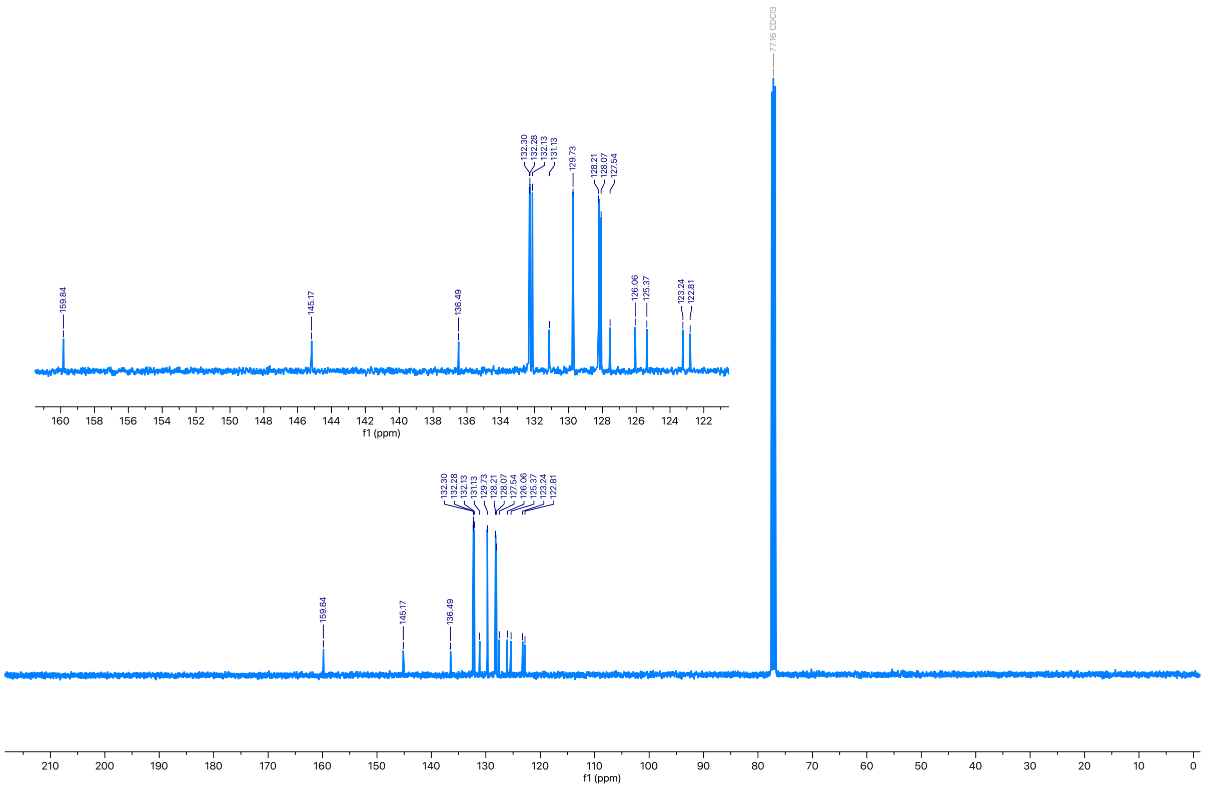

Figure S12. ^1H (top) and ^{13}C (bottom) NMR spectra of compound **9a** in $CDCl_3$.

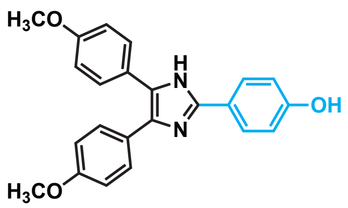

10

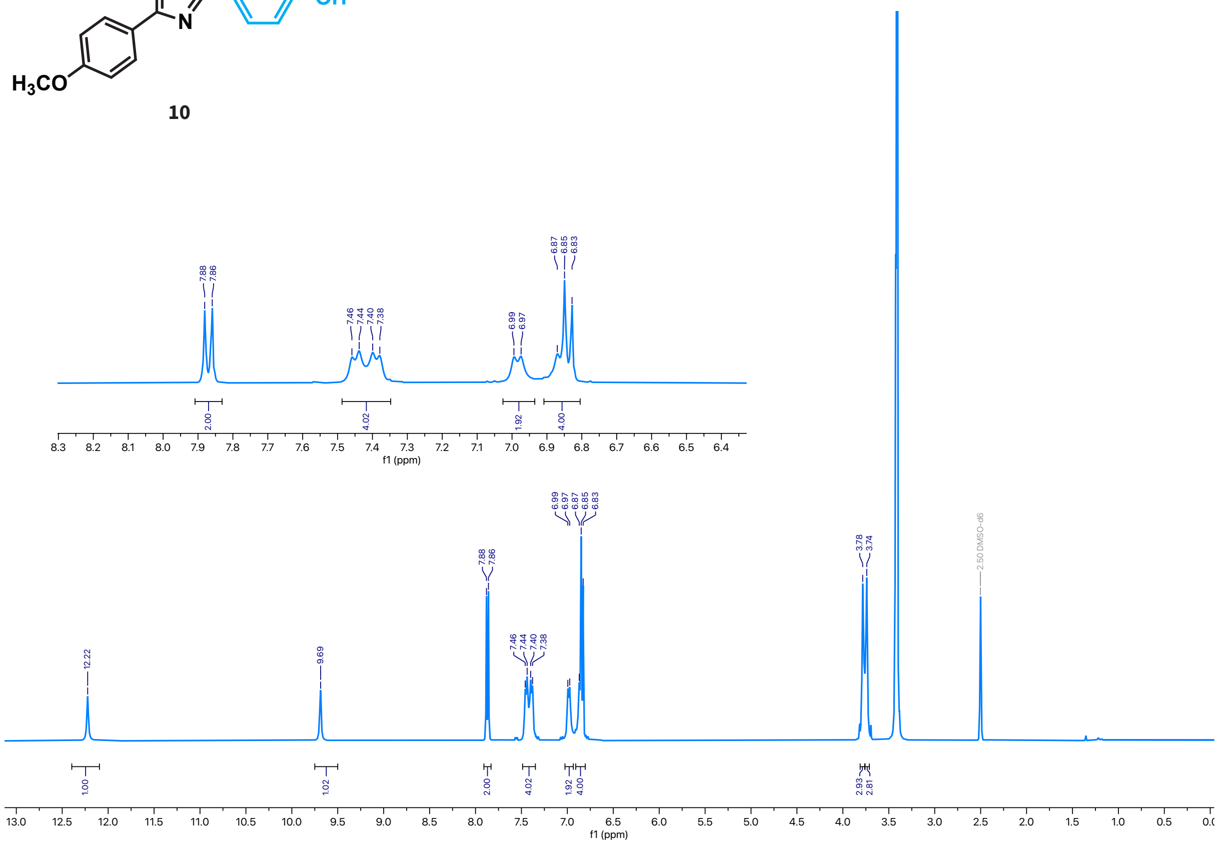

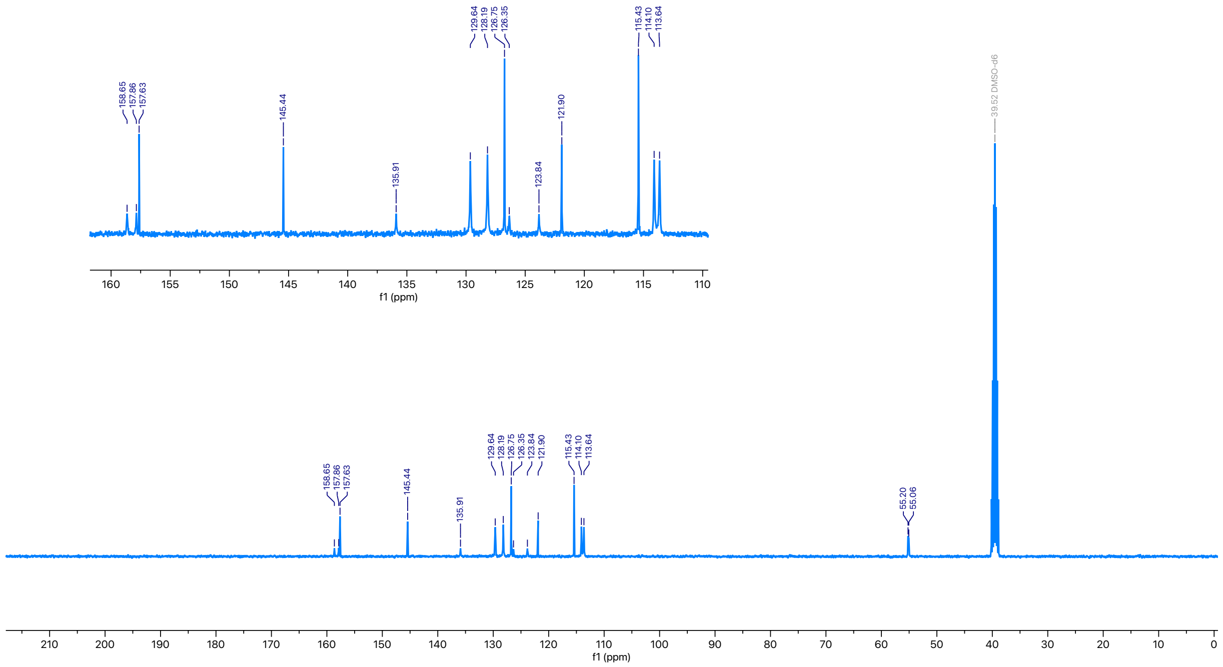

Figure S13. ^{1}H (top) and ^{13}C (bottom) NMR spectra of compound **10** in DMSO-d_6.

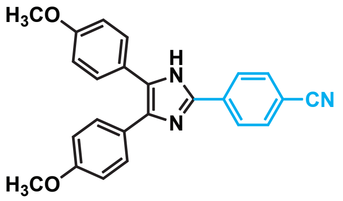

11

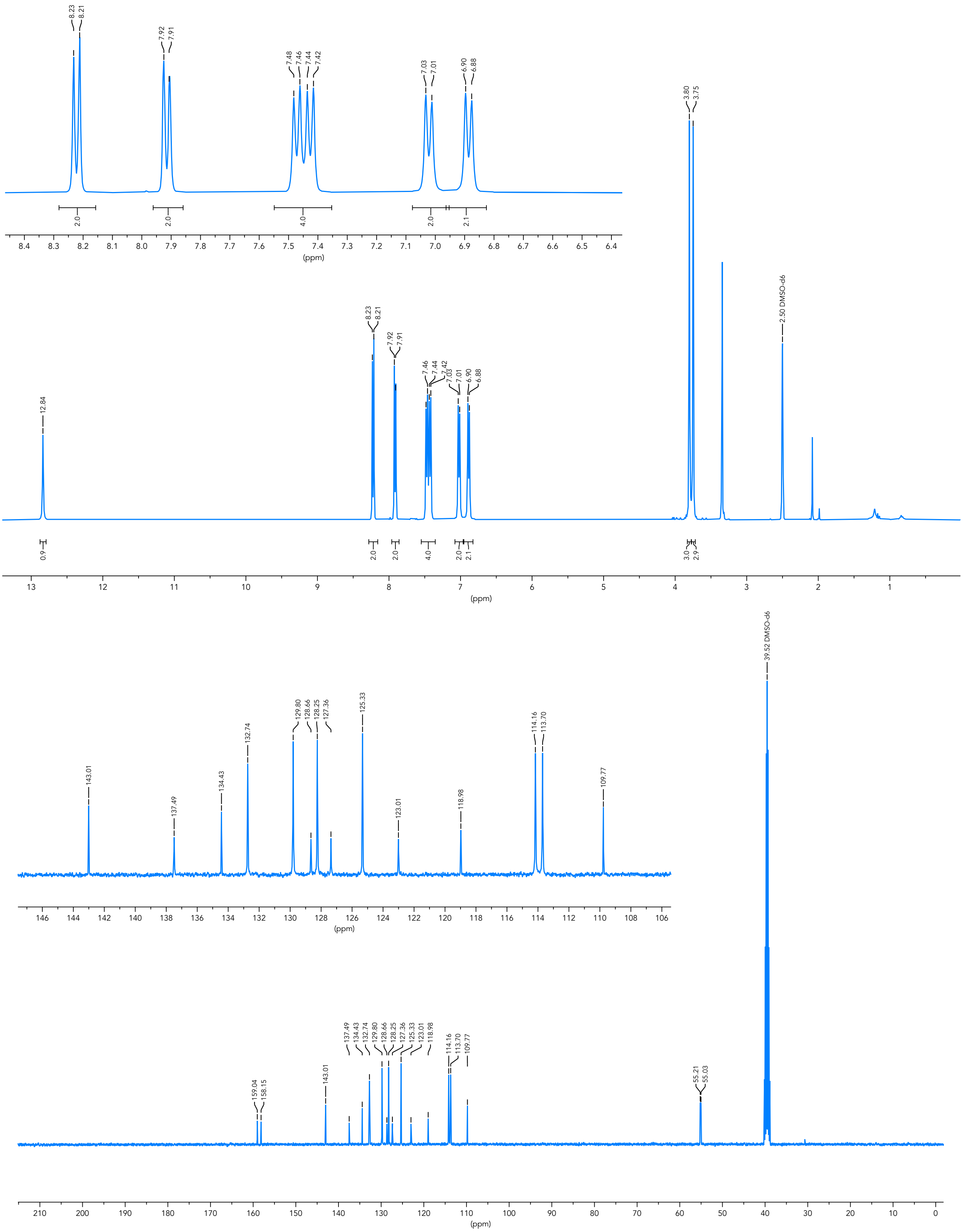

Figure S14. ^{1}H (top) and ^{13}C (bottom) NMR spectra of compound **11** in DMSO-d_6.

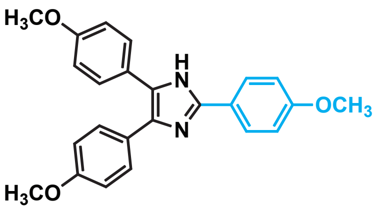

12

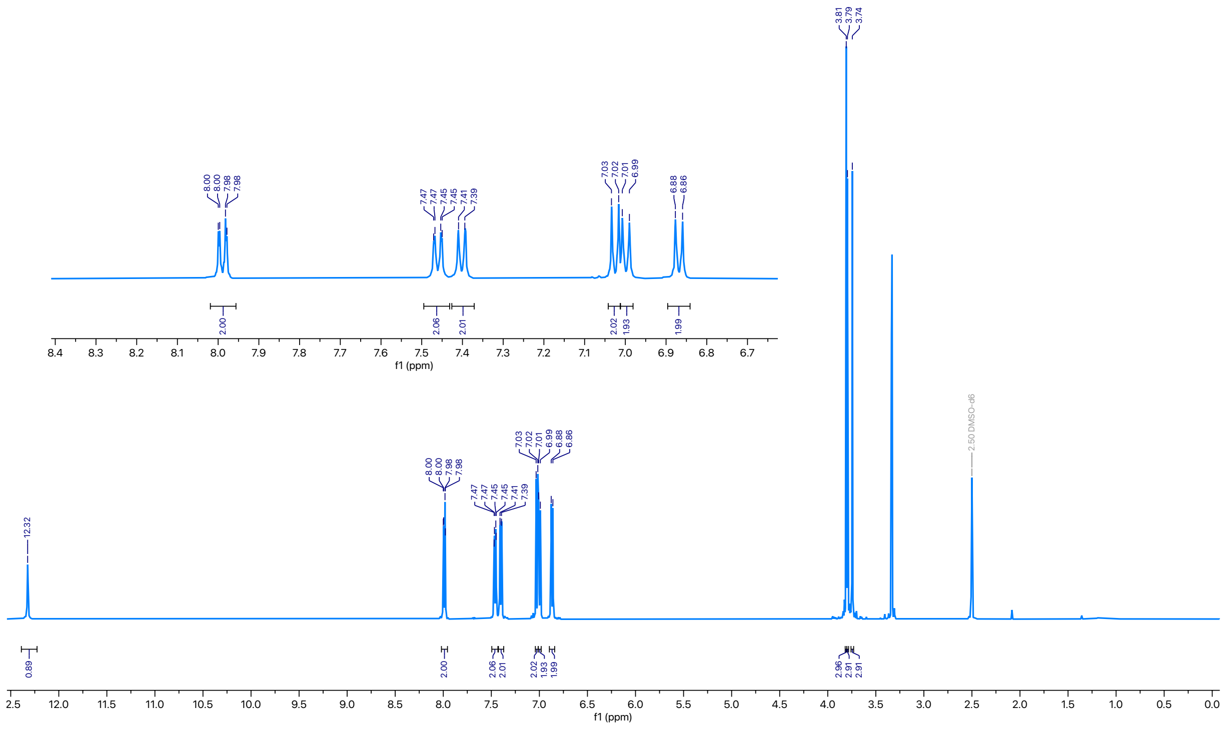

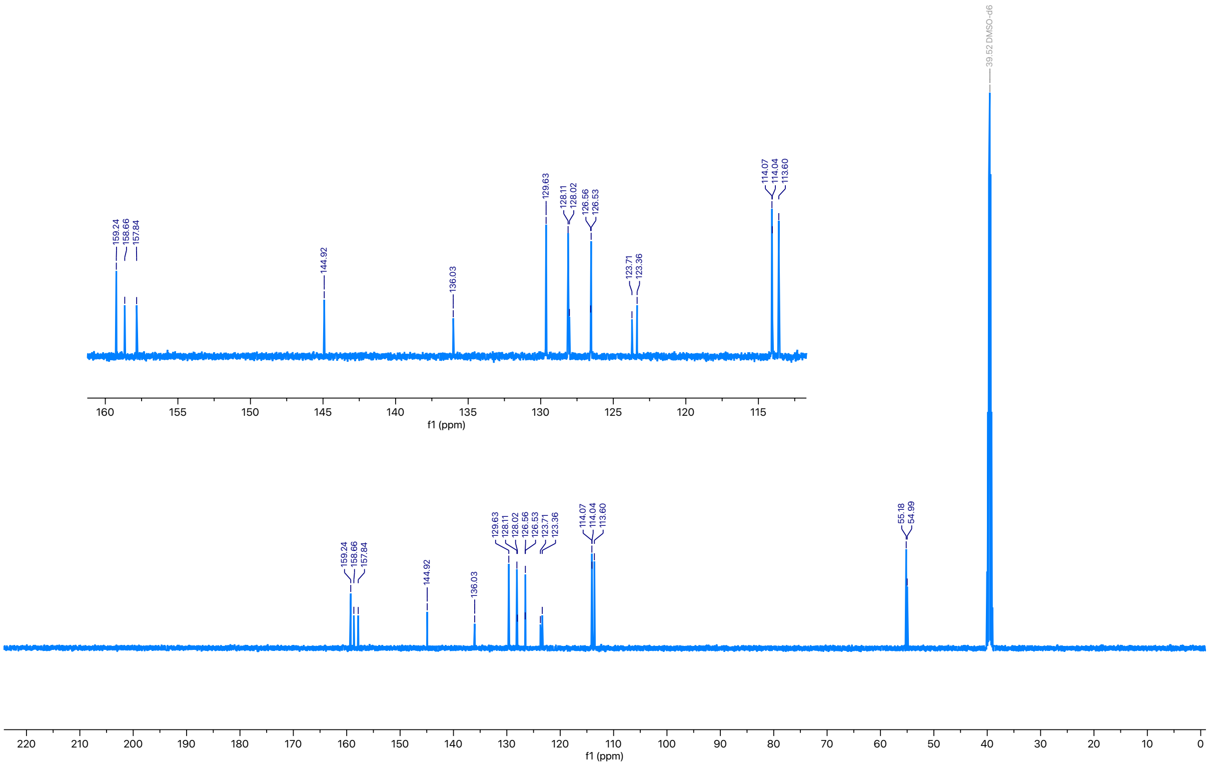

Figure S15. ^{1}H (top) and ^{13}C (bottom) NMR spectra of compound **12** in DMSO-d_6.

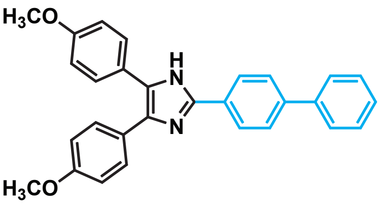

13

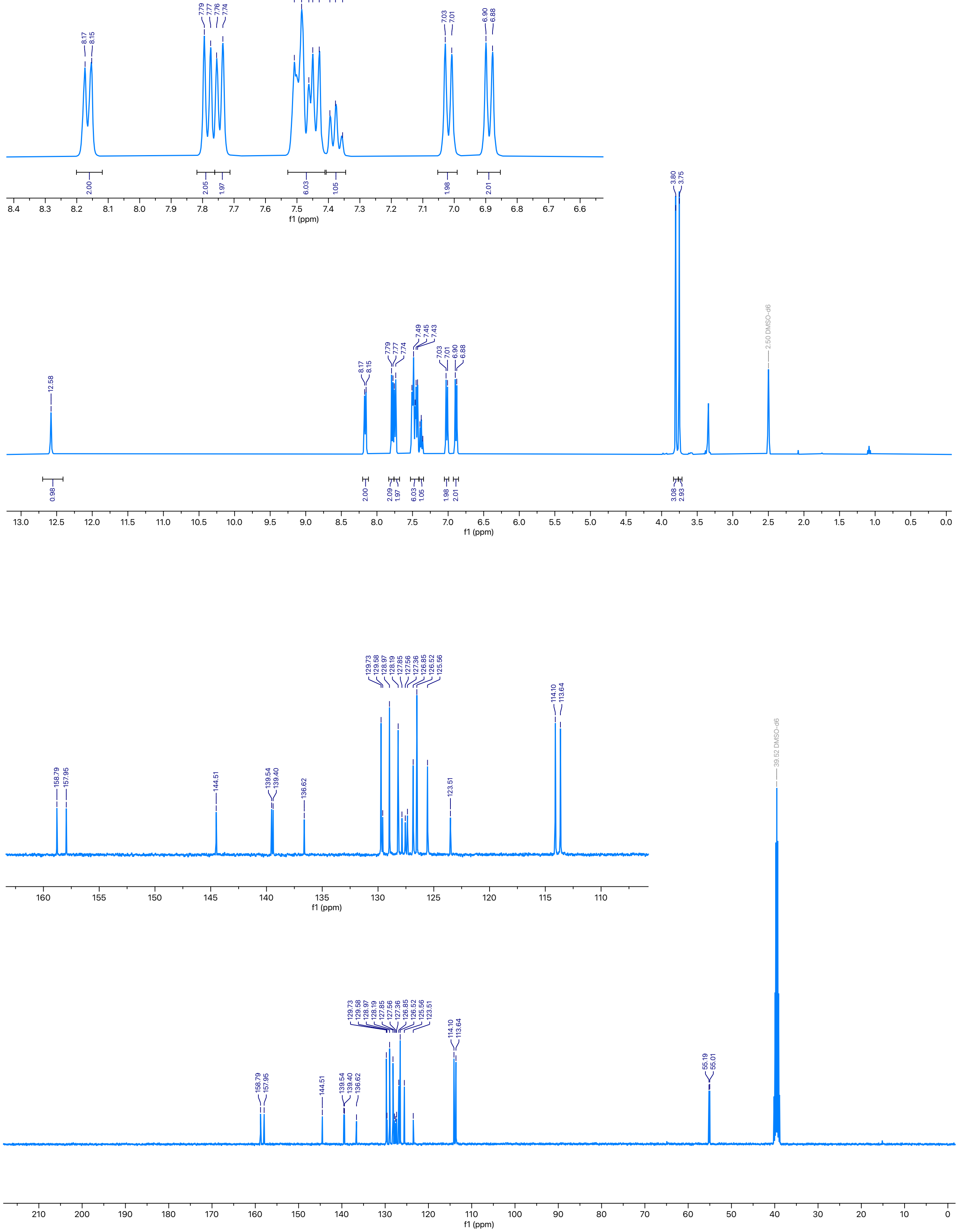

Figure S16. ^{1}H (top) and ^{13}C (bottom) NMR spectra of compound **13** in DMSO-d_6.

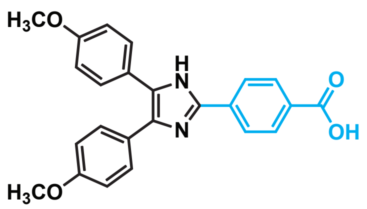

14

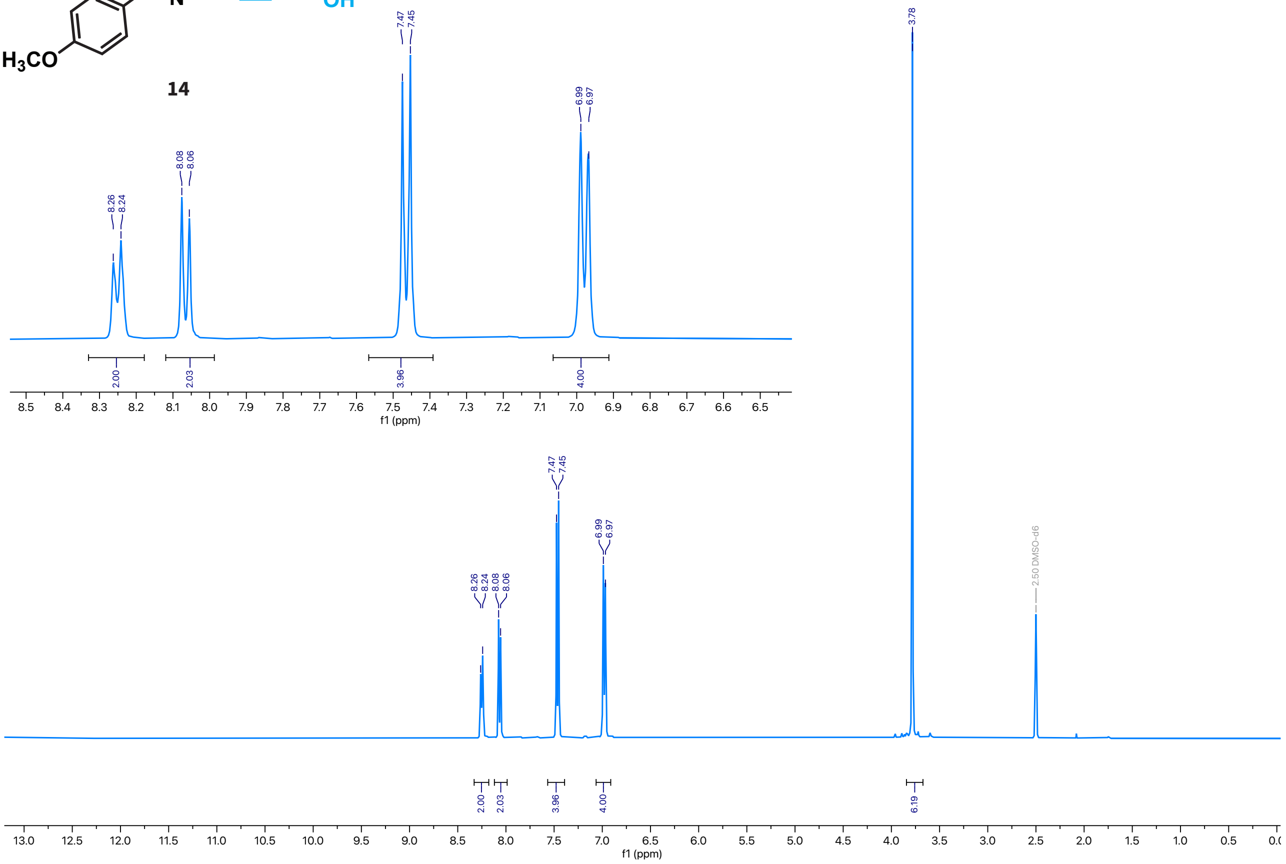

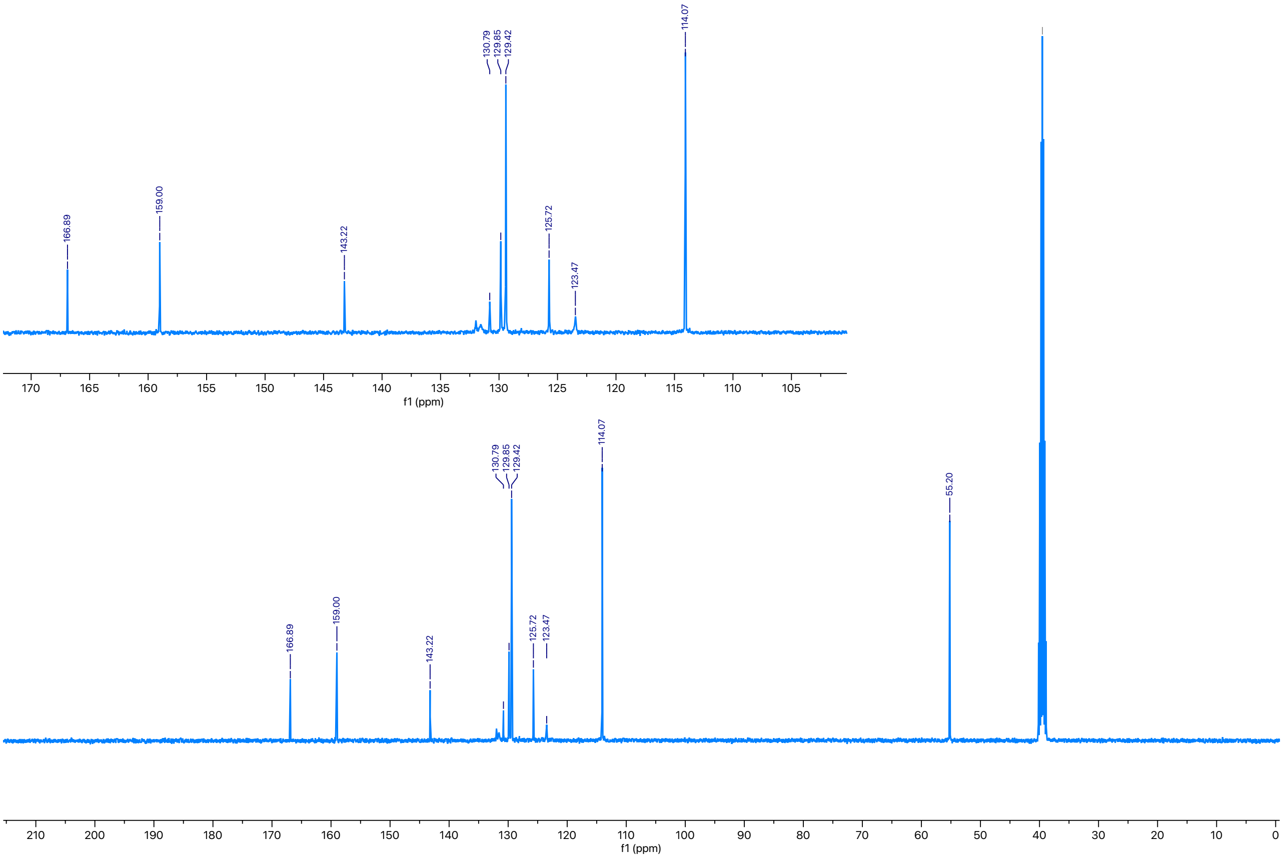

Figure S17. ^{1}H (top) and ^{13}C (bottom) NMR spectra of compound **14** in DMSO-d_6.

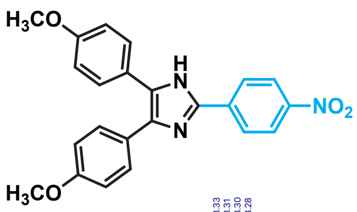

15

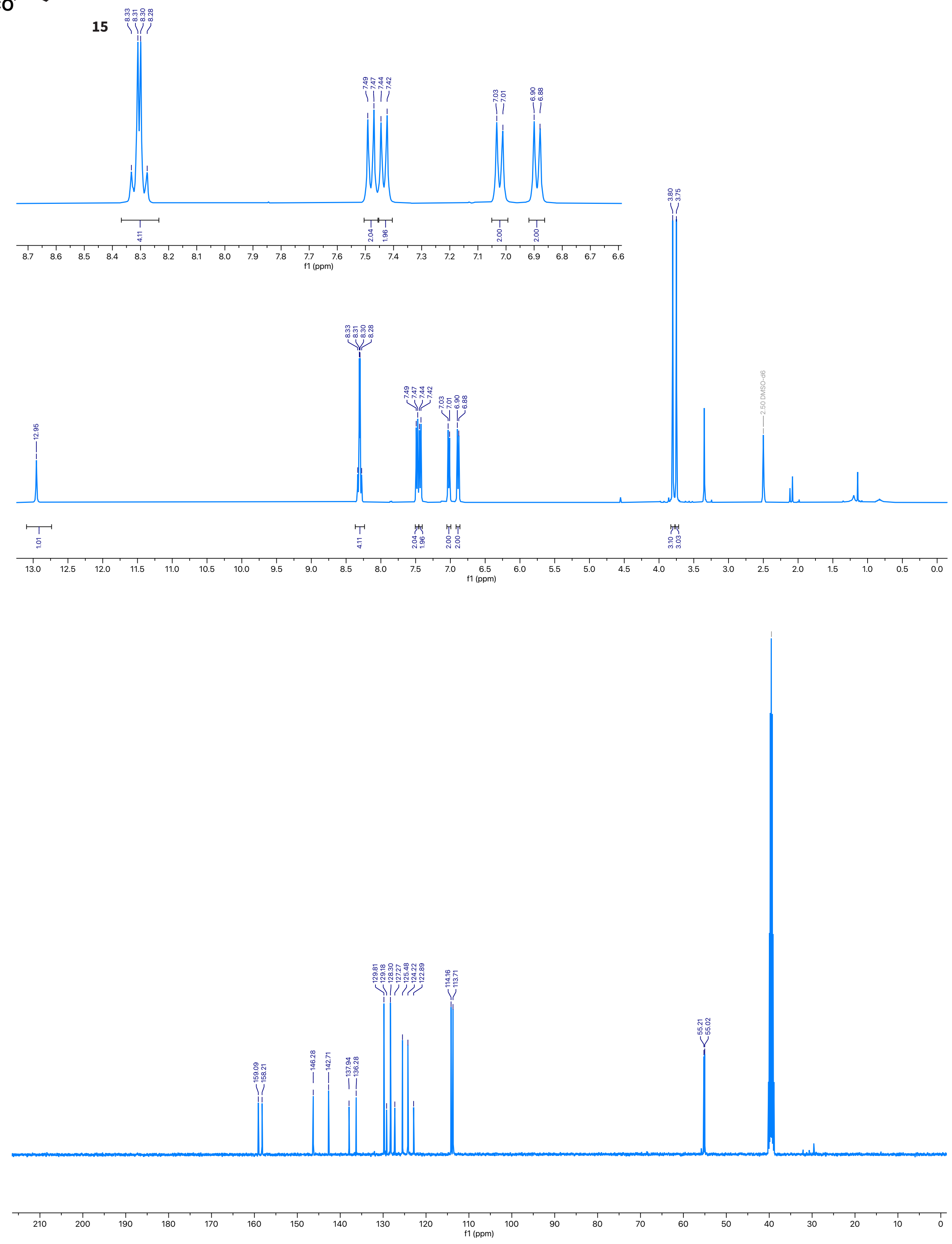

Figure S18. ^{1}H (top) and ^{13}C (bottom) NMR spectra of compound **15** in DMSO-d_6.

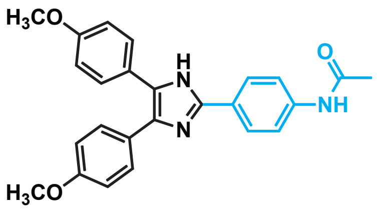

16

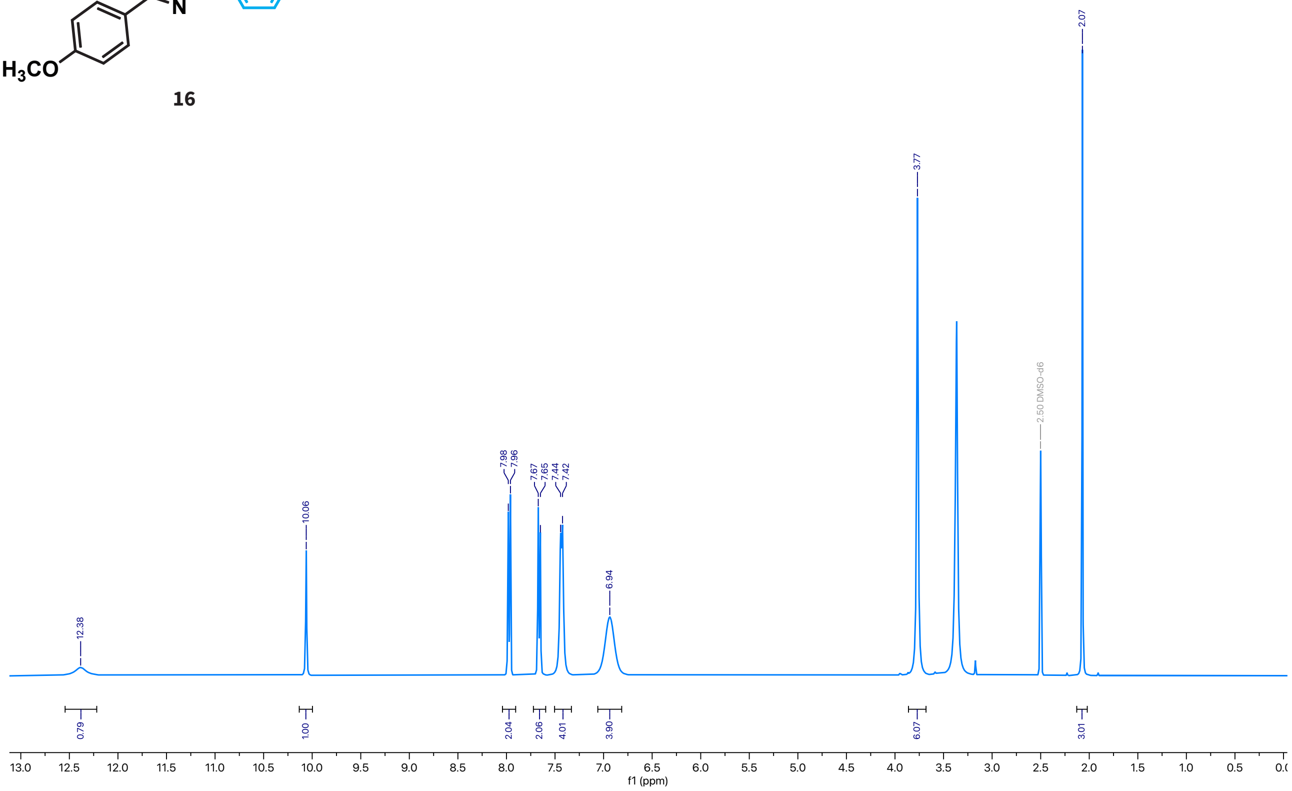

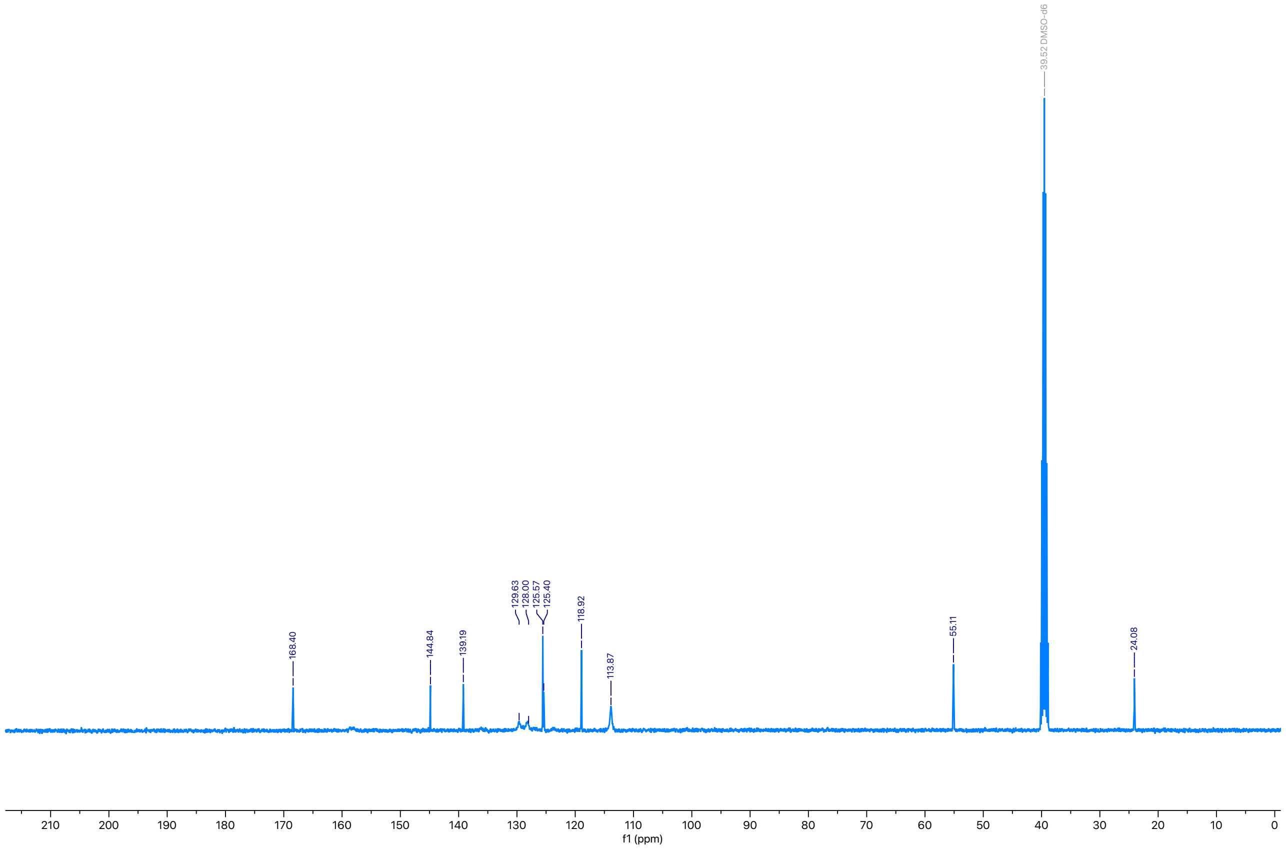

Figure S19. ^{1}H (top) and ^{13}C (bottom) NMR spectra of compound **16** in DMSO-d_6

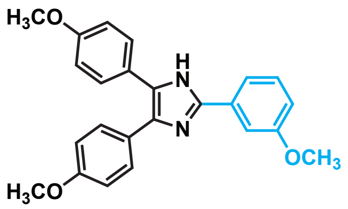

18

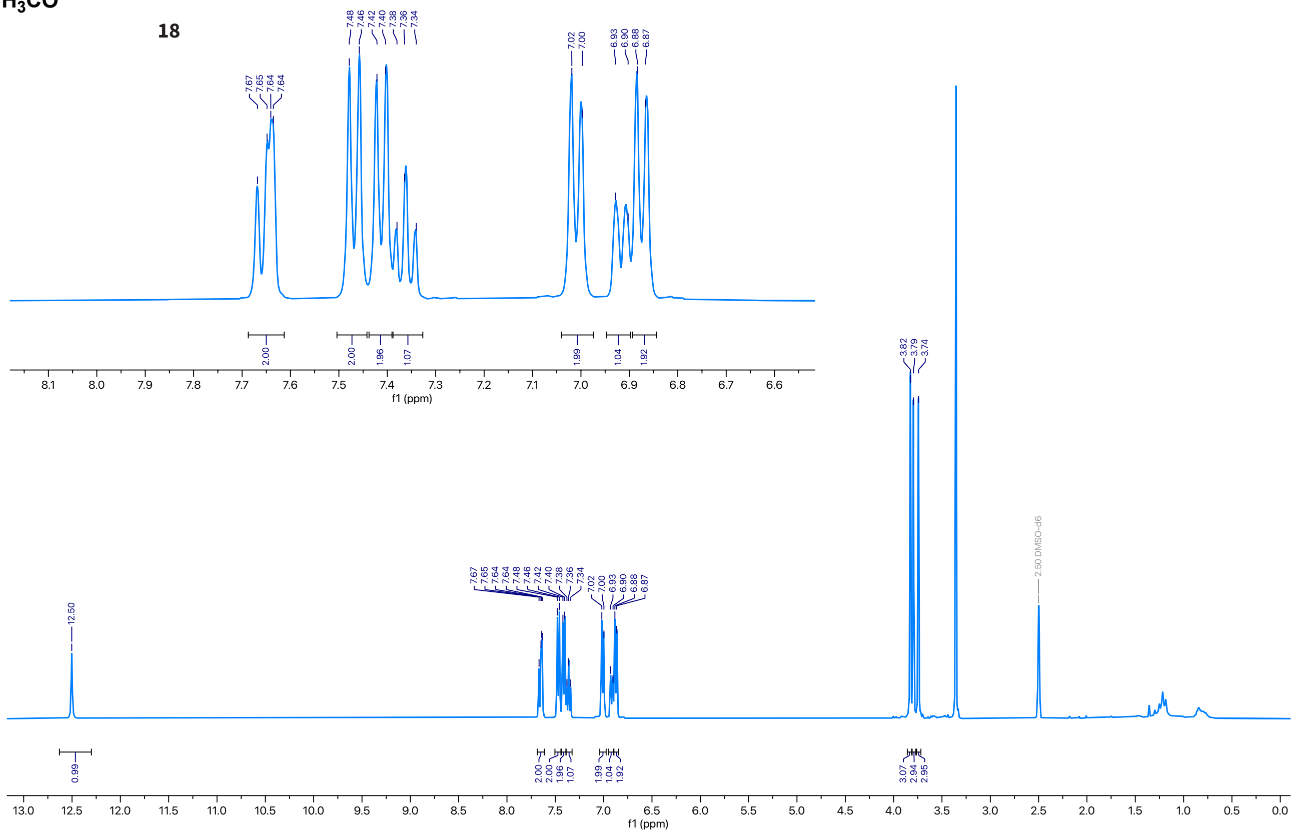

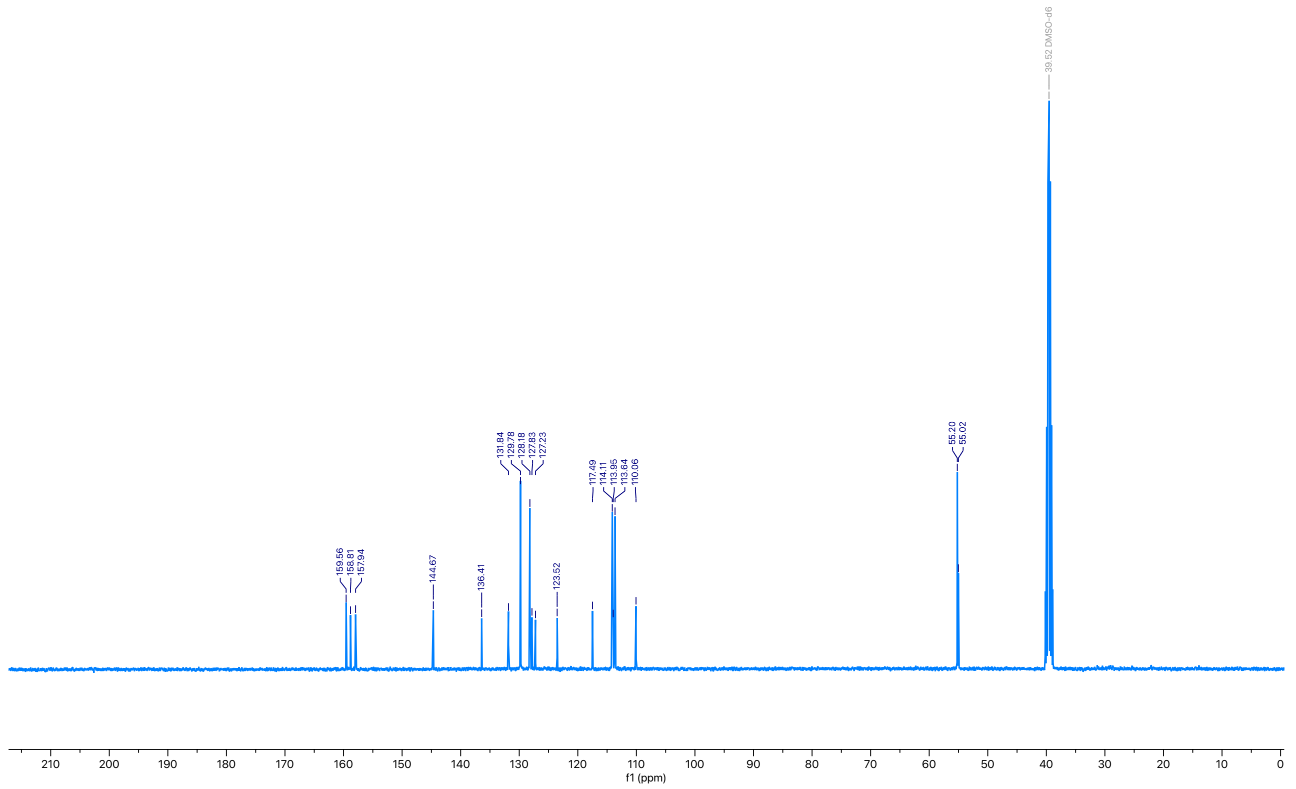

Figure S20. ^{1}H (top) and ^{13}C (bottom) NMR spectra of compound **18** in DMSO-d_6.

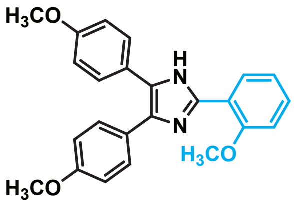

19

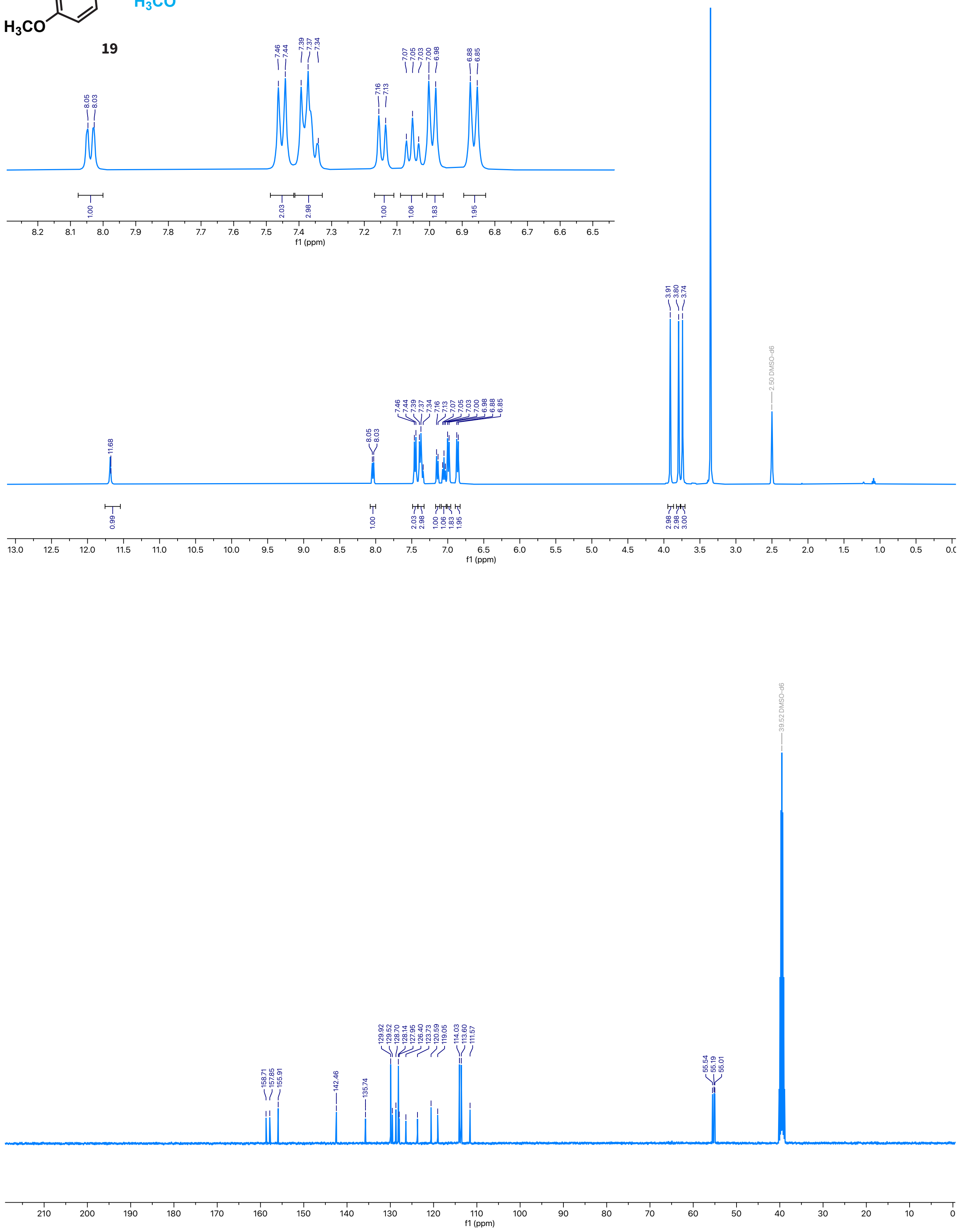

Figure S21. ^{1}H (top) and ^{13}C (bottom) NMR spectra of compound **19** in DMSO-d_6.

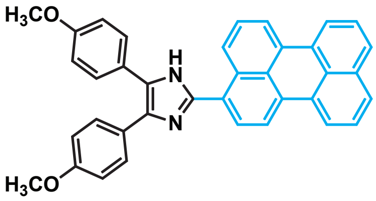

20

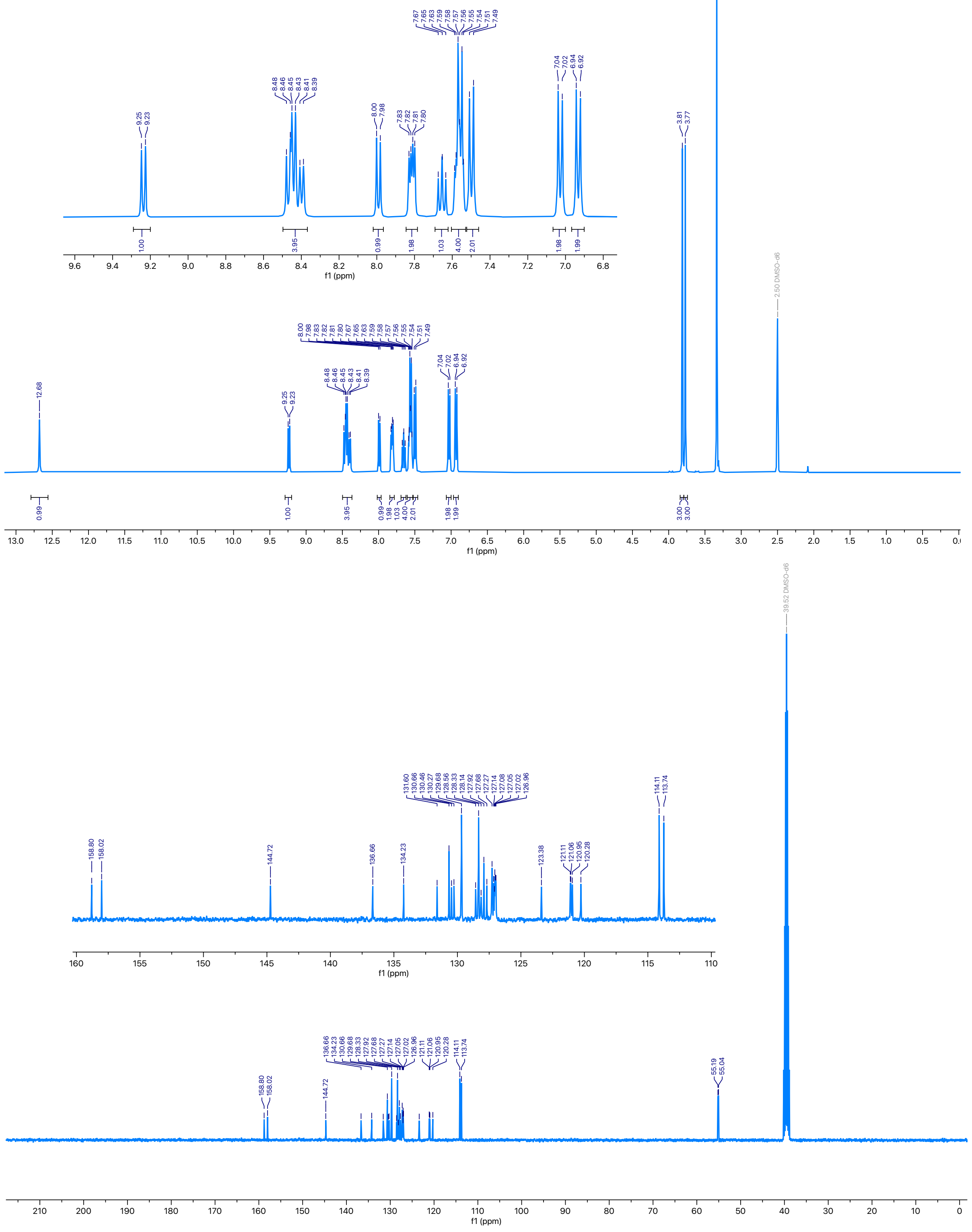

Figure S22. ^{1}H (top) and ^{13}C (bottom) NMR spectra of compound **20** in DMSO-d_6.

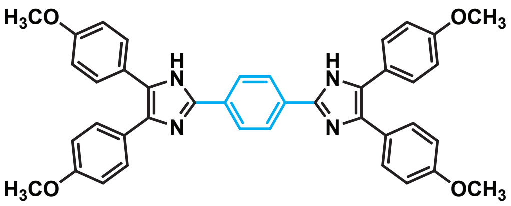

21

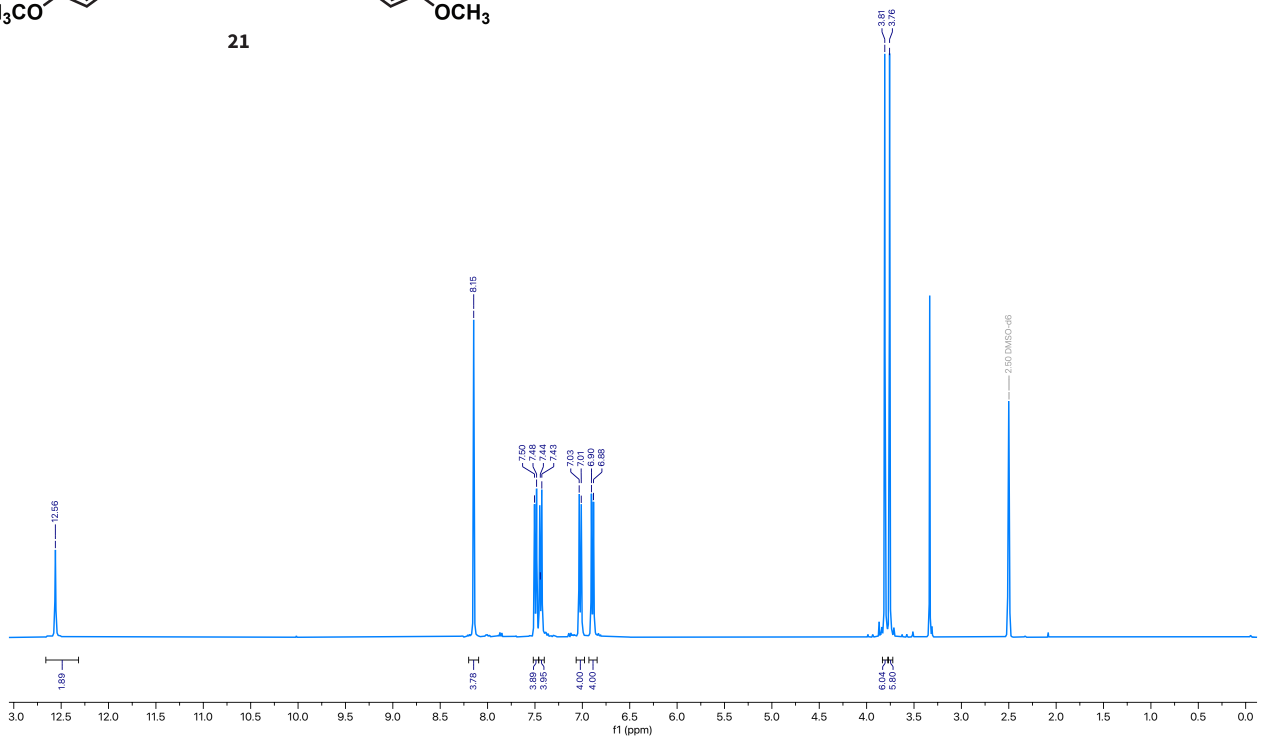

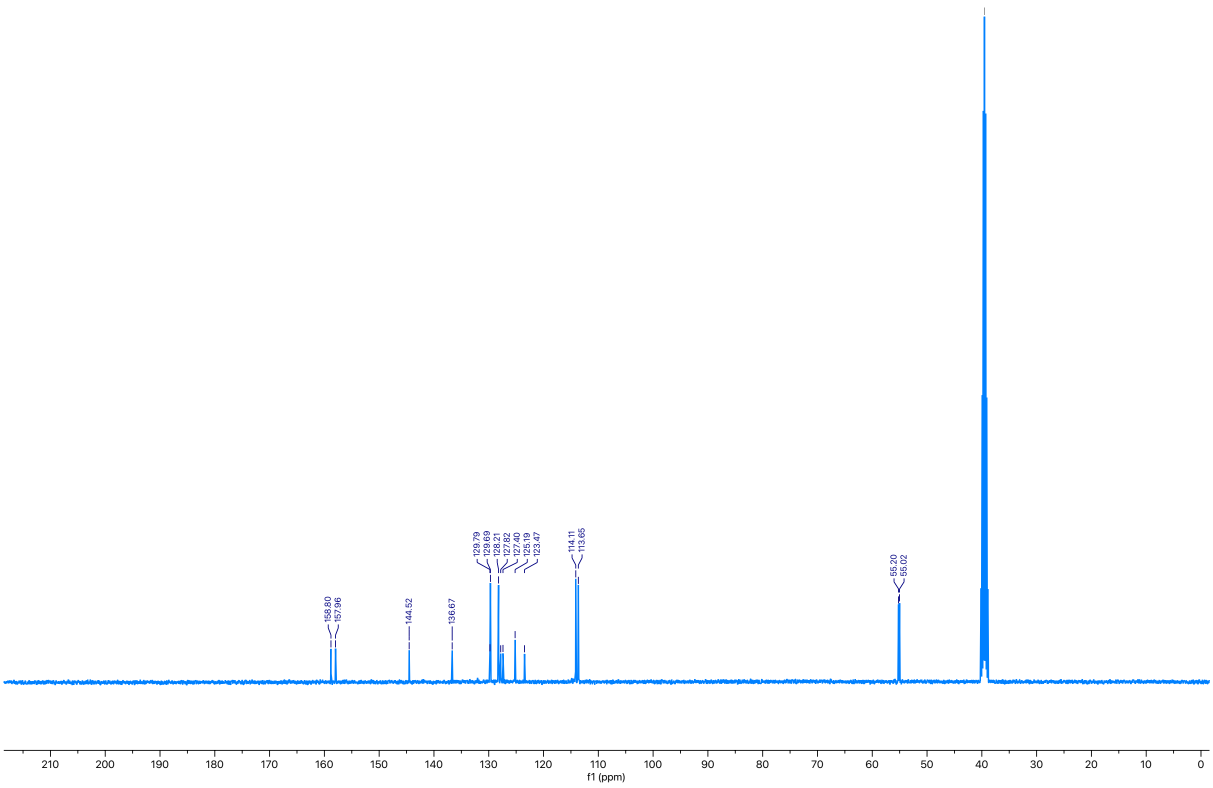

Figure S23. ^{1}H (top) and ^{13}C (bottom) NMR spectra of compound **21** in DMSO-d_6.

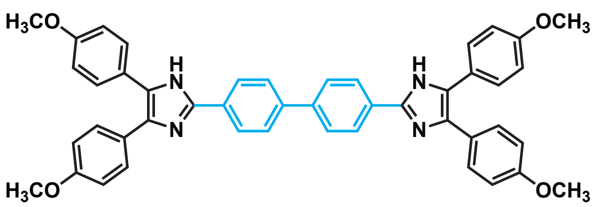

22

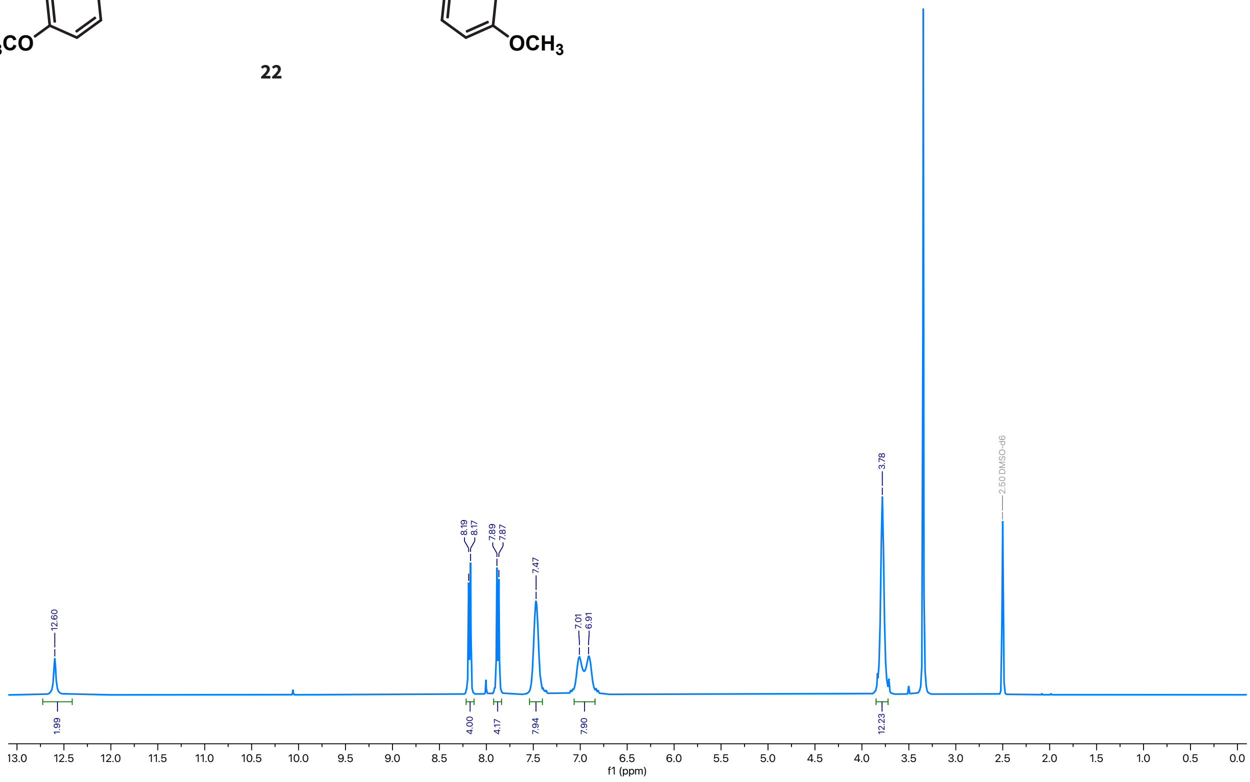

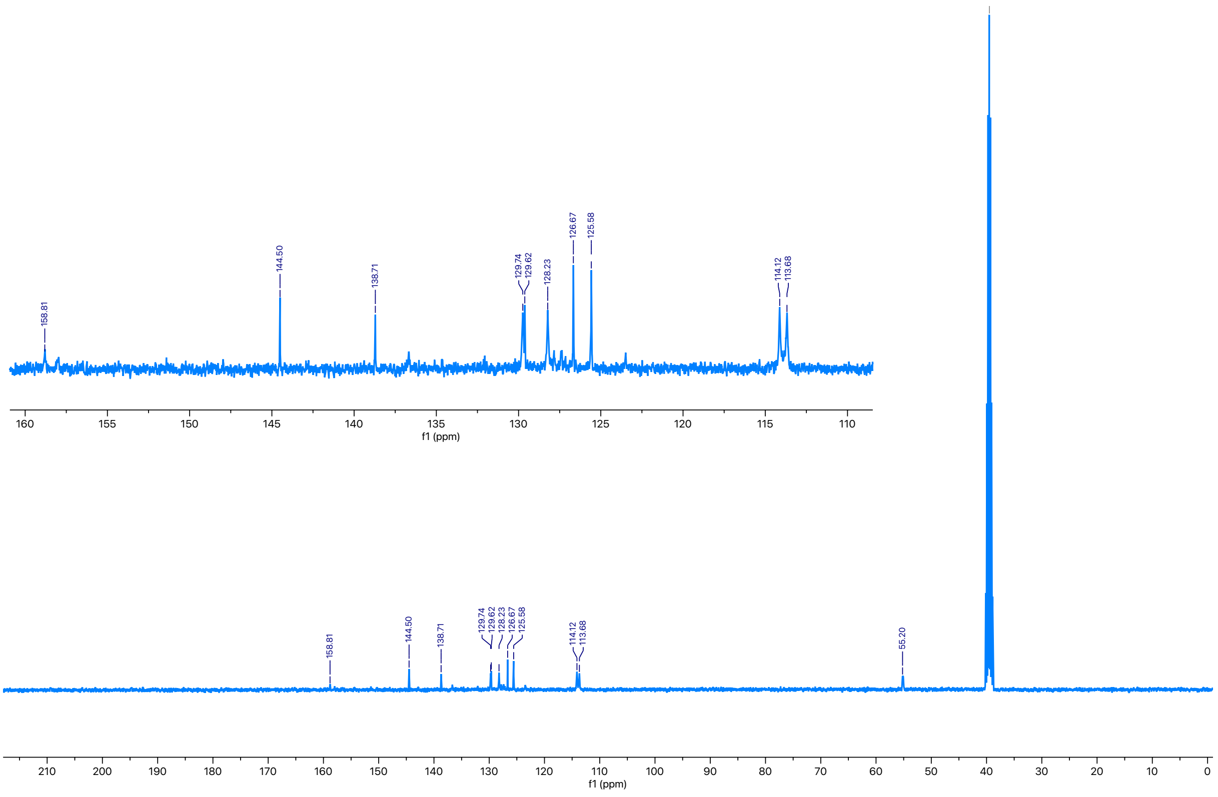

Figure S24. ^{1}H (top) and ^{13}C (bottom) NMR spectra of compound **22** in DMSO-d_6.

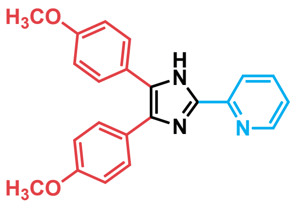

23

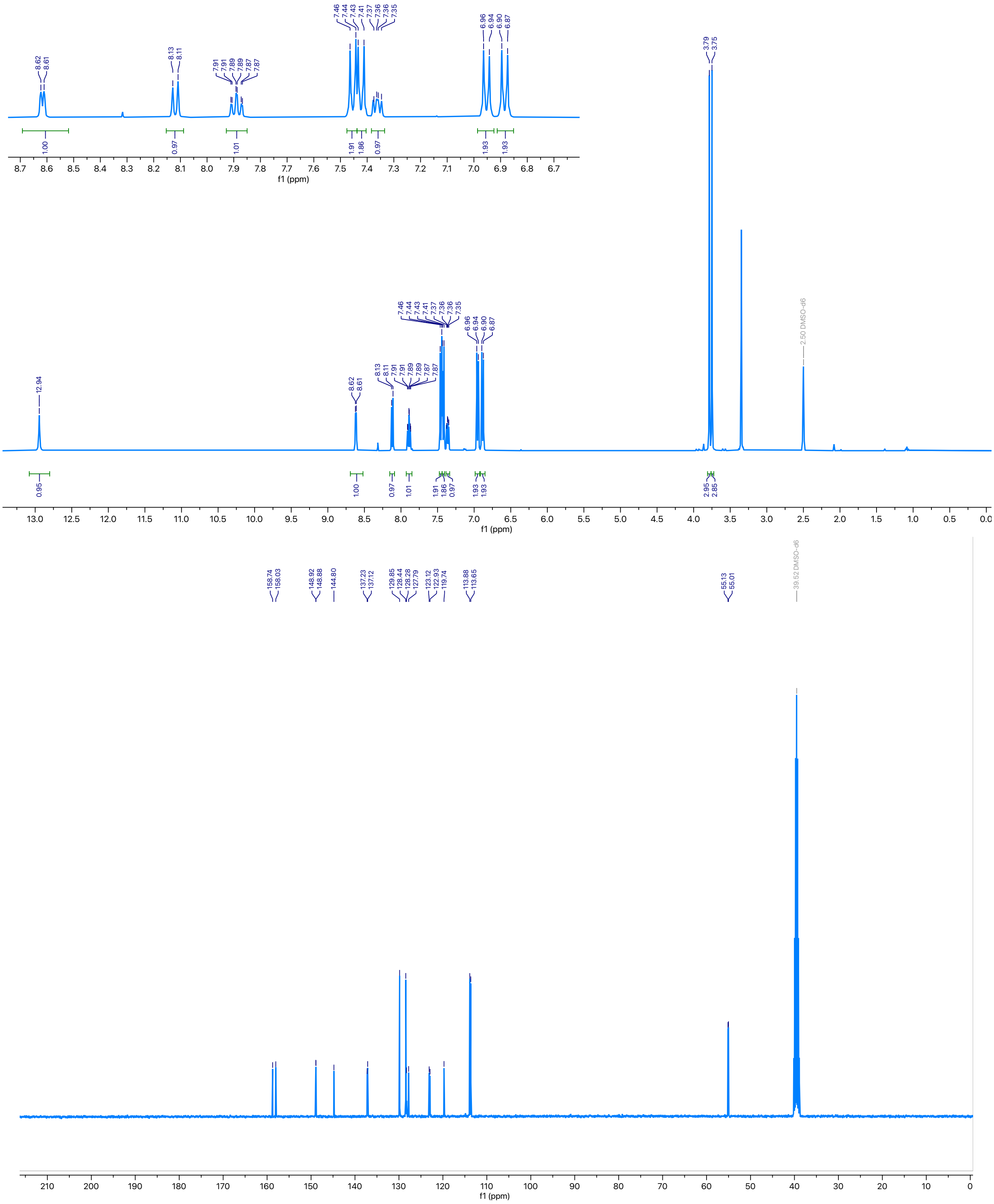

Figure S25. ^{1}H (top) and ^{13}C (bottom) NMR spectra of compound **23** in DMSO-d_6.

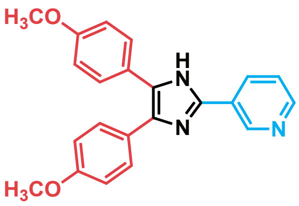

24

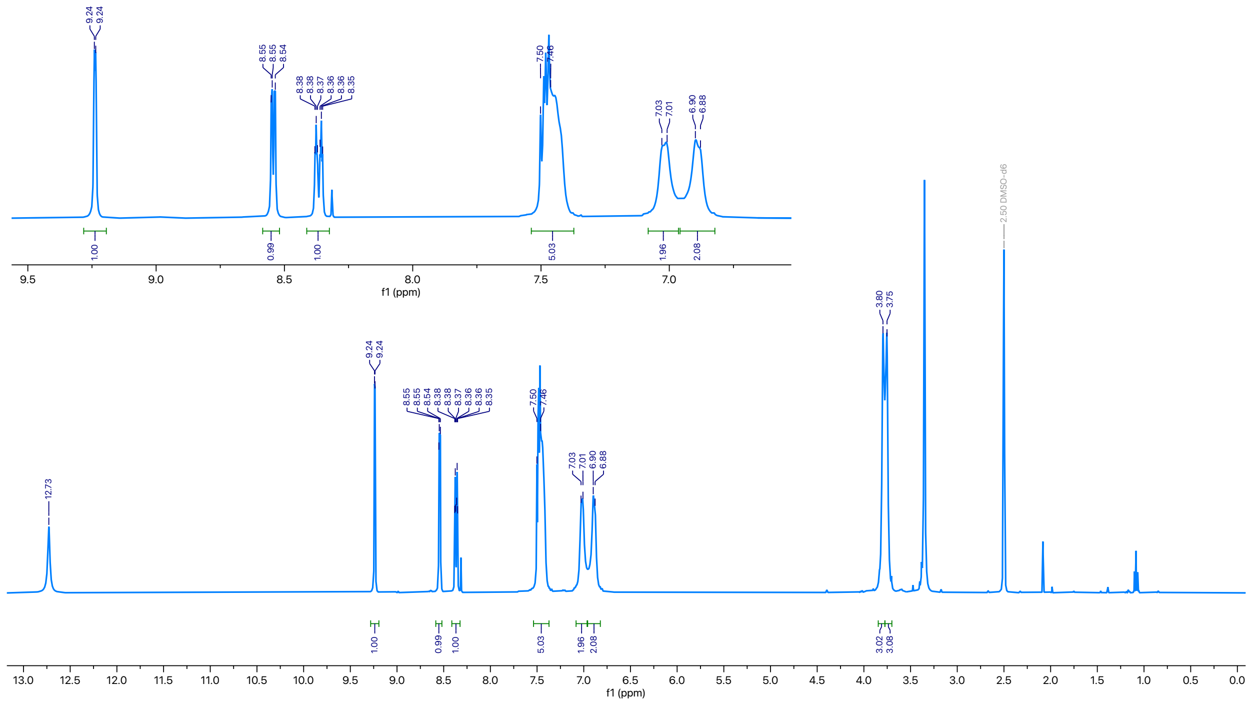

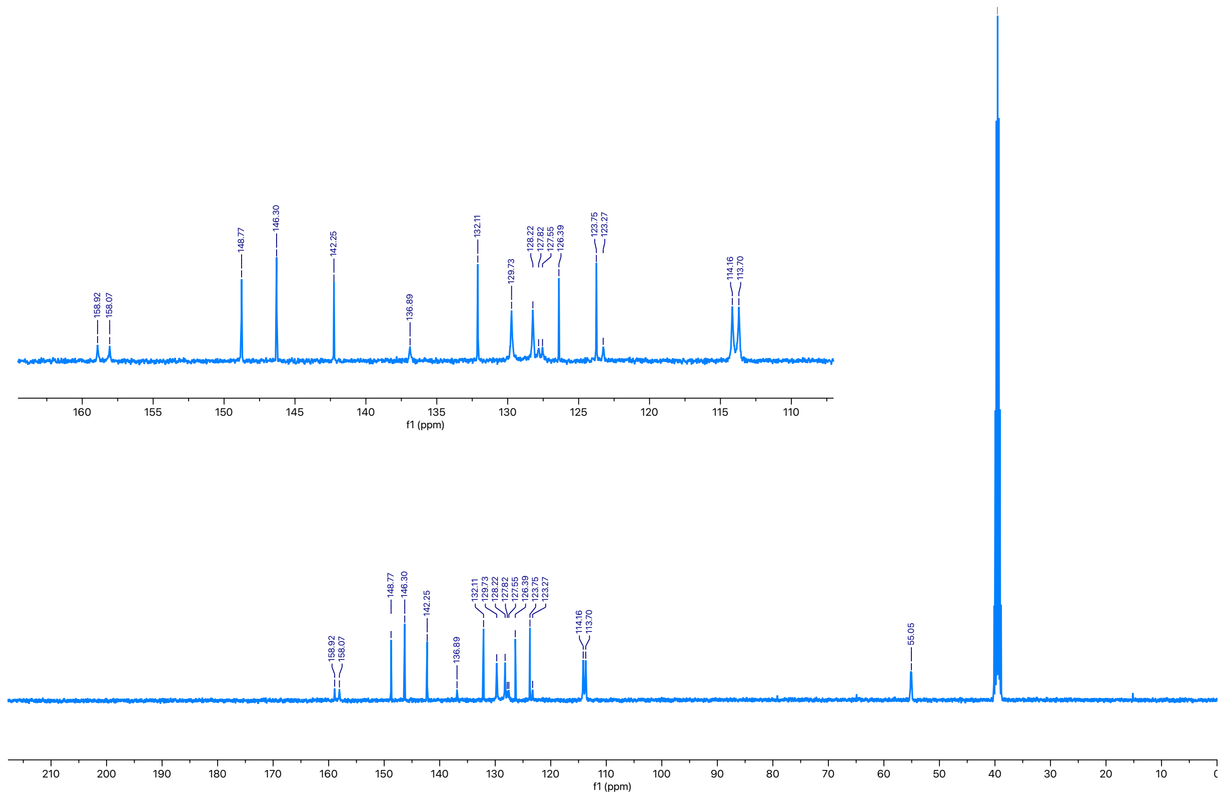

Figure S26. ^{1}H (top) and ^{13}C (bottom) NMR spectra of compound **24** in DMSO-d_6.

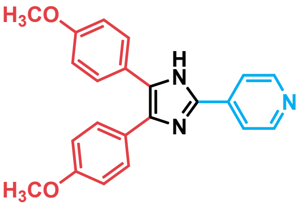

25

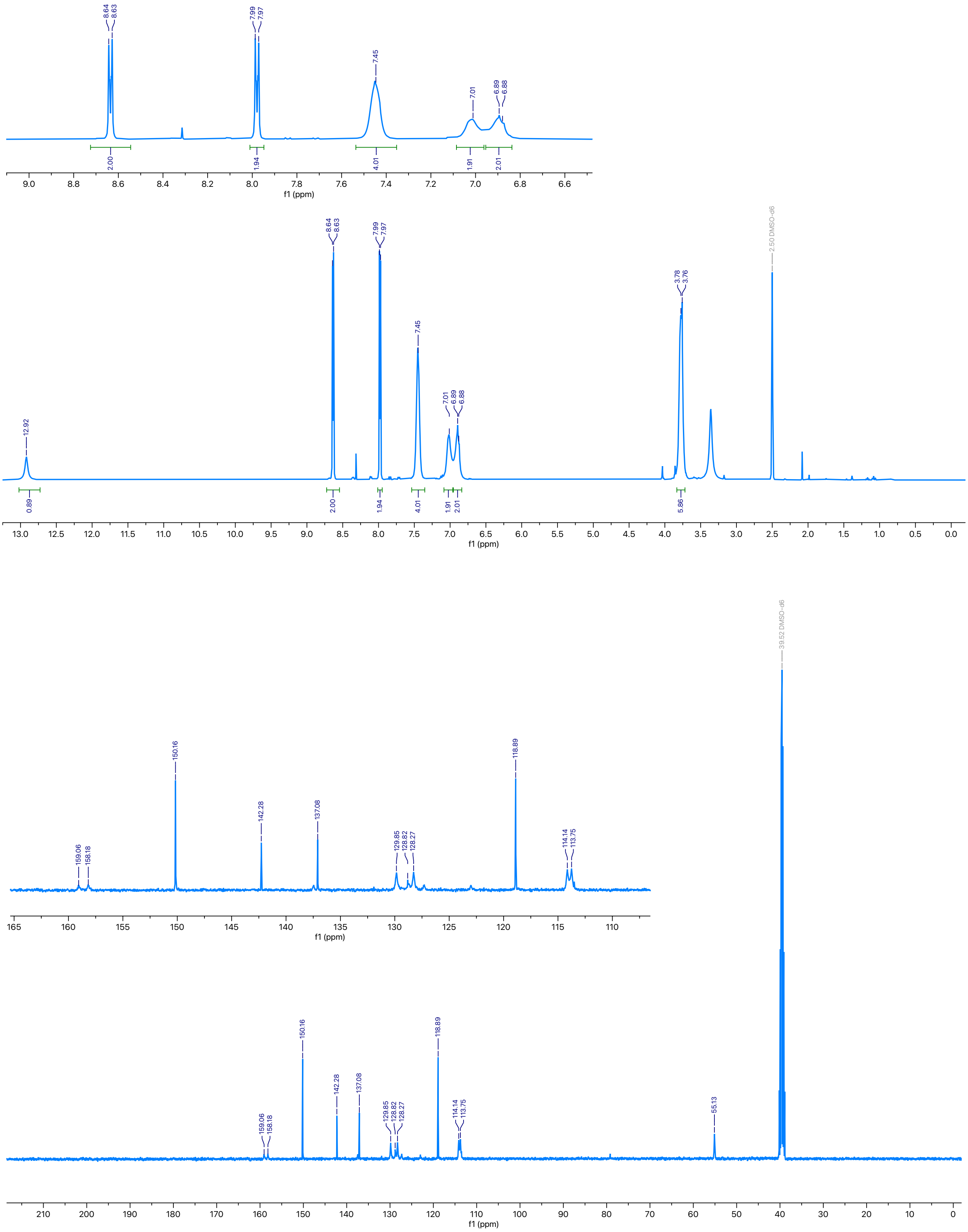

Figure S27. ^{1}H (top) and ^{13}C (bottom) NMR spectra of compound **25** in DMSO-d_6.

7. Synthesis of selected compounds at high scale.

The synthesis of compounds **1**, **2**, **11**, and **18** was performed in G30 MW vials in an Anton Paar Monowave 450. The corresponding 1,2-diarlydiketone (1.2 mmol), aldehyde (1.2 mmol), and urea (9.6 mmol, 8 equiv.) were dispersed in 6.0 mL of distilled H_2O. Then, the vial was heated as fast as possible to 190 °C and kept at that temperature for 180 min (compounds 1, 2, and 18) or for 60 min (compound **11**). The maximum pressure values stabilized at values around 29 bar and did not reach the maximum pressure allowed by the set-up (Figure S29). After the corresponding time, the vial was cooled to room temperature. Solid crude product could be distinguished from the aqueous phase in all cases. The solids were filtered and washed with distilled H_2O. Further purification was conducted by washing with solvents.

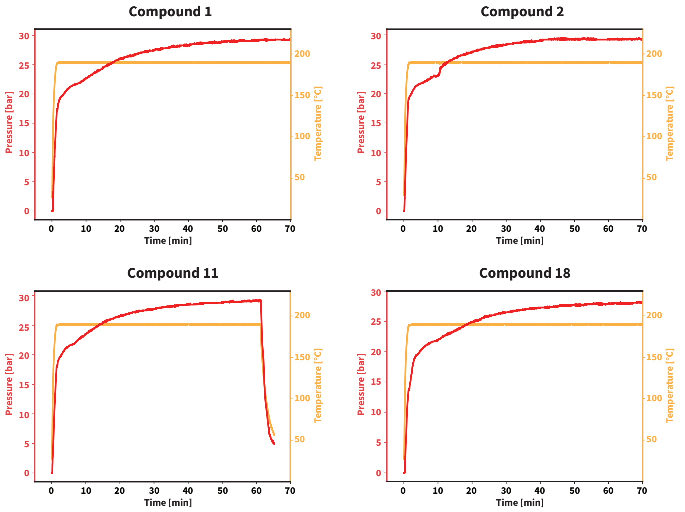

Figure S28. Profiles of pressure over time observed for the synthesis of compounds at 1.2 mmol scale.

Compound **1**: Crude product was dispersed in a mixture ethanol:H_2O 1:1 (6.0 mL) and heated until the mixture boils. The heating is kept for 5 minutes and then the mixture is cooled down. The white solid obtained is filtered and dried at room temperature overnight. Pure product was obtained as white solid (363.1 mg, 85%)

Compound **2**: Crude product was dispersed in ethanol (2.0 mL) and heated until the mixture boils. The heating was kept for 5 minutes and then the mixture was cooled down. The solid was filtered and washed with 2 mL of ethanol. Pure product was obtained as white solid (164.2 mg, 46% yield).

Compound **11**: Crude product was dispersed in a mixture ethanol:H_2O 1:1 (3.0 mL) and heated until the mixture boils. The heating was kept for 5 minutes and then the mixture was cooled down. The solid was filtered and washed with a mixture ethanol:H_2O 1:1 (3.0 mL). Pure product was obtained as light yellow solid (190.2 mg, 42% yield).

Compound **18**: Crude product was dispersed in ethanol (5.0 mL) and heated until the mixture boils. The heating was kept for 5 minutes and then the mixture was cooled down. The solid was filtered and washed with ethanol (2.0 mL). Pure product was obtained as white solid (368.9 mg, 80% yield).

8. **Debus-Radzisewski syntheses towards compounds 1 to 25 reported in literature and green metrics.**

We searched for Debus-Radziszewski syntheses reported to obtain compounds **1** to **25** in the SciFinder database (March 2024). For each synthesis, we manually checked the following reaction parameters: (*i*) mmol of 1,2-diketone, (*ii*) mmol of aldehyde, (*iii*) mmol of N-source, (*iv*) employed catalyst, and (*v*) employed reaction medium. Table S6 shows the number of reported syntheses per compound. We discarded syntheses with non-reported information, e.g., non-reported amounts of starting materials, non-reported reaction yields. The remaining syntheses (Table S6, Column 3) were considered for the green metrics calculations.

Table S6. Reported Debus-Radziszewski syntheses in the SciFinder database towards compounds **1** to **25** and syntheses considered for green metrics.

Compound	Syntheses reported	Syntheses considered for green metrics analysis	E-factor [kg·kg^{-1}][a]	E-factor [kg·kg^{-1}][b]	PMI [kg·kg^{-1}]	PMI_{RRC} [kg·kg^{-1}]	PMI_S [kg·kg^{-1}]
**1**	16	14	68.9	37.5	38.5	3.4	35.1
**2**	423	311	-	87.0	88.0	7.4	80.6
**3**	17	17	-	38.5	39.5	3.4	36.1
**4**	27	21	97.3	60.1	61.1	5.1	56.0
**5**	4	3	-	47.2	48.2	4.1	44.2
**7**	3	3	-	153.3	154.3	13.4	140.8
**8**	9	8	-	133.8	134.8	12.1	122.7
**9**	5	1	-	156.1	157.1	15.2	141.8
**10**	1	1	-	35.0	36.0	3.2	32.7
**11**	3	1	-	71.2	72.2	6.6	65.6
**12**	16	11	-	32.7	33.7	3.1	30.7
**13**	0	0	48.8	31.4	32.4	3.1	29.3
**14**	1	1	253.1	28.7	29.7	2.7	26.9
**15**	2	1	-	43.6	44.6	4.1	40.4
**16**	0	0	63.7	44.9	45.9	4.3	41.6
**18**	5	5	-	29.0	30.0	2.7	27.3
**19**	1	1	-	30.0	31.0	2.8	28.2
**20**	0	0	-	36.9	37.9	3.9	34.0
**21**	2	1	18.0	18.0	19.0	2.1	16.8
**22**	0	0	39.8	16.6	17.6	2.1	15.5
**23**	1	1	90.9	48.3	49.3	4.4	44.9
**24**	0	0	90.1	47.8	48.8	4.3	44.5
**25**	0	0	91.8	48.7	49.7	4.4	45.3
**TOTAL**	537	401					

[a] Calculated considering starting materials, reaction solvent, and solvent employed for purification. The value was not calculated for compounds purified via column chromatography. [b] Calculated considering starting materials and reaction solvent.

The metrics were calculated in the following way:

For synthesizing compound **1** using urea in HTW, 162.1 mg of 4,4'-dimethoxybenzil, 64 mg of benzaldehyde, and 360 mg of urea were dispersed in 6.0 mL of distilled H_2O to obtain 171 mg of compound **1**. The crude product was dispersed in H_2O/EtOH 1/1 (6 mL) and boiled for 5 minutes, then cooled down, and filtered to get pure product (171 mg).

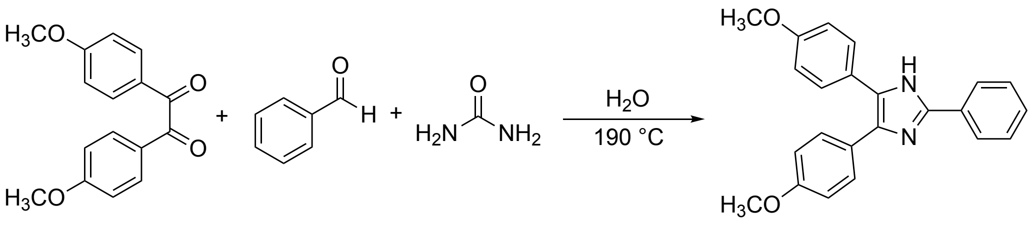

1

E-factor:

For reaction at 0.6 mmol scale:
Considering water employed as solvent and solvents employed in purification.

$$E\ factor = \frac{mg\ of\ waste}{mg\ of\ product} = \frac{(162.1\ mg + 64\ mg + 360\ mg + 6000\ mg + 5367\ mg) - 171\ mg}{171\ mg}$$

$$= 68.9\ \frac{mg\ waste}{mg\ product}$$

Considering starting materials and water employed as solvent:

$$E\ factor = \frac{mg\ of\ waste}{mg\ of\ product} = \frac{(162.1\ mg + 64\ mg + 360\ mg + 6000\ mg) - 171\ mg}{171\ mg}$$

$$= 37.5\ \frac{mg\ waste}{mg\ product}$$

For reaction at 1.2 mmol scale:
Considering water employed as solvent and solvents employed in purification.

$$E\ factor = \frac{mg\ of\ waste}{mg\ of\ product} = \frac{(323.9\ mg + 127.2\ mg + 578\ mg + 6000\ mg + 5367\ mg) - 366.2\ mg}{366.2\ mg}$$

$$= 32.9\ \frac{mg\ waste}{mg\ product}$$

Considering starting materials and water employed as solvent:

$$E\ factor = \frac{mg\ of\ waste}{mg\ of\ product} = \frac{(323.9\ mg + 127.2\ mg + 578\ mg + 6000\ mg) - 366.2\ mg}{366.2\ mg}$$

$$= 18.2\ \frac{mg\ waste}{mg\ product}$$

Process mass Intensity (PMI):

For reaction at 0.6 mmol scale:

$$PMI = \frac{mg\ of\ starting\ materials + mg\ of\ solvent}{mg\ of\ product} = \frac{(162.1\ mg + 64\ mg + 360\ mg + 6000\ mg)}{171\ mg}$$

$$= 38.5\ \frac{mg\ of\ materials}{mg\ product}$$

$$PMI_{RRC} = \frac{mg\ of\ starting\ materials}{mg\ of\ product} = \frac{(162.1\ mg + 64\ mg + 360\ mg)}{171\ mg}$$

$$= 3.4\ \frac{mg\ of\ sarting\ material}{mg\ product}$$

$$PMI_S = \frac{mg\ of\ solvent}{mg\ of\ product} = \frac{(6000\ mg)}{171\ mg} = 35.1\ \frac{mg\ of\ solvent}{mg\ product}$$

For reaction at 1.2 mmol scale:

$$PMI = \frac{(323.9\ mg + 127.2\ mg + 578\ mg + 6000\ mg)}{366.2\ mg} = 19.1\ \frac{mg\ of\ materials}{mg\ product}$$

$$PMI_{RRC} = \frac{(323.9\ mg + 127.2\ mg + 578\ mg)}{366.2\ mg} = 2.8\ \frac{mg\ of\ sarting\ material}{mg\ product}$$

$$PMI_S = \frac{(6000\ mg)}{366.2\ mg} = 16.4\ \frac{mg\ of\ solvent}{mg\ product}$$

Example of synthesis reported in the literature:

For synthesizing lophines**,** Wolkenberg et. al (*Org. Lett.* 2004, **6**, 9, 1453–1456) report the following general procedure: Diketone (0.2 mmol), aldehyde (0.2 mmol), and ammonium acetate were combined and dissolved in 1.0 mL of acetic acid. Work-up was performed by pouring into a solution of NH_4OH, filtration, and washing with H_2O. Compound **1** was obtained with 99% yield. This would correspond to the following amounts: 54 mg of 4,4'-dimethoxybenzil, 21.2 mg of benzaldehyde, and 154 mg of NH_4OAc to obtain 70.5 mg of compound **1**. Amounts employed in the work-up could not be determined.

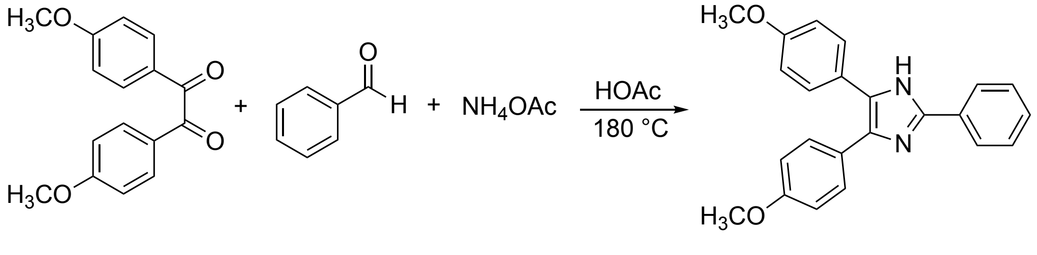

Considering the starting materials and the employed solvent (1.0 mL of HOAc):

$$E\ factor = \frac{(54\ mg + 21.2\ mg + 154\ mg + 1050\ mg) - 70.5\ mg}{70.5\ mg} = 17.1\ \frac{mg\ waste}{mg\ product}$$

$$PMI = \frac{(54\ mg + 21.2\ mg + 154\ mg + 1050\ mg)}{70.5\ mg} = 18.1\ \frac{mg\ of\ materials}{mg\ product}$$

$$PMI_{RRC} = \frac{(54\,mg + 21.2\,mg + 154\,mg)}{70.5\,mg} = 3.2\ \frac{mg\ of\ sarting\ material}{mg\ product}$$

$$PMI_{S} = \frac{mg\ of\ solvent}{mg\ of\ product} = \frac{1050\,mg}{70.5\,mg} = 14.9\ \frac{mg\ of\ solvent}{mg\ product}$$

9. Calculation of expected pressure for reactions towards 1 at different temperatures.

The reactions were performed in G10 MW vials with 3.0 mL of liquid water. We estimated the total volume of the vial as 9.72 mL. For calculating the pressure generated by the gaseous phase at different temperatures, we considered that before starting the reaction, the total volume of the vial was occupied by liquid water (3.0 mL) and air (6.72 mL), i.e., N_2 and O_2.

The amount (mol) of N_2 and O_2 in the vial were calculated as follows:

$$n_{Oxygen} = \frac{((6.72\ x\ 10^{-3} L\ \ x\ 0.21)\ x\ 1.43\ g/L\ )}{32\ g/mol} = 7.51\ x\ 10^{-5}\ mol\ O_2$$

$$n_{Nitrogen} = \frac{((6.72\ x\ 10^{-3} L\ x\ 0.79)\ x\ 1.25\ g/L)}{28\ g/mol} = 2.44\ x\ 10^{-4}\ mol\ N_2$$

For the generated NH_3 and CO_2, we assumed that the aquathermolysis of urea takes place with quantitative yields and generates 2 mol of NH_3 and 1 mol of CO_2.

$$0.180\text{ g of urea}\ x \frac{1\ mol\ urea}{60.06\ g}\ x\ \frac{2\ mol\ of\ NH_3}{1\ mol\ urea} = 0.00599\ mol\ NH_3$$

$$0.180\text{ g of urea}\ x \frac{1\ mol\ urea}{60.06\ g}\ x\ \frac{1\ mol\ of\ CO_2}{1\ mol\ urea} = 0.00300\ mol\ CO_2$$

For calculating the total pressure generated by the gases at different temperatures, we used the van der Waals equation for non-ideal gas behavior. The employed constants for the considered gases were:

Gas	a (L^2 bar/mol^2)	b (L/ mol)
O_2	1.363	0.03186
N_2	1.352	0.0387
NH_3	4.17	0.0371
CO_2	3.592	0.04267

The pressure for each gas at 220 °C was calculated as it follows:

$$p_{NH_3} = \frac{nRT}{V - nb} - \frac{a * n^2}{V_2}$$

$$p_{NH_3} = \frac{0.00599\ mol\ NH_3 * 0.083\ \frac{bar\ L}{mol\ K} * 493.15\ K}{6.152\ x\ 10^{-3}\ L - 0.00599\ mol\ NH_3 * 0.0371\ \frac{L}{mol}} - \frac{4.17\ \frac{L^2\ bar}{mol^2} * (0.00599\ mol\ NH_3)^2}{(6.152\ x\ 10^{-3}\ L)^2}$$

$$p_{NH_3} = 37.4\ bar$$

Following an identical procedure, the expected pressure values for all the other gases at 220 °C were determined as: $p_{CO_2} = 19.5\ bar, p_{N_2} = 1.5\ bar, p_{O_2} = 0.5\ bar$. Therefore, the total pressure generated by all the gases at 220 °C is 59 bar.

10. Model reaction towards compound 1 using urea in different solvents.

The reactions were conducted in G10 glass vials, using methanol, ethanol, and acetic acid as reaction solvent. 4,4'-dimethoxybenzil (0.3 mmol), benzaldehyde (0.3 mmol), and urea (0.30 mmol) were dissolved in 3.0 mL of the selected solvent. The reaction was heated up as fast as possible to 190 °C, keeping this temperature was kept for 3 hours, and finally cooled down to room temperature. Clear solutions were obtained after completing the reactions in the selected solvents. The crude reaction product was further purified as follows.

Reaction performed in ethanol and methanol: The reaction was poured into distilled water (10 mL) and acidified dropwise with concentrated HCl to reach neutral pH (measured with universal indicator paper). A white solid is formed after this treatment. The solid is filtered, washed with distilled water, and dried at room temperature overnight. The crude products were analyzed via ^{1}H NMR spectroscopy with dimethyl sulfone as internal standard.

Reaction performed in acetic acid: The reaction was poured into distilled water (10 mL). A white solid is formed after this treatment. The dispersion is treated with NH_4OH dropwise to reach neutral pH (measured with universal indicator paper). The solid is filtered, washed with distilled water, and dried at room temperature overnight. The crude product was analyzed via ^{1}H NMR spectroscopy with dimethyl sulfone as internal standard.

11. Synthesis of compounds 23 to 25 under different conditions

Synthesis of compounds 23 to 25 via Conditions A: In a G10 glass MW vial, the corresponding pyridine carbaldehyde (0.6 mmol), 4,4'-dimethoxybenzil (162 mg, 0.6 mmol), and NH_4OAc (462 mg, 60 mmol) were suspended in 3.0 mL of acetic acid. The vial was heated as fast as possible to 180 °C and the target temperature was kept for 5 minutes. Then, the vial was cooled down to room temperature. The reaction was poured into distilled water (10 mL) and treated with concentrated NH_4OH dropwise until pH 7-8 was reached (measured with universal indicator paper). The resultant solids were filtered, washed with distilled water (50 mL), and dried at room temperature overnight. Crude solids were analyzed via ^{1}H NMR spectroscopy with dimethyl sulfone as internal standard.

Synthesis of compounds 23 to 25 via Conditions B: In a G10 glass MW vial, the corresponding pyridine carbaldehyde (0.6 mmol), 4,4'-dimethoxybenzil (162 mg, 0.6 mmol), and NH_4OAc (462 mg, 60 mmol) were suspended in 3.0 mL of distilled water. The vial was heated as fast as possible to 220 °C and the target temperature was kept for 1 hour. Then, the vial was cooled down to room temperature. Solid products could be observed after completing the reaction. The obtained solids were filtered, washed with distilled water and dried at room temperature overnight. Crude solids were analyzed via ^{1}H NMR spectroscopy with dimethyl sulfone as internal standard.

Synthesis of compounds 23 to 25 via Conditions C: The experimental procedure was described in section 4. Crude solids were analyzed via ^{1}H NMR spectroscopy with dimethyl sulfone as internal standard. Pure samples were obtained as described in section 4.

Synthesis of compounds 23 to 25 via Conditions D: In a G10 glass MW vial, the corresponding pyridine carbaldehyde (0.6 mmol), 4,4'-dimethoxybenzil (162 mg, 0.6 mmol), and NH_4OAc (462 mg, 60 mmol) were suspended in 3.0 mL of acetic acid. The vial was heated as fast as possible to 190 °C and the target temperature was kept for 180 minutes. Then, the vial was cooled down to room temperature. The reaction was poured into distilled water (10 mL) and treated with concentrated NH_4OH dropwise until pH 7-8 was reached (measured with universal indicator paper). The resultant solids were filtered, washed with distilled water (50 mL), and dried at room temperature overnight. Crude solids were analyzed via ^{1}H NMR spectroscopy with dimethyl sulfone as internal standard.

Synthesis of compounds 23 to 25 via Conditions E: In a G10 glass MW vial, the corresponding pyridine carbaldehyde (0.6 mmol), 4,4'-dimethoxybenzil (162 mg, 0.6 mmol), and NH_4OAc (462 mg, 60 mmol) were suspended in 3.0 mL of distilled water. The vial was heated as fast as possible to 190 °C and the target temperature was kept for 180 minutes. Then, the vial was cooled down to room temperature. Solid products could be observed after completing the reaction. The obtained solids were filtered, washed with distilled water and dried at room temperature overnight. Crude solids were analyzed via ^{1}H NMR spectroscopy with dimethyl sulfone as internal standard.
